# 1,2,3-Triazole Derivatives as Novel Antifibrinolytic Drugs

**DOI:** 10.3390/ijms232314942

**Published:** 2022-11-29

**Authors:** Oriol Bosch-Sanz, Yvette Rabadà, Xevi Biarnés, Javier Pedreño, Luis Caveda, Mercedes Balcells, Jordi Martorell, David Sánchez-García

**Affiliations:** 1 IQS School of Engineering, Universitat Ramon Llull, Via Augusta 390, 08017 Barcelona, Spain; 2 Institute of Medical Engineering and Science, Massachusetts Institute of Technology, 77 Massachusetts Ave, Cambridge, MA 02139, USA; 3 Laboratory of Biochemistry, Institut Químic de Sarrià, Universitat Ramon Llull, Via Augusta 390, 08017 Barcelona, Spain; 4 Alxerion Biotech, 245 First St, Riverview II, 18th Floor, Cambridge, MA 02142, USA; 5 Grup d’Enginyeria de Materials, Institut Químic de Sarrià, Universitat Ramon Llull, Via Augusta 390, 08017 Barcelona, Spain

**Keywords:** fibrinolysis, plasminogen, plasmin, tPa, triazole, oxadiazolone, antifibrinolytic

## Abstract

Fibrinolysis is a natural process that ensures blood fluidity through the removal of fibrin deposits. However, excessive fibrinolytic activity can lead to complications in different circumstances, such as general surgery or severe trauma. The current antifibrinolytic drugs in the market, aminocaproic acid (EACA) and tranexamic acid (TXA), require high doses repetitively to maintain their therapeutic effect. These high doses are related to a number of side effects such as headaches, nasal symptoms, or gastrointestinal discomfort and severely limit their use in patients with renal impairment. Therefore, the discovery of novel antifibrinolytics with a higher specificity and lower dosage could vastly improve the applicability of these drugs. Herein, we synthesized a total of ten compounds consisting of a combination of three key moieties: an oxadiazolone, a triazole, and a terminal amine. The IC_50_ of each compound was calculated in our clot lysis assays, and the best candidate (**1**) provided approximately a 2.5-fold improvement over the current gold standard, TXA. Molecular docking and molecular dynamics were used to perform a structure–activity relationship (SAR) analysis with the lysine binding site in the Kringle 1 domain of plasminogen. This analysis revealed that 1,2,3-triazole was crucial for the activity, enhancing the binding affinity through pi–pi stacking and polar interactions with Tyr72. The results presented in this work open the door to further investigate this new family as potential antifibrinolytic drugs.

## 1. Introduction

Fibrinolysis is a natural process that consists of the breakdown of blood clots due to the degradation of fibrin fibers. Under physiological conditions, both coagulation and fibrinolysis are finely regulated by a series of substrates, activators, inhibitors, cofactors, and receptors [1,2,3]. The activation of coagulation ultimately promotes the transformation of fibrinogen to fibrin, which constitutes the main structural protein of blood clots [1]. The activation of fibrinolysis allows for the localized, timely removal of ongoing or acutely induced fibrin deposits. These coordinated molecular events ensure blood fluidity while preventing blood loss [4]. Therefore, fibrinolysis constitutes a necessary control mechanism within the hemostatic system [5,6].

However, excessive fibrinolytic activity can be dangerous and has been related to certain disorders and complications. For example, it has been linked to heavy menstrual bleeding [7] and complications during dental surgery for hemophiliac patients [8] as well as excessive post-partum hemorrhage [9]. Reducing fibrinolysis can also assist in minimizing blood loss during general surgery [10,11] or severe trauma.

Severe trauma has been reported to result in a massive activation of the fibrinolytic system [12], contributing to the development of acute traumatic coagulopathy and exacerbation of bleeding. Hemorrhage represents the leading cause of preventable death due to injury, and excessive fibrinolysis is a contributing factor in up to 40% of all deaths from trauma [13]. Existing antifibrinolytic drugs have been proven to reduce mortality in trauma patients in clinical trials, such as the CRASH-2 trial [14]. Consequently, antifibrinolytic drugs such as tranexamic acid (TXA) have been adopted into trauma practice guidelines worldwide [15].

The lysis of fibrin fibers is performed by plasmin, a well-known serine protease. Its inactive zymogen plasminogen (PLG) binds to fibrin, where it is activated into plasmin by plasminogen activators, namely tissue plasminogen activator (tPa) and urokinase plasminogen activator (uPa) [16]. This process enables localized activity in the presence of fibrin-based blood clots. While tPA is a weak activator of PLG in the absence of fibrin, its catalytic efficiency for PLG activation is enhanced by at least two orders of magnitude in the presence of fibrin [1]. This enhanced activity is possible due to the attachment of PLG to fibrin through the lysine binding sites (LBS) present in its Kringle domains, the most relevant one being Kringle 1 (K1).

**Figure 1:**
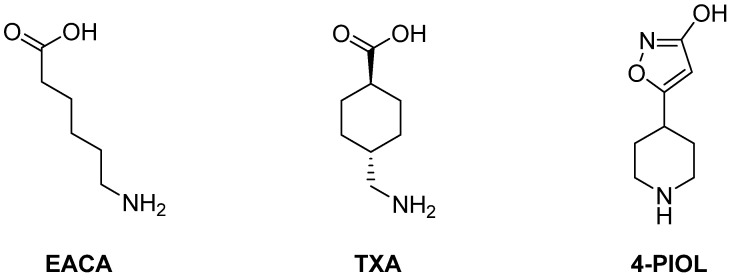
Previously studied small molecules with antifibrinolytic activity by inhibition of the lysine binding sites of plasminogen.

Inhibiting the interaction of the LBS in K1 with fibrin is therefore a viable mechanism to slow down the activation of inactive plasminogen to active plasmin and to ultimately slow down fibrinolysis. Lysine analogues—such as ε-aminocaproic acid (EACA) and tranexamic acid (TXA)—have been vastly studied [17,18,19,20,21] (Figure 1). It has been reported that they are able to form complexes with the LBS of K1-K5, although the highest affinity is observed in the case of K1 [22]. Consequently, small-molecule lysine analogues have been proven to inhibit fibrinolysis [23,24,25].

The above-mentioned lysine analogues, EACA and TXA, are the active ingredients of different FDA-approved antifibrinolytic drugs, such as Amicar™ (EACA), Cyclokapron™ (TXA) or Lysteda™ (TXA). Although commonly used, they all require very high doses (Amicar™: 1 g per hour [26]; Cyclokapron™: 25 mg/kg four times per day [27]; Lysteda™: 3 × 1.3 g per day [7]), which are associated with a high frequency of side effects such as headaches, nasal symptoms, or back, abdominal, and muscle pain [7]. Additionally, gastrointestinal discomfort, including nausea, vomiting, and diarrhea, occur in 30% of patients treated orally with EACA or TXA [28,29]. Hypotension has been observed when it is intravenously administered too rapidly [30]. The need for high doses to obtain sustained effects causes heavy limitations in patients with prior renal malfunctions. Consequently, TXA is contraindicated in patients with severe renal impairment. In cases of mild to moderate renal impairment, the dosage is reduced according to the serum creatinine level [31].

Different attempts have been made to overcome these limitations. The approval in 1998 of aprotinin, a direct plasmin inhibitor, provided a substantial improvement to the high dosage demands of lysine analogues. Unfortunately, post-operative risks identified in post-market surveillance [32,33] led to its withdrawal in 2007 by the FDA and the EMA. More recently, a piperidine derivative named 4-PIOL (Figure 1) was described by Böstrom et al. as a powerful plasminogen binding inhibitor with an IC_50_ of 0.8 µM [34], which is four times more active than TXA. However, its affinity towards the GABA_A_ receptor, which controls the majority of inhibitory signaling in the nervous central system [35], remains an issue regarding possible neurological side effects [36]. Some attempts have been made to improve its selectivity by adding additional residues to the piperidine ring [37], subsequently complicating the synthetic pathway by adding additional chiral centers. Other piperidine derivatives with very high potency (6–15 nM) have been described by Bayer in different patents [38,39]. However, their complex structure and long synthetic pathway of over 15 steps preclude them from being scalable drugs. In fact, no further development has been made public regarding these compounds. Despite further attempts [40,41] to obtain more potent and selective compounds, no other antifibrinolytics exist currently in the market.

**Figure 2:**
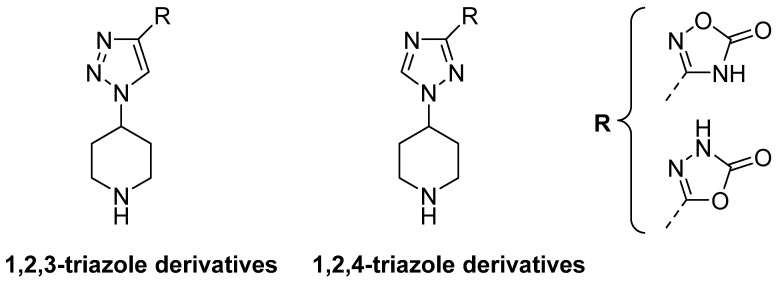
General structure for the hypothesized components, which include a piperidine ring, a triazole ring, and an oxadiazolone ring.

Traditional lysine analogues (EACA, TXA) present terminal amine and carboxylic moieties. 4-PIOL, however, achieved an improved activity by combining a piperidine moiety and an additional aromatic ring. Introducing rigid rings in the ligand structure is a common way to increase potency and selectivity [42], reducing entropic energy penalties in ligand–receptor interactions. In order to bind to the LBS, compounds need to interact with both the basic and acidic regions of the K1 pocket. The example of 4-PIOL is a valid example, where activity was improved by rigidifying the molecular structure. It introduced a piperidine instead of a linear amine, and an isoxazole ring instead of a carboxylic acid. An additional ring would simultaneously increase the rigidity of the molecule and its size, potentially reducing its affinity for the GABA_A_ receptor. Therefore, we hypothesized that compounds containing a terminal piperidine to interact with the acidic region, a triazole ring as a linker, and an oxadiazolone ring to interact with the basic region could provide new viable antifibrinolytic candidates (Figure 2).

This work presents the synthesis of ten compounds, including variations to the hypothesized structure, a thorough an analysis of their activity and structure–activity relationship, as well as a comparison with approved antifibrinolytic active ingredients (EACA, TXA).

## 2. Results

### 2.1. Synthesis

**Scheme 1:**
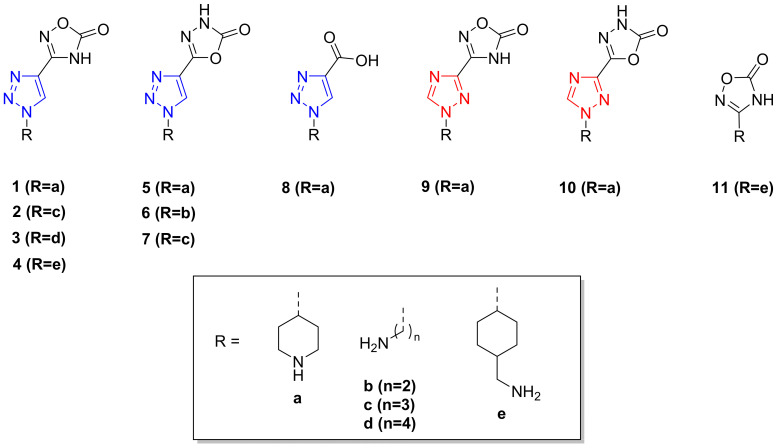
Studied compounds for their antifibrinolytic activity.

Two families of triazole derivatives were synthesized: 1,2,3-triazole and 1,2,4-triazole. Position 1 was substituted either with a piperidine, a linear terminal amine, or a cyclohexyl methanamine. Position 4 (1,2,3 disposition) or 3 (1,2,4 disposition) of the triazole ring was substituted with either a 1,2,4 oxadiazolone (**1**-**4**, **9**), a 1,3,4-oxadiazolone (**5**-**7**, **10**), or a carboxylic acid functionality (**8**). An additional compound (**11**) was synthesized, which combined a 1,2,4-oxadiazolone and a cyclohexyl methanamine (Scheme 1).

**Scheme 2:**
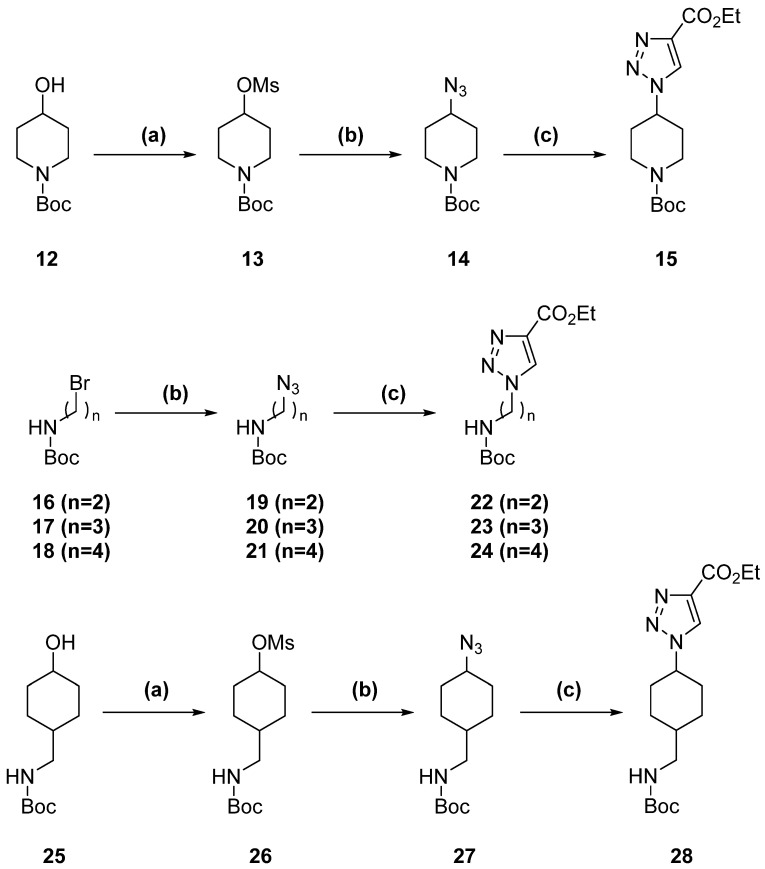
1,2,3-triazole formation. (**a**) TEA, MsCl, DCM, 0–5 °C to 23 °C, 2 h. (**b**) NaN_3_, DMF, 80 °C, 8 h. (**c**) Ethyl propiolate, CuI, ACN, 23 °C, 12 h.

1,2,3-triazole derivatives were synthesized by a Cu(I)-catalyzed cycloaddition between an azide and ethyl propiolate. Their azide precursor was obtained through two different methods. For compounds based on linear amines, their respective bromide derivatives were transformed directly into azide by treatment with NaN_3_ (Scheme 2). For the piperidine and cyclohexyl methanamine derivatives, hydroxyl groups were activated into mesylates by treatment with methanesulfonyl chloride in DCM. Subsequently, the mesylate groups were converted into azide by treatment with NaN_3_.

**Scheme 3:**
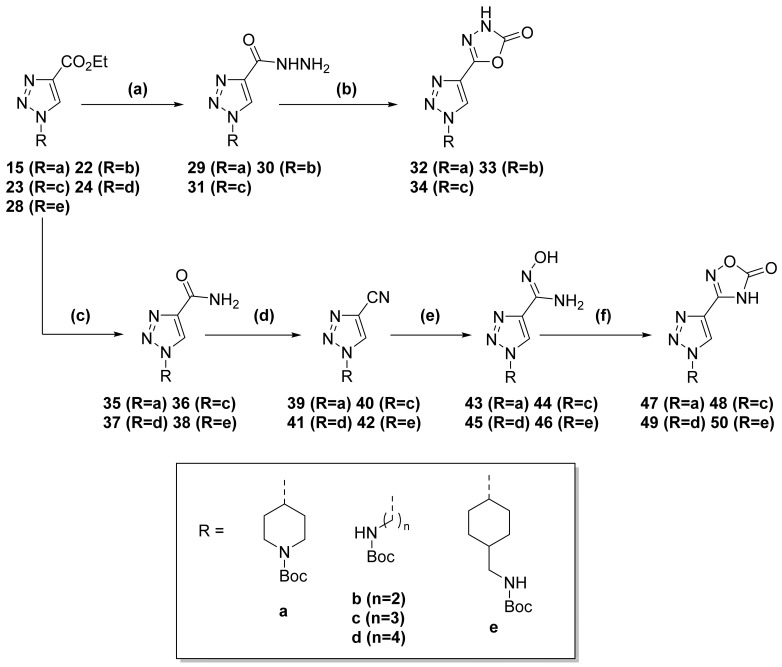
Oxadiazolone formation for 1,2,3-triazole derivatives. (**a**) Hydrazine hydrate, *n*-butanol, reflux, 3 h. (**b**) CDI, DBU, ACN, reflux, 15 h. (**c**) NH_3_, MeOH, 23 °C, 12 h. (**d**) TEA, TFAA, DCM, 0–5 °C to 23 °C, 5 h. (**e**) NH_2_OH·HCl, NaHCO_3_, MeOH, reflux, 14 h. (**f**) CDI, DBU, ACN, reflux, 15 h.

To obtain the final 1,3,4-oxadiazolone derivatives (compounds **5**, **6**, **7**), ester groups were first transformed into the corresponding hydrazides by treatment with hydrazine hydrate in refluxing *n*-butanol. Subsequently, the 1,3,4-oxadiazolone ring was formed through cycloaddition with CDI in the presence of DBU in acetonitrile (Scheme 3).

Alternatively, to obtain the 1,2,4-oxadiazolone derivatives (compounds **1**, **2**, **3**, **4**), ester groups were transformed into their corresponding amide by treatment with methanolic ammonia at room temperature. The amide was later dehydrated in the presence of TFAA to provide the respective nitrile. Subsequent reflux with hydroxylamine hydrochloride and sodium bicarbonate in MeOH yielded the corresponding hydroxylamine. Finally, the 1,2,4-oxadiazolone ring was formed by reaction with CDI and DBU in ACN (Scheme 3).

**Scheme 4:**
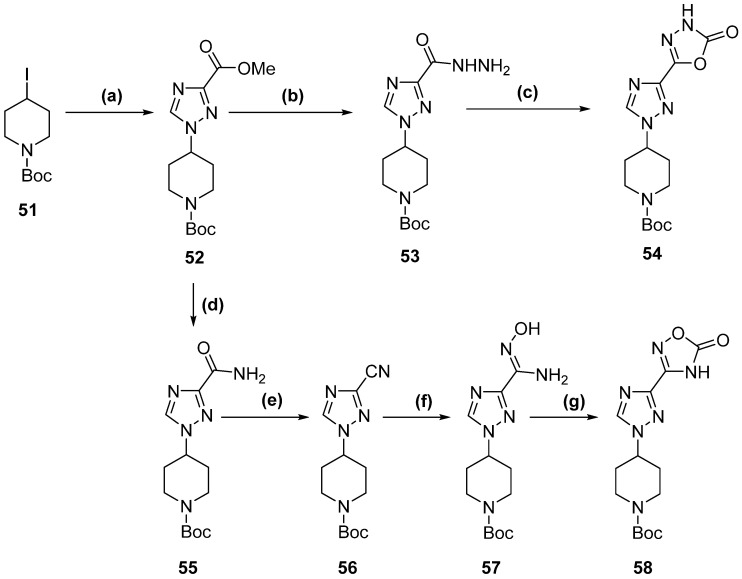
1,2,4-triazole derivatives complete synthetic pathway. (**a**) methyl 1*H*,1,2,4-triazole-3-carboxylate, NaH, DMF, 70 °C, 48 h. (**b**) Hydrazine hydrate, *n*-butanol, reflux, 3 h. (**c**) CDI, DBU, ACN, reflux, 15 h. (**d**) NH3, MeOH, 23 °C, 12 h. (**e**) TEA, TFAA, DCM, 0–5 °C to 23 °C, 5 h. (**f**) NH_2_OH·HCl, NaHCO_3_, MeOH, reflux, 14 h. (**g**) CDI, DBU, ACN, reflux, 15 h.

For the 1,2,4-triazole derivatives, the ester–triazole compound was obtained through alkylation in DMF at 70 °C for 48 h using NaH as a base (Scheme 4). The following steps, both for the 1,3,4-oxadiazolone (compound **10**) and 1,2,4-oxadiazolone derivatives (compound **9**), were identical to the ones described for the 1,2,3-triazole derivatives (Scheme 3).

**Scheme 5:**
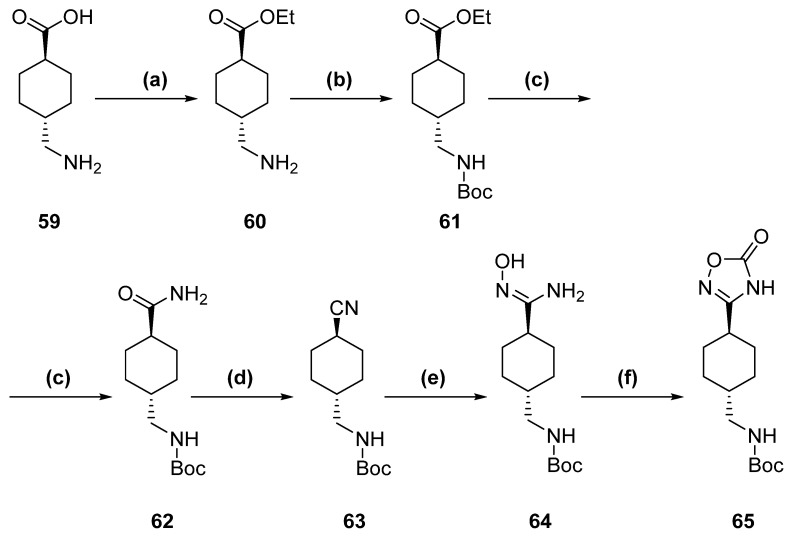
Tranexamic acid derivative complete synthetic pathway. (**a**) TsCl, EtOH, reflux, 2 h. (**b**) Boc_2_O, TEA, MeOH, 23 °C, 12 h. (**c**) NH_3_, MeOH, 85 °C (sealed vial), 5 days. (**d**) TEA, TFAA, DCM, 0–5 °C to 23 °C, 5 h. (**e**) NH_2_OH·HCl, NaHCO_3_, MeOH, reflux, 14 h. (**f**) CDI, DBU, ACN, reflux, 15 h.

Compound **11** was synthesized using tranexamic acid as the starting material (Scheme 5). First, it was treated with thionyl chloride in ethanol to provide the ester. Subsequently, the terminal amine was protected with a Boc group. The conversion of ester to amide was achieved by treatment in methanolic ammonia at 85 °C for 5 days inside an autoclave. The following steps were again identical to those explained for the 1,2,3-triazole (Scheme 3) and 1,2,4-triazole (Scheme 4) derivatives.

Final compounds were always obtained as hydrochloride salts after treating their protected precursors with methanolic HCl at room temperature for 2 h. The set of molecules tested in this study are shown in Scheme 1.

### 2.2. Clot Lysis Assays

**Figure 3:**
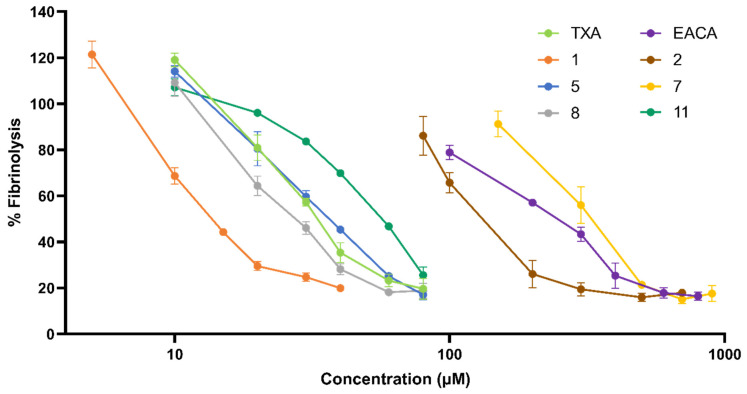
Dose–response curve for each molecule represented as a percentage of fibrinolysis vs. concentration (µM) of inhibitor measured in plasma clot lysis assays. Compounds not included in this graph showed undetectable activity below 1000 µM.

The activity for each compound at different concentrations was assessed through in vitro clot lysis assays performed in human plasma. The results portrayed in Figure 3 show the relation between activity and concentration for each compound. IC_50_ values (Table 1) were calculated as the concentration at which fibrinolytic activity was 50% of the control value.

The data show that compound **1** provided the highest activity among the tested products with an IC_50_ less than half that of TXA, the current gold standard of antifibrinolytic drugs. The chemical structure of **1** (Scheme 1) consisted of a piperidine, a 1,2,3-triazole ring, and a 1,2,4-oxadiazolone. In compound **5**, the 1,2,4-oxadiazolone was replaced with a 1,3,4-oxadiazolone. This change caused its IC_50_ to increase 2.5-fold, presenting a similar value to TXA. This decrease in activity indicated a higher affinity for 1,2,4-oxadiazolone when compared to 1,3,4-oxadiazolone. Accordingly, compound **2** also provided a higher activity than **7**.

Compounds **2-3** and **6-7** all presented the same structures as **1** and **5**, except for the presence of a piperidine ring. This moiety was replaced with linear amines of different lengths. In all cases, the presence of a linear amine severely decreased the activity. This observation highlighted the importance of the bulkiness and rigidity of the piperidine ring compared to a linear chain. Compounds with a propylamine residue (**2**, **7**) showed a 10-fold increase in the IC_50_ when compared to their piperidine counterparts. For compounds with either shorter (**6**) or longer (**3**) linear chains, no activity could be measured below 1000 µM. In addition, a lack of activity was observed for compound **4**, which presented the structure of compound **1** with a cyclohexyl methanamine residue instead of propylamine.

The possibility of maintaining a carboxylic acid (present in TXA) instead of an oxadiazolone ring was also considered. Compound **8** presented the structure of **1**, with a carboxylic acid moiety instead of the 1,2,4-oxadiazolone. The results showed a 2-fold decrease in activity whilst still conserving a slightly higher potency than compound **5**. In addition, compound **11** was synthesized as a derivative of TXA, having its carboxylic acid substituted for the 1,2,4-oxadiazolone ring present in compound **1**. The results showed that although a noticeable activity was maintained, it was lower compared to that of TXA.

Regarding the central triazole ring, the results showed that almost all activity was lost when substituting the 1,2,3-triazole (compounds **1** and **5**) for a 1,2,4-triazole (compounds **9** and **10**). This pointed toward the importance of having all three nitrogen atoms aligned on the same side of the molecule.

### 2.3. Computational Analysis

**Figure 4:**
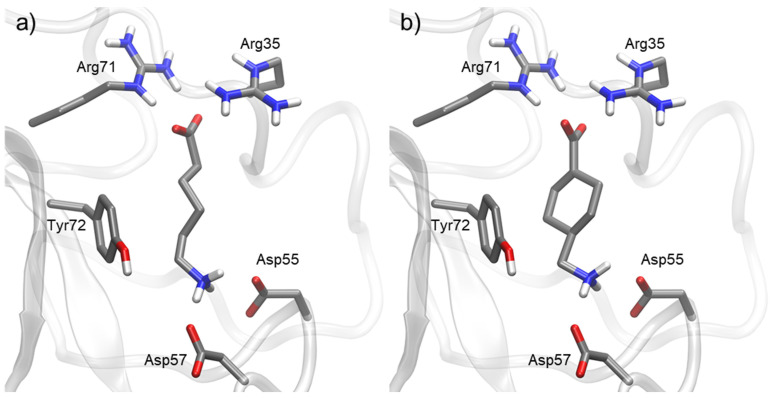
Docking of most representative poses for lysine analogues (**a**) ε-aminocaproic acid (EACA) and (**b**) tranexamic acid (TXA). Docking studies were performed with Kringle 1 lysine binding site (pdb code 1cea) using AutoDock 4.2.

Docking simulations were performed for all the studied compounds against the lysine binding site in the Kringle 1 domain of plasminogen. The binding mode (Figure 4) for lysine analogues, EACA and TXA, was equivalent to what has been reported previously [23]. Their most favorable target–ligand interactions were concentrated in two areas. On one side, the terminal amine could form salt bridges with Asp55 and Asp57. Simultaneously, the carboxylic acid moiety at the opposite end of the molecule could form other salt bridges with Arg35 and Arg71.

**Figure 5:**
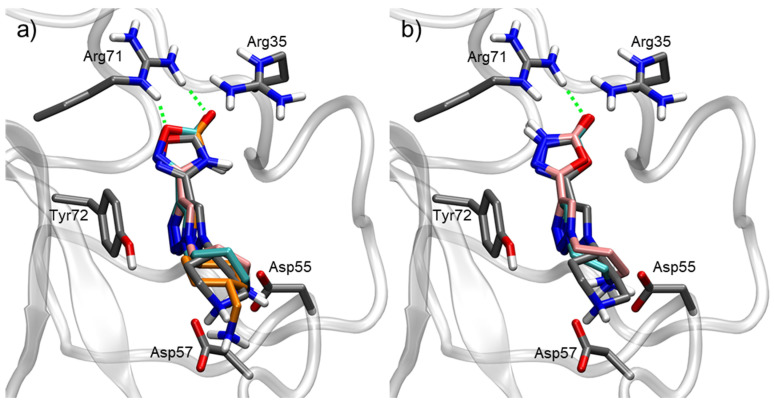
Docking of most representative poses for 1,2,3-triazole derivatives combined with oxadiazolone rings. (**a**) 1,2,4-oxadiazolone compounds: **1** (grey), **2** (pink), **3** (cyan), **4** (orange). (**b**) 1,3,4-oxadiazolone compounds: **5** (grey), **6** (cyan), **7** (pink). Docking studies were performed with Kringle 1 lysine binding site (pdb code 1cea) using AutoDock 4.2. H-bond interactions with the oxadiazolone ring are indicated as green dashed lines.

For compounds derived from 1,2,3-triazole and an oxadiazolone ring, the interactions were slightly different (Figure 5). Similarly to EACA and TXA, the terminal amine interacted with the acidic side of the pocket, where Asp55 and Asp57 reside. The nature of this amine moiety played an important role. Linear amine residues possess a higher flexibility to adapt to the position of Asp amino acids, although their increased entropy also decreases the stability of their interactions. In addition, the length of these moieties is also important to achieve the optimal size for the target pocket.

The oxadiazolone region interacts with the basic side of the pocket. For the 1,2,4-oxadiazolone derivatives, the oxygen atom from the ring enabled the formation of an H-bond with Arg71, while the carbonyl group could also form an H-bond with Arg71 as well as electrostatic interactions with Arg35. This could explain the increased activity observed in the 1,2,4-oxadiazolone derivatives (**1**, **2**) when compared to their 1,3,4-oxadiazolone counterparts (**5**, **7**).

The central part of the molecule, a triazole ring, also played a crucial role for its activity. The aromatic ring allowed for certain pi–pi stacking interactions with Tyr72 (Figure 5), which is not present in the case of TXA and EACA (Figure 4).

**Figure 6:**
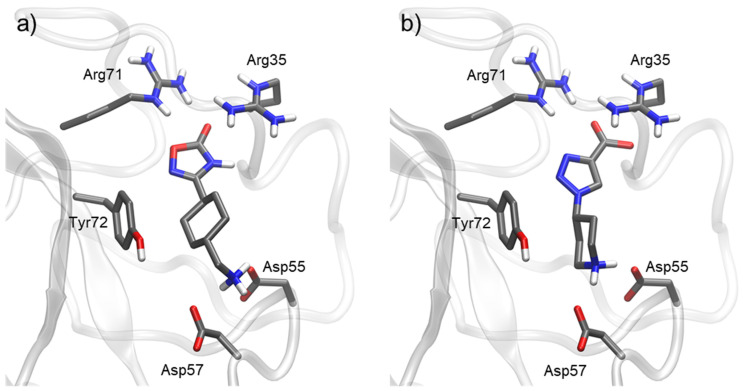
Docking of most representative poses for compounds **11** (**a**) and **8** (**b**). Docking studies were performed with Kringle 1 lysine binding site (pdb code 1cea) using AutoDock 4.2.

For compound **11** (Figure 6), the interactions were similar to those of TXA. They both include a cyclohexyl methanamine moiety that interacts with Asp55 and Asp57. However, the carboxylic acid in TXA was replaced for a 1,2,4-oxadiazolone ring in molecule **11**. This change increased the length of the molecule. However, most importantly, it eliminated the positive charge, therefore removing any possible salt bridging with Arg71 and Arg35. This explained the lower activity of **11** when compared to TXA. When compared to molecule **1**, the absence of a triazole ring in molecule **11** removed the pi–pi stacking interaction with Tyr72. As a result, the activity of compound **11** was four times lower than that of **1**.

In the case of compound **8** (Figure 6), the carboxylic acid formed salt bridges with Arg71 and Arg35, similarly to lysine analogues. As mentioned, carboxylic acid provides a stronger interaction with this basic area of the target than oxadiazolone rings. With this disposition of the molecule, however, pi–pi stacking interaction between the triazole ring and Tyr72 was lost. This explained the lower activity of compound **8** compared to compound **1**.

**Figure 7:**
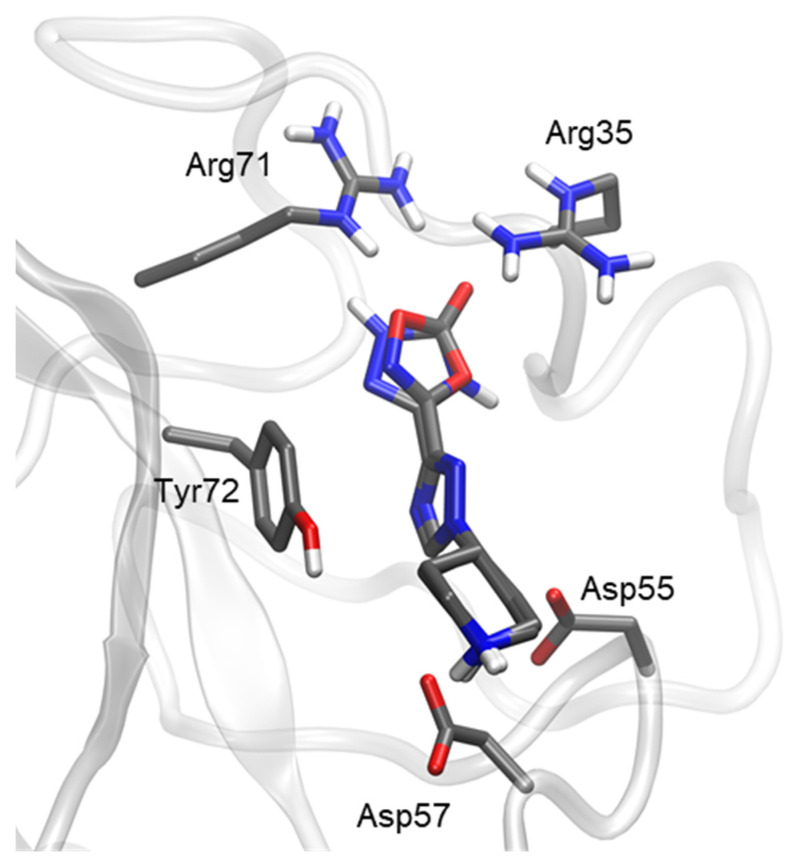
Docking of most representative poses for 1,2,4-triazole derivatives (compounds **9** and **10**). Docking studies were performed with Kringle 1 lysine binding site (pdb code 1cea) using AutoDock 4.2.

The docking poses for compounds **9** and **10** (Figure 7) were very similar to those of **1** and **5**. The interactions of the piperidine moiety, as well as the oxadiazolone ring, with the lysine binding site were maintained. For the triazole ring region, the change in structure from a 1,2,3-triazole to a 1,2,4-triazole did not eliminate the pi–pi stacking interaction with Tyr72. Therefore, docking itself was insufficient to explain the loss of activity of compounds **9** and **10** with respect to **1** and **5**. Molecular dynamics analysis was performed to assess whether the change in the triazole ring in compound **9** could affect the dynamics of the ligand in the lysine binding site.

**Figure 8:**
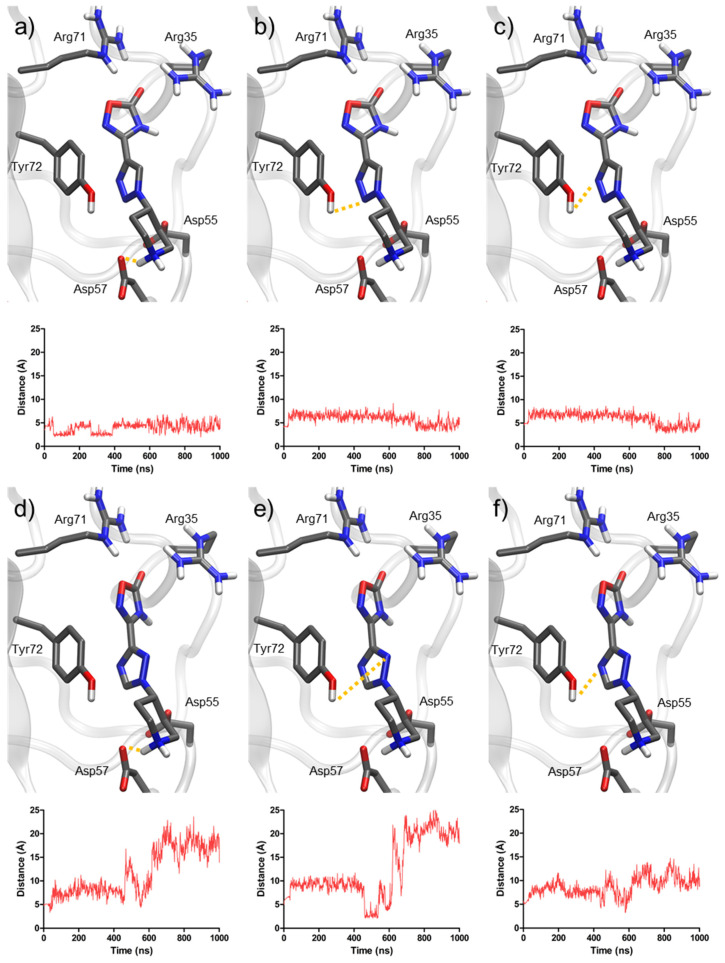
Representation of the most important distances for molecular dynamics simulations of compounds **1** and **9**. Distances for compound **1**: (**a**) piperidine and Asp57, (**b**) triazole (nitrogen N2) and Tyr72, (**c**) triazole (nitrogen N3) and Tyr72. Distances for compound **9**: (**d**) piperidine and Asp57, (**e**) triazole (nitrogen N2) and Tyr72, (**f**) triazole (nitrogen N4) and Tyr72.

The molecular dynamics simulations (Figure 8) showed a clearly different behavior for compounds **1** and **9**. Molecule **1** (1,2,3-triazole) remained inside the binding site throughout the entire simulation time (1000 ns), while molecule **9** (1,2,4-triazole) did not, abandoning it at 500–600 ns.

The distance between piperidine and Asp57 was the most stable throughout the simulations. In the case of compound **1**, it remained around 3 Å during the entire simulation, indicating the presence of an H-bond. For compound **9**, the interaction was weaker, with a distance around 7 Å, until the piperidine suddenly separated at 600 ns, indicating the exit of the molecule from the binding site.

Additionally, the distances between the nitrogen atoms in each triazole ring and Tyr72 (particularly its hydroxyl group) also provided key information. For compound **1**, these distances fluctuated between 3 and 6 Å, which allowed for polar interactions including H-bonds. In the case of compound **9**, these distances were always above 7 Å, until the molecule finally exited the pocket. These results highlighted the role of 1,2,3-triazole in providing anchoring to the binding site, mainly with Tyr72, which 1,2,4-triazole cannot provide.

**Figure 9:**
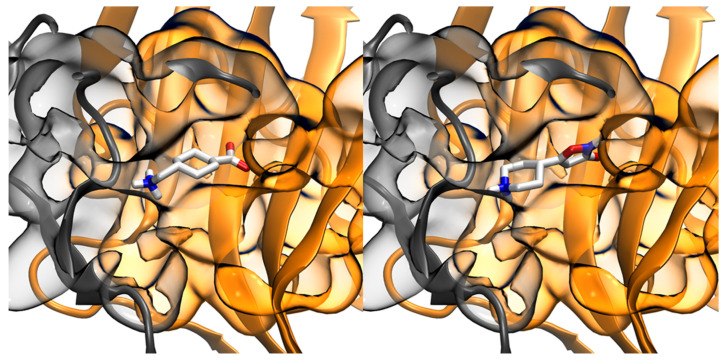
Binding poses with GABA binding pocket of GABA_A_ receptor for tranexamic acid (**left**) and 4-PIOL (**right**). GABA_A_ receptor file obtained from PDB code 6d6u.

The interactions of TXA, 4-PIOL, and compound **1** with the GABA_A_ receptor were analyzed through docking (Figure 9). The results showed viable binding modes for TXA and 4-PIOL inside the GABA binding pocket. This was in alignment with their known affinity towards the GABA_A_ receptor. Although both TXA and 4-PIOL are larger than GABA in terms of molecular surface (Table 2), this increase in size does not preclude them from interacting with the GABA binding pocket. In case of compound **1**, no docking pose was observed in the binding site. This result indicated that the size of molecule **1** (almost 50% more molecular surface than GABA) precluded it from interacting with the enclosed GABA binding site.

## 3. Discussion

Oxadiazole derivatives have been proven relevant in medicinal chemistry, with their anti-inflammatory, analgesic, or antiproliferative [43,44,45] activities having been described. In our compounds, the modified oxadiazolone ring interacted with the arginine residues of the LBS pocket comparably to the carboxylic acid in traditional lysine analogues (EACA and TXA). Interestingly, the 1,2,4-oxadiazolone was more active than a carboxylic acid derivative. Given the basic nature of arginine, the affinity with an acidic moiety could be expected to be higher than with an oxadiazolone. However, the additional bulkiness and rigidity of the ring when compared to a linear chain appeared to positively impact the activity. The size of the oxadiazolone allowed for H-bonds to form between the oxadiazolone and Arg71 and, interestingly, the docking studies showed two potential bonds being formed in the presence of the 1,2,4-oxadiazolone moiety, giving it its highest activity.

The 1,2,3-triazole moiety not only provides a linking scaffold easily prepared through “click” chemistry [46] but has also shown interesting antimicrobial, antiviral, and antitumoral activities in many molecules [47,48]. In our compounds, the 1,2,3-triazole disposition proved to be of critical importance. Against all odds, and against the initial docking results, replacing such a disposition for a 1,2,4-triazole made the drug totally inactive. Only molecular dynamic simulations were able to significantly differentiate both structures, pointing towards the idea of a more stable ligand–target binding. Although docking assays are a relevant tool to identify key interactions for each compound, dynamic simulations provide more complex information. This becomes more relevant in an external binding site, such as the studied K1 domain, which is therefore inherently flexible. Consequently, the different interactions of 1,2,3-triazole and 1,2,4-triazole derivatives with Tyr72 were only observed when studying their behavior in a dynamic system. 1,2,3-triazole demonstrated a higher stabilizing capacity for the pocket, explaining the activity difference between **1** and **9**.

The presence of a terminal piperidine, instead of other amine residues, proved to be crucial for activity, in alignment with the literature [37,49]. On the one hand, substituting the piperidine for a linear amine rendered the molecules inactive or less active, confirming the importance of the bulkiness and rigidity of piperidine ring. Interestingly, however, substituting the piperidine with a cyclohexyl methanamine residue radically decreased the activity, indicating an excessive distance between the terminal amine and oxadiazolone ring. On the other hand, when comparing linear amines, the propylamine residue showed a higher activity than the ethylamine and butylamine residues. This was coherent with the adequacy of piperidine, since propylamine presents the same number of bonds between the triazole ring and the amine group. Therefore, these results reinforced the unicity of piperidine in compounds interacting with the LBS of the K1 domain of plasminogen.

As mentioned in the introduction, other molecules containing piperidine rings have been documented in the literature to provide antifibrinolytic effects. Regarding the molecules disclosed by Bayer [38,39], their scalability is diminished by their complex structures and long synthetic pathways of over 15 steps. In comparison, our two most prominent molecules required only six to eight synthetic steps, thereby presenting a much easier pathway towards a scalable process.

Regarding 4-PIOL, we tested commercially available 4-PIOL and obtained very similar IC_50_ values to those published (0.8 µM [34]), validating our plasma method and making it comparable to the available bibliography. The 4-PIOL in vitro results were hence notably better than those of our family of molecules. However, 4-PIOL’s interference with the GABA_A_ receptor [36] remains its main limitation. We studied in silico the binding capacity of 4-PIOL, TXA, and our most active compound (**1**) towards GABA_A_ receptor. Our docking results indicated that 4-PIOL and TXA bound competitively to the GABA binding pocket, whereas the larger size of our family of compounds precluded the binding to the receptor. This indicated a probable improvement in the selectivity of compound **1** when compared to 4-PIOL. While significant efforts have been performed to correct this selectivity issue in 4-PIOL by adding a substituent in the piperidine ring [37], this adds an additional chiral center and thus substantially complicates the synthetic pathway.

## 4. Materials and Methods

### 4.1. Synthesis

All reagents were purchased from Sigma Aldrich or FluoroChem and were used without further purification. The progress of all reactions was monitored on Merck precoated silica gel plates (with fluorescence indicator UV_254_) using ethyl acetate/cyclohexane as the solvent system. Column chromatography was performed with Merck silica gel 60 (230–400 mesh particle size). Proton (^1^H) and carbon (^13^C) NMR spectra were recorded on a Varian 400 (400 MHz for ^1^H; 100 MHz for ^13^C) using chloroform-d or *d_6_*-DMSO as the solvent. Chemical shifts were given in parts per million (ppm) (δ relative to residual solvent peak for ^1^H and ^13^C). Elemental analysis was performed on an EA3000 elemental analyzer. The 1,2,4-oxadiazolone derivatives were synthesized following the protocol described by Sangshetti et al. (2009) [50]. The synthesis for 1,3,4-oxadiazolone derivatives was partially based on the work by Jansen et al. (2007) [36]. All synthetic methodologies and structural determination results are in the Supplementary Materials.

### 4.2. Clot Lysis Assay

Fresh plasma was extracted from healthy donors after signing an informed consent form. Blood was collected into citrate-containing tubes (BD Vacutainer^®^ 9NC, 367691) to prevent early coagulation. Immediately after collecting it, blood was centrifuged at 1000× *g* for 15 min. Plasma was then extracted from the upper layer of each centrifuge tube, collected, and frozen at −20 °C. Plasma aliquots were used within the timespan of three weeks. Thromborel^®^ S (Siemens, OUHP20) was used as source of tissue factor. Prior to its use, the content of each vial was dissolved into 4 mL of ddH_2_O followed by vigorous shaking for two minutes. This solution was stored at 4 °C for up to three days. Recombinant human tissue plasminogen activator (abcam, ab92637) was used as a source for tPA. The stock concentration was 1.37 mg/mL, and it was stored at −20 °C and thawed right before its use. Tris hydrochloride, 1M, pH = 7.5 solution (Fisher BioReagents, 10573145) was used as a buffer. A 100 mM solution of CaCl_2_ was prepared by dissolving 0.111 g of anhydrous CaCl_2_ into 10 mL of ddH_2_O. The solution was then homogenized and stored at room temperature until further use.

The antifibrinolytic activity of all compounds was evaluated using clot lysis in vitro assays in human plasma. The assays were performed in flat-bottom 96-well plates. Plasma clots were pre-formed by mixing 140 µL of plasma, 20 µL of tissue factor, and 20 µL of a 100 mM CaCl_2_ solution. This mix was incubated at 37 °C for 30 min to achieve complete coagulation. A solution of tPA and the studied compound was previously prepared in Tris HCl buffer, and 20 µL was added on top of each plasma clot. The final concentration of tPA for all tests was 10.0 µg/mL (considering the total volume of 200 µL). The density of the clot was then assessed by measuring the absorbance at a wavelength of 405 nm every 1 min at a constant temperature of 37 °C. The assay was conducted for a maximum of 6 h, or until the clot was fully lysed.

The lysis rate was calculated from the absorbance slope for each assay, calculated from the initial and final values of absorbance and their respective time points. The final value was taken as the value at 6 h, or the value at which the clot was fully lysed.

(1) $$Absorbance\;slope=\frac{\left|{\mathrm{Abs}}_{final}-{\mathrm{Abs}}_{initial}\right|}{t_{final}-t_{initial}}$$

The fibrinolysis rate was calculated dividing this value by the same slope for the assay with no compound (with presence of tPA):

(2) $$Fibrinolysis\;rate\;\left(\%\right)=\frac{Slope_{Compound}}{Slope_{tPA}}\times 100$$

IC_50_ values for each compound were considered as the concentration that caused a 50% decrease in fibrinolytic activity, and therefore presented a 50% inhibitory effect on fibrinolysis.

### 4.3. Docking Studies

The 3D structures of the enzyme–ligand complexes were modelled with AutoDock 4.2. The protein structure of the Kringle 1 domain of plasminogen was taken from the Protein Data Bank with PDB accession code 1CEA. Ligand structures for EACA and TXA were extracted from protein structures with PDB codes 1CEA (EACA) and 1CEB (TXA). The structures for all ligands were generated with AVOGADRO. Both protein and ligand structures were first parametrized with AutoDockTools: polar hydrogens were added, AutoDock 4.2 atom typing was used, and Gasteiger partial charges were computed. All rotatable bonds of the ligands were considered free during the docking calculations, whereas the whole protein structure was kept fixed.

Dockings for all ligands were performed with AutoDock 4.2. A grid box of 38.25 × 37.5 × 33.75 Å with a grid point spacing of 0.375 Å, centered at the point (33.96, −1.477, −47.335), was used as the search space for docking. For each ligand docking, 1000 rounds of the genetic algorithm implemented in AutoDock 4.2 were performed. For each round, an initial population of 150 members was considered, with randomized initial positions and orientation coordinates and randomized conformations of the substrate flexible bonds. The genetic algorithm was extended up to 27,000 offspring generations with a maximum of 2,500,000 energy evaluations. Only low-energy and repetitive binding poses were considered for analysis. Distances were measured, and pictures of the complexes were generated using VMD [51].

### 4.4. Molecular Dynamics Studies

The protein was inserted into a cubic box of water molecules, ensuring that the solvent shell would extend for at least 0.8 nm around the system. Three sodium counterions were added. The GROMOS 54a7 force field was used for both the protein and ligands. The water molecules were described by the SPC/E model. Parameters for the ligands were generated with the PRODRG webserver [52]. The system was minimized, imposing harmonic position restraints of 1000 kJ·mol^−1^·nm^−2^ on solute atoms, allowing the equilibration of the solvent without distorting the solute structure. After an energy minimization of the solvent and the solute without harmonic restraints, the temperature was gradually increased from 0 to 298 K. This was performed by increasing the temperature from 0 to 298 K in 12 steps, in which the temperature was increased by 25 K in 100 ps of MD.

Constant temperature−pressure (T = 298 K, P = 1 bar) 20-ns dynamics were then performed through the Nosé–Hoover and Andersen–Parrinello–Rahman coupling schemes. Periodic boundary conditions were applied. The final simulation box equilibrated at around 5.61 × 5.61 × 5.61 nm. Long-range electrostatic interactions were treated with the particle mesh Ewald (PME) method using a grid with a spacing of 0.12 nm combined with a fourth-order B-spline interpolation to compute the potential and forces in between grid points. The cutoff radius for the Lenard–Jones interactions as well as for the real part of the PME calculations was set to 0.9.

The simulations were performed under a constant temperature−pressure scheme for 1000 ns for each compound with a time step of 2 fs. Analysis of the simulated trajectories were performed with VMD [51].

### 4.5. Molecular Surface Determination

Molecular surfaces were computed as described in the study by Varshney et al. [53]. A probe radius of 1.4 Å was used to measure the solvent accessible surface area. A total of 100 different conformations of each ligand were evaluated. Molecular surface values corresponded to the average of this ensemble.

## 5. Conclusions

This work presented the discovery of a new family of antifibrinolytic drugs. These compounds consisted of a terminal amine group, a triazole, and an oxadiazolone ring. Different variations were synthesized, analyzed in vitro, and studied computationally. The results showed that the optimal candidate tested (compound **1**) provided a 2.5-fold improvement in activity over TXA, the current gold standard, and a very low risk of interacting with the GABA_A_ receptor. The presence of 1,2,3-triazole in the molecule proved to be key, and the computational analysis revealed crucial interactions with Tyr72 to stabilize the protein–ligand complex. Furthermore, this constituted the first report of the antifibrinolytic activity of 1,2,3-triazole derivatives. In conclusion, this discovery opens the possibility to further investigate these molecules and/or other variations with the goal of finding better antifibrinolytic drugs.

## Figures and Tables

**Table 1:** IC_50_ values for all tested compounds. IC_50_ was considered as the concentration at which each inhibitor reduced the fibrinolytic activity by 50%.

Compound	IC_50_ (µM)	Compound	IC_50_ (µM)
TXA	33.3	6	>1000
EACA	263	7	335
**1**	13.8	8	27.8
**2**	138	9	>1000
**3**	>1000	10	>1000
**4**	>1000	11	60.4
**5**	36.8		

**Table 2:** Molecular surfaces calculated for GABA, TXA, 4-PIOL, and compound **1**.

Ligand	Molecular Surface (Å^2^)
GABA	256.4
TXA	293.7
4-PIOL	310.9
**1**	374.4

## Data Availability

Not applicable.

## Supplementary Materials

The supporting information can be downloaded at: https://www.mdpi.com/article/10.3390/ijms232314942/s1.

## Abbreviations

ACN, acetonitrile; CDI, carbonyl diimidazole; DBU; 1,8-Diazabicyclo(5.4.0)undec-7-ene; DCM, dichloromethane; DMF; dimethyl formamide, DMSO, dimethyl sulfoxide; EACA, epsilon-aminocaproic acid; LBS, lysine binding site; PLG, plasminogen; TEA, triethylamine; TFAA, trifluoroacetic acid anhydride; THF, tetrahydrofuran; tPa, tissue plasminogen activator; TXA, tranexamic acid; uPa, urokinase plasminogen activator.
