# Supplementary material for: 1,2,3-Triazole Derivatives as Novel Antifibrinolytic Drugs
Source: Int J Mol Sci. 2022 Nov 29;23(23):14942. doi: 10.3390/ijms232314942 (PMC9736318; doi:10.3390/ijms232314942)
Supplement: Supplementary file 1 [file ijms-23-14942-s001.zip › ijms-2019216-supplementary.pdf]

1,2,3-triazole derivatives as novel antifibrinolytic drugs

*Oriol Bosch-Sanz [1,4], Yvette Rabadà [1], Xevi Biarnés [2], Javier Pedreño [4,5], Luis Caved [5], Mercedes Balcells [1,4†], Jordi Martorell [1†], David Sánchez-García [3]**

[1] IQS School of Engineering, Universitat Ramon Llull. Via Augusta 390, 08017 Barcelona, Spain.

[2] Laboratory of Biochemistry, Universitat Ramon Llull. Via Augusta 390, 08017 Barcelona, Spain.

[3] Grup d'Enginyeria de Materials, Universitat Ramon Llull. Via Augusta 390, 08017 Barcelona, Spain.

[4] Massachusetts Institute of Technology. 77 Massachusetts Ave, Cambridge, MA 02139, USA.

[5] Alxerion Biotech. 245 First St, Riverview II, 18th Floor, Cambridge, MA 02142, USA.

†Senior Author.

*Corresponding Author.

Table of Contents

I. Synthetic Methodologies 2
II. NMR Spectroscopy 44
III. Docking with $GABA_A$ receptor 64

I. Synthetic Methodologies

tert-butyl 4-((methylsulfonyl)oxy)piperidine-1-carboxylate (13)

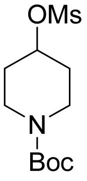

13

To a stirred solution of *tert*-butyl 4-hydroxypiperidine-1-carboxylate (**12**) (0.96 g, 4.8 mmol) in 10 mL dichloromethane was added triethylamine (1 mL) and stirred at room temperature for 5 minutes and then cooled to 0-5 °C. Methanesulfonyl chloride (0.67 g, 6.2 mmol) was added dropwise at 0-5 °C and after addition the reaction mixture was allowed to warm to room temperature and then stirred at room temperature for 2 h. After completion (monitored by TLC), the reaction was quenched by addition of distilled water, additional dichloromethane was added and the dichloromethane layer after extraction was separated, dried ($MgSO_4$) and concentrated *in vacuo* to yield 1.3 g of *tert*-butyl 4-((methylsulfonyl)oxy)piperidine-1-carboxylate (**13**) as a pale yellow solid. Yield: 97 %. ^{1}H-NMR (400 MHz, $CDCl_3$) δ (ppm): 4.84 – 4.90 (m, 1H), 3.66 – 3.72 (m, 2H), 3.25 – 3.32 (m, 2H), 3.02 (s, 3H), 1.91 – 1.99 (m, 2H), 1.76 – 1.84 (m, 2H), 1.44 (s, 9H).

tert-butyl 4-azidopiperidine-1-carboxylate (14)

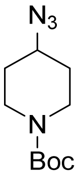

14

To a stirred solution of *tert*-butyl 4-((methylsulfonyl)oxy)piperidine-1-carboxylate (**13**) (2.48 g, 8.9 mmol) in 20 mL dimethyl formamide, sodium azide (1.74, 26.8 mmol) was added and the resulting suspension was stirred at 80 °C for 8 h. After completion (monitored by TLC), the

reaction was allowed to cool to room temperature and then poured into cold water with occasional stirring. The residue was extracted with of ethyl acetate (3x10 mL). The combined organic layers were concentrated *in vacuo* and the residue was dissolved in hexane (50 mL) and an extraction with water (3x10 mL) was made. The combined organic layers were dried ($MgSO_4$) and concentrated *in vacuo* to yield 1.75 g of *tert*-butyl 4-azidopiperidine-1-carboxylate (**14**) as a yellow oil. Yield: 87%. ^{1}H-NMR (400 MHz, $CDCl_3$) δ (ppm): 3.81 – 3.76 (m, 2H), 3.51 – 3.56 (m, 1H), 3.02 – 3.09 (m, 2H), 1.79 – 1.86 (m, 2H), 1.49 – 1.56 (m, 2H), 1.44 (s, 9H).

***tert*-butyl 4-(4-(ethoxycarbonyl)-1*H*-1,2,3-triazol-1-yl)piperidine-1-carboxylate (15)**

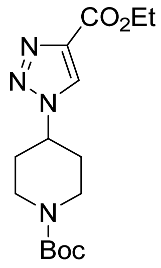

15

tert-butyl 4-azidopiperidine-1-carboxylate (**14**) (0.66 g, 2.9 mmol) was taken in acetonitrile (15 mL) and to it ethyl propiolate (0.29 g, 2.9 mmol) was added, followed by CuI (0.11 g, 0.6 mmol) and the resulting solution was stirred at room temperature for 12 h. After completion (monitored by TLC), solvent was removed *in vacuo* and distilled water was added to the residue and then extracted with 3 portions (3x10 mL) of ethyl acetate. The combined organic layers were dried ($MgSO_4$) and concentrated *in vacuo* to yield 0.79 g of the desired compound *tert*-butyl 4-(4-(ethoxycarbonyl)-1*H*-1,2,3-triazol-1-yl)piperidine-1-carboxylate (**15**) as a pale yellow solid. Yield: 84%. ^{1}H-NMR (400 MHz, $CDCl_3$) δ (ppm): 8.08 (s, 1H), 4.62 – 4.70 (m, 1H), 4.40 (q, *J*=7.1Hz, 2H), 4.23 – 4.31 (m, 2H), 2.89 – 2.93 (m, 2H), 2.18 – 2.23 (m, 2H), 1.88 – 1.98 (m, 2H), 1.46 (s, 9H), 1.39 (t, *J*=7.1Hz, 3H).

***tert*-butyl 4-(4-(hydrazinecarbonyl)-1*H*-1,2,3-triazol-1-yl)piperidine-1-carboxylate (29)**

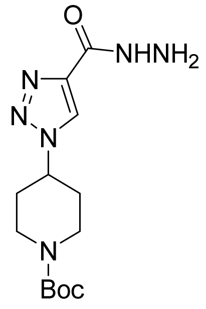

29

tert-butyl 4-(4-(ethoxycarbonyl)-1*H*-1,2,3-triazol-1-yl)piperidine-1-carboxylate (**15**) (1 g, 3.1 mmol) and hydrazine hydrate (0.5 g) in 20 mL of *n*-butanol were refluxed for 3 h. Then, the solvent was removed by evaporation under vacuum. The residue was treated with dichloromethane and washed with water. The organic phase was dried ($MgSO_4$) and the solvent removed under reduced pressure. The resulting pale-yellow solid was washed with cold ethanol. Yield 0.93 g, 97%. ^{1}H NMR (400 MHz, $CDCl_3$), δ (ppm): 8.08 (s, 1H), 4.63 (tt, *J* = 11.6, 3.6 Hz, 1H), 4.27 (s, 2H), 4.03 (s, 1H), 2.94 (t, *J* = 13.0 Hz, 2H), 2.21 (d, *J* = 12.8 Hz, 2H), 1.94 (qd, *J* = 12.0, 4.4 Hz, 2H), 1.47 (d, *J* = 0.6 Hz, 9H).

***tert*-butyl 4-(4-(5-oxo-4,5-dihydro-1,3,4-oxadiazol-2-yl)-1*H*-1,2,3-triazol-1-yl)piperidine-1-carboxylate (32)**

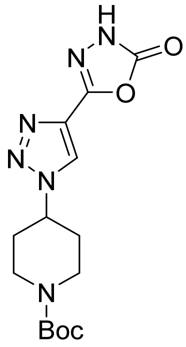

32

To a suspension *tert*-butyl 4-(4-(hydrazinecarbonyl)-1*H*-1,2,3-triazol-1-yl)piperidine-1-carboxylate (**29**) (0.15 g, 0.50 mmol) in anhydrous acetonitrile (5 mL) *N,N'*-carbonyldiimidazole (CDI) (0.10 g, 0.65 mmol) and DBU (0.1 mL) were added at room temperature. The mixture was stirred for 15 h under reflux. After completion (monitored by TLC), 10% (v/v) acetic acid (25 mL) was added, and the mixture was extracted with ethyl acetate (3x10 mL). The combined organic layers washed with distilled water (3x10 mL), dried ($MgSO_4$) and concentrated *in vacuo*. to provide 0.15 g of compound **32** as a white solid. Yield: 89%. ^{1}H NMR (400 MHz, $CDCl_3$), δ (ppm): 9.52 (s, 1H), 8.05 (s, 1H), 4.70 (tt, *J* = 11.7, 4.1 Hz, 1H), 4.29 (d, *J* = 11.9 Hz, 2H), 2.96 (t, *J* = 12.7 Hz, 2H), 2.38 – 2.17 (m, 2H), 2.17 – 1.88 (m, 2H), 1.47 (s, 9H). ^{13}C NMR (100 MHz, d_6-DMSO), δ (ppm): 154.5, 154.1, 148.7, 133.6, 124.1, 79.4, 58.2, 42.2, 32.0, 28.50. IR (cm^{-1}): 3130.52, 2986.99, 1747.35, 1704.20, 1540.88, 1410.72, 1366.35, 1235.55, 1149.52, 999.32, 950.23, 728.20. HRMS (ESI-FIA-TOF): m/z calculated for $C_{14}H_{20}N_6NaO_4$ 359.1444, found 359.1438.

5-(1-(piperidin-4-yl)-1*H*-1,2,3-triazol-4-yl)-1,3,4-oxadiazol-2(3*H*)-one hydrochloride (5)

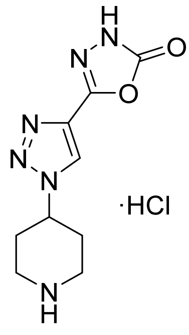

5

A mixture of *tert*-butyl 4-(4-(5-oxo-4,5-dihydro-1,3,4-oxadiazol-2-yl)-1*H*-1,2,3-triazol-1-yl)piperidine-1-carboxylate (**32**) (100 mg, 0.3 mmol) and 4N HCl in dioxane (2 mL) was stirred at room temperature for 2 h. The solvent was removed *in vacuo* and the resulting yellow solid was triturated with EtOAc to provide 74 mg of the hydrochloride of 5-(1-(piperidin-4-yl)-1*H*-1,2,3-

triazol-4-yl)-1,3,4-oxadiazol-2(3*H*)-one as a white solid. Yield: 91%. ^{1}H NMR (400 MHz, d_6-DMSO), δ (ppm): 13.24 (s, 1H), 9.26 (s, 1H), 9.10 (s, 1H), 8.24 (s, 1H), 5.28 – 5.02 (m, 1H), 3.38 (d, *J* = 12.7 Hz, 2H), 3.05 (q, *J* = 11.7 Hz, 2H), 2.40 – 1.99 (m, 4H). ^{13}C NMR (100 MHz, d_6-DMSO), δ (ppm): 154.0, 148.1, 133.1, 124.2, 54.9, 41.7, 28.2. Elemental analysis calculated (%) for $C_9H_{13}ClN_6O_2$: C 39.64, H 4.81, N 30.82. Found C 39.47, H 4.56, N 30.77. IR (cm^{-1}): 2939.70, 2802.84, 1808.57, 1770.15, 1657.33, 1397.35, 1397.35, 1213.28, 1048.40, 892.53. HRMS (ESI-FIA-TOF): m/z calculated for $C_9H_{13}N_6O_2$ 237.1095, found 237.1093.

***tert*-butyl 4-(4-carbamoyl-1*H*-1,2,3-triazol-1-yl)piperidine-1-carboxylate (35)**

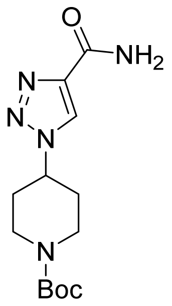

35

To a solution of *tert*-butyl 4-(4-(ethoxycarbonyl)-1*H*-1,2,3-triazol-1-yl)piperidine-1-carboxylate (**15**) (2.04 g, 6.3 mmol) in 20 mL of methanol, 40 mL of ammonia in methanolic solution was added and the resulting solution was stirred at room temperature for 12 h. After completion (monitored by TLC), solvent was removed *in vacuo* and distilled water was added to the residue. It was extracted with 3 portions (3x10 mL) of dichloromethane. The combined organic layers were dried ($MgSO_4$) and concentrated *in vacuo* to yield 1.69 g of *tert*-butyl 4-(4-carbamoyl-1*H*-1,2,3-triazol-1-yl)piperidine-1-carboxylate (**35**) as a white solid. Yield: 91 %. ^{1}H-NMR (400 MHz, $CDCl_3$) δ (ppm): 8.10 (s, 1H), 7.00 (s, 1H), 5.70 (s, 1H), 4.62 – 4.70 (m, 1H), 4.24 – 4.31 (m, 2H), 2.89 – 3.01 (m, 2H), 2.19 – 2.23 (m, 2H), 1.88 – 1.99 (m, 2H), 1.44 (s, 9H).

tert-butyl 4-(4-cyano-1H-1,2,3-triazol-1-yl)piperidine-1-carboxylate (39)

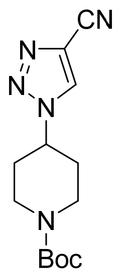

39

A solution of *tert*-butyl 4-(4-carbamoyl-1*H*-1,2,3-triazol-1-yl)piperidine-1-carboxylate (**35**) (0.21 g, 0.7 mmol) and 0.4 mL of triethylamine in 9 mL of dichloromethane was stirred at room temperature. The resulting solution was then cooled to 0-5 °C in an ice bath. To this cooled solution, trifluoroacetic anhydride (0.30 g, 1.5 mmol) was added. The resulting solution was stirred for 5 h. After completion (monitored by TLC), solvent was removed *in vacuo* and distilled water added to the residue and then extracted with 3 portions (3x10mL) of dichloromethane. The organic layer was washed with saturated $NaHCO_3$ solution, dried ($MgSO_4$) and concentrated *in vacuo* to yield 0.19 g of *tert*-butyl 4-(4-cyano-1*H*-1,2,3-triazol-1-yl)piperidine-1-carboxylate (**39**) as a brown oil. Yield: 97%. ^{1}H-NMR (400 MHz, $CDCl_3$) δ (ppm): 8.12 (s, 1H), 4.61 – 4.72 (m, 1H), 4.25 – 4.30 (m, 2H), 2.89 – 3.03 (m, 2H), 2.20 – 2.26 (m, 2H), 1.92 – 2.04 (m, 2H), 1.48 (s, 9H).

tert-butyl (Z)-4-(4-(N'-hydroxycarbamimidoyl)-1H-1,2,3-triazol-1-yl)piperidine-1-carboxylate (43)

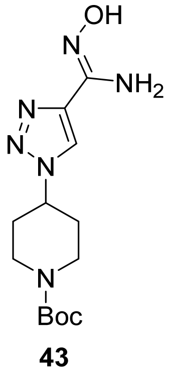

43

To a stirred solution of *tert*-butyl 4-(4-cyano-1*H*-1,2,3-triazol-1-yl)piperidine-1-carboxylate (**39**) (0.14 g, 0.5 mmol) in 10 mL methanol, hydroxylamine hydrochloride (0.07g, 1 mmol) and sodium bicarbonate (0.07 g, 0.9 mmol) was added. The resulting mixture was heated under reflux for 14 h. After completion (monitored by TLC), solvent was removed *in vacuo* and distilled water added to the residue and then extracted with 3 portions (3x10mL) of dichloromethane. The organic layer dried ($MgSO_4$) and concentrated *in vacuo*. A small portion of ethyl acetate was added and stirred at room temperature for 30 minutes. The resulting solid, *tert*-butyl (*Z*)-4-(4-(*N*'-hydroxycarbamimidoyl)-1*H*-1,2,3-triazol-1-yl)piperidine-1-carboxylate (**43**) was collected by filtration. After drying under vacuum, 0.04 g of **43** were obtained. Yield: 23%. ^{1}H-NMR (400 MHz, d_6-DMSO) δ (ppm): 9.50 (s, 1H), 8.35 (s, 1H), 5.71 (s, 2H), 4.69 – 4.77 (m, 1H), 4.03 – 4.07 (m, 2H), 2.95 (s, 2H), 2.05 – 2.09 (m, 2H), 1.82 – 1.93 (m, 2H), 1.42 (s, 9H).

***tert*-butyl 4-(4-(5-oxo-4,5-dihydro-1,2,4-oxadiazol-3-yl)-1*H*-1,2,3-triazol-1-yl)piperidine-1-carboxylate (47)**

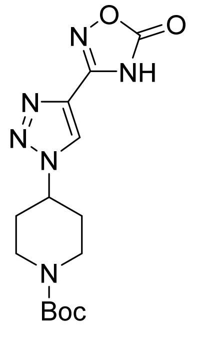

47

To a suspension *tert*-butyl (*Z*)-4-(4-(*N*'-hydroxycarbamimidoyl)-1*H*-1,2,3-triazol-1-yl)piperidine-1-carboxylate (**43**) (0.15 g, 0.50 mmol) in anhydrous acetonitrile (5 mL) *N,N'*-carbonyldiimidazole (CDI) (0.10 g, 0.65 mmol) and DBU (0.1 mL) were added at room temperature. The mixture was stirred for 15 h under reflux. After completion of the reaction (monitored by TLC), 10% (v/v) acetic acid (25 mL) was added, and the mixture was extracted

with ethyl acetate (3x10 mL). The combined organic layers washed with distilled water (3x10 mL), dried ($MgSO_4$) and concentrated *in vacuo* to furnish 96 mg of compound **47**. Yield: 57%. ^{1}H NMR (400 MHz, d_6-DMSO), δ (ppm): 8.57 (s, 1H), 6.92 (s, 1H), 4.71 – 4.77 (m, 1H), 4.00 – 4.03 (m, 2H), 2.82 – 3.01 (m, 2H), 2.03 – 2.08 (m, 2H), 1.81 – 1.89 (m, 2H), 1.38 (s, 9H). ^{13}C NMR (100 MHz, d_6-DMSO), δ (ppm): 159.6, 154.2, 151.9, 150.6, 139.8, 79.4, 57.5, 42.9, 31.6, 28.4. IR (cm^{-1}): 3301.13, 3127.81, 2973.45, 2848.88, 1788.44, 1679.12, 1530.05, 1400.15, 1365.24, 1243.75, 1166.05, 1052.23, 973.32, 930.96, 763.27. HRMS (ESI-FIA-TOF): m/z calculated for $C_{14}H_{21}N_6O_4$ 337.1624, found 337.1619.

hydrochloride of 3-(1-(piperidin-4-yl)-1*H*-1,2,3-triazol-4-yl)-1,2,4-oxadiazol-5(4*H*)-one (1)

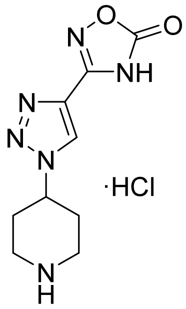

1

A mixture of *tert*-butyl 4-(4-(5-oxo-4,5-dihydro-1,2,4-oxadiazol-3-yl)-1*H*-1,2,3-triazol-1-yl)piperidine-1-carboxylate (**47**) (10 mg, 0.03 mmol) and 4N HCl in dioxane (2 mL) was stirred at 23 °C for 2 h. The solvent was removed *in vacuo* and the resulting yellow solid was triturated with EtOAc to provide 7 mg of the hydrochloride of 3-(1-(piperidin-4-yl)-1*H*-1,2,3-triazol-4-yl)-1,2,4-oxadiazol-5(4*H*)-one (**1**) as a white solid. Yield: 90%. ^{1}H NMR (400 MHz, d_6-DMSO), δ (ppm): 13.28 (s, 1H), 9.10 (s, 1H), 8.90 (s, 1H), 8.89 (s, 1H), 4.92 (m, 1H), 3.32 (m, 2H), 3.09 (m, 2H), 2.33 – 2.24 (m, 4H). ^{13}C NMR (100 MHz, d_6-DMSO), δ (ppm): 159.4, 151.5, 131.9, 124.4, 55.1, 41.7, 30.6, 28.3. Elemental analysis calculated (%) for $C_9H_{13}ClN_6O_2$: C 39.64, H 4.81, N 30.82. Found C 39.89, H 4.72, N 30.97. IR (cm^{-1}): 3030.32, 2824.51, 2518.49, 1765.15, 1610.26,

1560.46, 1436.31, 1213.61, 1056.13, 923.46, 888.00, 836.19, 760.76. HRMS (ESI-FIA-TOF): m/z calculated for $C_9H_{13}N_6O_2$ 237.1095, found 237.1092.

***tert*-butyl (2-azidoethyl)carbamate (19)**

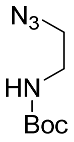

19

tert-butyl (2-bromoethyl)carbamate (**16**) (0.94 g, 4.2 mmol) and sodium azide (0.7 mg, 8.4 mmol) were dissolved in DMF (10 mL). The reaction mixture was refluxed overnight at 75 ºC with stirring. Subsequently, the solvent was evaporated and the reaction mixture was diluted with dichloromethane (50 mL) and washed with water (3x50 mL). Organic layer was collected, and solvent was evaporated, affording 0.74 g the compound **19** as pale-yellow oil. Yield: 94%. ^{1}H-NMR (400 MHz, $CDCl_3$) δ (ppm): 4.75 - 5.00 (s, 1H), 3.38 (t, J = 5.6 Hz, 2H), 3.28 (q, J = 5.8 Hz, 2H), 1.42 (s, 9H).

***tert*-butyl (3-azidopropyl)carbamate (20)**

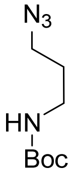

20

tert-butyl (3-bromopropyl)carbamate (**17**) (1.0 g, 4.2 mmol) and sodium azide (0.7 mg, 8.4 mmol) were dissolved in DMF (10 mL). The reaction mixture was refluxed overnight at 75 ºC with stirring. Subsequently, the solvent was evaporated and the reaction mixture was diluted with dichloromethane (50 mL) and washed with water (3x50 mL). Organic layer was collected, and solvent was evaporated, affording 0.80 g of compound **20** as pale-yellow oil. Yield: 95%. ^{1}H-NMR

(400 MHz, $CDCl_3$) δ (ppm): 4.45 - 4.90 (s, 1H), 3.32 (t, J = 6.7 Hz, 2H), 3.17 (t, J = 6.7 Hz, 2H), 1.74 (m, J = 6.7 Hz, 2H), 1.41 (s, 9H).

***tert*-butyl (4-azidobutyl)carbamate (21)**

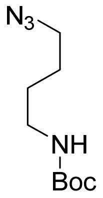

21

tert-butyl (4-bromobutyl)carbamate (**18**) (1.1 g, 4.2 mmol) and sodium azide (0.7 mg, 8.4 mmol) were dissolved in DMF (10 mL). The reaction mixture was refluxed overnight at 75 ºC with stirring. Subsequently, the solvent was evaporated and the reaction mixture was diluted with dichloromethane (50 mL) and washed with water (3x50 mL). Organic layer was collected, and solvent was evaporated, affording 0.86 g the product as pale-yellow oil. Yield: 96%. ^{1}H-NMR (400 MHz, $CDCl_3$) δ (ppm): 4.50 – 4.70 (s, 1H), 3.27 (t, J = 6.5 Hz, 2H), 3.10 (q, J = 6.5 Hz, 2H), 1.48-1.63 (m, 4H), 1.41 (s, 9H).

ethyl 1-(2-((*tert*-butoxycarbonyl)amino)ethyl)-1*H*-1,2,3-triazole-4-carboxylate (22)

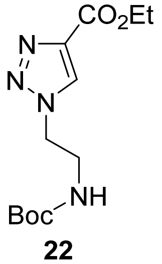

22

tert-butyl (2-azidoethyl)carbamate (**19**) (0.56 g, 3.0 mmol) was taken in acetonitrile (15 mL) and to it ethyl propiolate (0.33 g, 3.3 mmol) was added, followed by CuI (0.11 g, 0.6 mmol) and the resulting solution was stirred at room temperature for 15 h. After completion (monitored by TLC), solvent was removed *in vacuo* and distilled water was added to the residue and then extracted with

3 portions (3x10 mL) of ethyl acetate. The combined organic layers were dried ($MgSO_4$) and concentrated *in vacuo* to obtain 0.74 g the desired compound as a pale-yellow solid. Yield: 87%. ^{1}H-NMR (400 MHz, d_6-DMSO) δ (ppm): 8.66 (s, 1H), 6.97 (t, *J* = 5.9 Hz, 1H), 4.45 (t, 2H), 4.31 (q, *J* = 7.1 Hz, 2H), 3.40 (q, *J* = 6.4 Hz, 2H), 1.32 (s, 9H), 1.29 (t, *J* = 7.1 Hz, 3H).

ethyl 1-(3-((*tert*-butoxycarbonyl)amino)propyl)-1*H*-1,2,3-triazole-4-carboxylate (23)

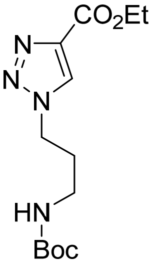

23

tert-butyl (3-azidopropyl)carbamate (**20**) (0.60 g, 3.0 mmol) was taken in acetonitrile (15 mL) and to it ethyl propiolate (0.33 g, 3.3 mmol) was added, followed by CuI (0.11 g, 0.6 mmol) and the resulting solution was stirred at room temperature for 15 h. After completion (monitored by TLC), solvent was removed *in vacuo* and distilled water was added to the residue and then extracted with 3 portions (3x10 mL) of ethyl acetate. The combined organic layers were dried ($MgSO_4$) and concentrated *in vacuo* to yield 0.79 g of the desired compound as a pale-yellow solid. Yield: 84%. ^{1}H-NMR (400 MHz, $CDCl_3$) δ (ppm): 8.31 (s, 1H), 4.78 (s, 1H), 4.49 (t, *J* = 6.5 Hz, 2H), 4.42 (q, *J* = 6.8 Hz, 2H), 3.12 (q, *J* = 6.1 Hz 2H), 2.09 (m, *J* = 6.5 Hz, 2H), 1.40 (s, 9H), 1.36 (t, 3H).

ethyl 1-(4-((*tert*-butoxycarbonyl)amino)butyl)-1*H*-1,2,3-triazole-4-carboxylate (24)

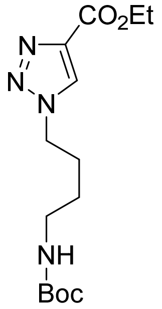

24

tert-butyl (4-azidobutyl)carbamate (**21**) (0.64 g, 3.0 mmol) was taken in acetonitrile (15 mL) and to it ethyl propiolate (0.33 g, 3.3 mmol) was added, followed by CuI (0.11 g, 0.6 mmol) and the resulting solution was stirred at room temperature for 15 h. After completion (monitored by TLC), solvent was removed *in vacuo* and distilled water was added to the residue and then extracted with 3 portions (3x10 mL) of ethyl acetate. The combined organic layers were dried ($MgSO_4$) and concentrated *in vacuo* to obtain 0.81 g the desired compound as a pale-yellow solid. Yield: 87%. ^{1}H-NMR (400 MHz, $CDCl_3$) δ (ppm): 8.08 (s, 1H) 4.62 (s, 1H), 4.43 (t, 2H), 4.38 (q, 2H), 3.13 (q, J = 6.7 Hz, 2H), 1.94 (m, 2H), 1.48 (m, 2H), 1.40 (s, 9H), 1.37 (t, J = 7.1 Hz, 3H).

***tert*-butyl (2-(4-(hydrazinecarbonyl)-1*H*-1,2,3-triazol-1-yl)ethyl)carbamate (30)**

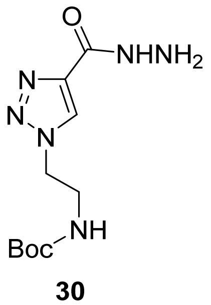

30

Ethyl 1-(2-((*tert*-butoxycarbonyl)amino)ethyl)-1*H*-1,2,3-triazole-4-carboxylate (**22**) (1.6 g, 5.7 mmol) and hydrazine hydrate (1.4 g, 28.7 mmol) in 15 mL of *n*-butanol were refluxed for 5 h. Then, the solvent was removed by evaporation under vacuum. The resulting solid was left drying

under vacuum in the presence of P_2O_5. 1.32 g of compound **30** were obtained. Yield: 86%. ^{1}H-NMR (400 MHz, *d*$_6$-DMSO) δ (ppm): 8.42 (s, 1H), 6.96 (t, *J* = 5.8 Hz, 1H), 4.39 (t, *J* = 5.9 Hz, 2H), 3.36 (q, *J* = 5.9 Hz, 2H), 1.31 (s, 9H).

***tert*-butyl (3-(4-(hydrazinecarbonyl)-1*H*-1,2,3-triazol-1-yl)propyl)carbamate (31)**

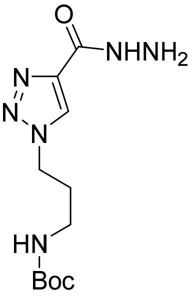

31

Ethyl 1-(3-((*tert*-butoxycarbonyl)amino)propyl)-1*H*-1,2,3-triazole-4-carboxylate (**23**) (1.7 g, 5.7 mmol) and hydrazine hydrate (1.4 g, 28.7 mmol) in 15 mL of *n*-butanol were refluxed for 5 h. Then, the solvent was removed by evaporation under vacuum. The resulting solid was left drying under vacuum in the presence of P_2O_5. 1.5 g of compound **31** were obtained. Yield: 94 %. ^{1}H-NMR (400 MHz, *d*$_6$-DMSO) δ (ppm): 8.52 (s, 1H), 6.92 (t, *J* = 5.7 Hz, 1H), 4.38 (t, *J* = 7.0 Hz, 2H), 2.90 (q, *J* = 6.5 Hz, 2H), 1.93 (m, *J* = 6.9 Hz, 2H), 1.35 (s, 9H).

***tert*-butyl (2-(4-(5-oxo-4,5-dihydro-1,3,4-oxadiazol-2-yl)-1*H*-1,2,3-triazol-1-yl)ethyl)carbamate (33)**

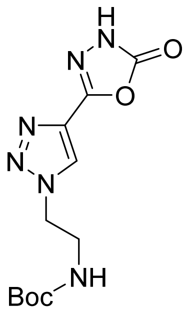

33

To a suspension of *tert*-butyl (3-(4-(hydrazinecarbonyl)-1*H*-1,2,3-triazol-1-yl)ethyl)carbamate (**30**) (0.50 mmol) in anhydrous acetonitrile (5 mL) *N,N'*-carbonyldiimidazole (CDI) (0.10 g, 0.65 mmol) and DBU (0.1 mL) were added at room temperature. The mixture was stirred for 4 h under reflux. After completion (monitored by TLC), 10% (v/v) acetic acid (25 mL) was added, and the mixture was extracted with ethyl acetate (3x10 mL). The combined organic layers were washed with distilled water (3x10 mL), dried ($MgSO_4$) and concentrated *in vacuo* to afford 0.11 g of compound **33**. Yield: 78%. ^{1}H-NMR (400 MHz, d_6-DMSO) δ (ppm): 12.59 (s, 1H), 8.70 (s, 1H), 6.97 (t, J = 5.9 Hz, 1H), 4.45 (t, J = 6.5 Hz, 2H), 3.39 (q, J = 5.9 Hz, 2H), 1.30 (s, 9H). ^{13}C-NMR (100 MHz, CD_3OD) δ (ppm): 156.8, 155.1, 148.7, 133.6, 124.9, 79.0, 50.2, 39.9, 27.2. IR (cm^{-1}): 3382.37, 3133.23, 2986.99, 1792.22, 1764.24, 1692.66, 1518.65, 1273.38, 1166.36, 1045.09, 861.15. HRMS (ESI-FIA-TOF): m/z calculated for $C_{11}H_{17}N_6O_4$ 297.1306, found 297.1306.

***tert*-butyl (3-(4-(5-oxo-4,5-dihydro-1,3,4-oxadiazol-2-yl)-1*H*-1,2,3-triazol-1-yl)propyl)carbamate 34)**

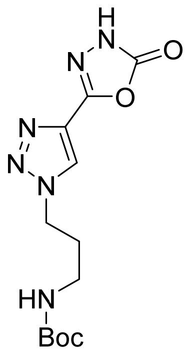

34

To a suspension of *tert*-butyl (3-(4-(hydrazinecarbonyl)-1*H*-1,2,3-triazol-1-yl)propyl)carbamate (**31**) (0.50 mmol) in anhydrous acetonitrile (5 mL) *N,N'*-carbonyldiimidazole (CDI) (0.10 g, 0.65 mmol) and DBU (0.1 mL) were added at room temperature. The mixture was stirred for 4 h under reflux. After completion (monitored by TLC), 10% (v/v) acetic acid (25 mL) was added, and the

mixture was extracted with ethyl acetate (3x10 mL). The combined organic layers washed with distilled water (3x10 mL), dried ($MgSO_4$) and concentrated *in vacuo* to afford 0.12 g of compound **34**. Yield: 82%. ^{1}H-NMR (400 MHz, *d6*-DMSO) δ (ppm): 12.57 (s, 1H), 8.76 (s, 1H), 6.92 (t, *J* = 5.7 Hz, 1H), 4.33 (t, *J* = 7.0 Hz, 2H), 2.92 (q, *J* = 6.5 Hz, 2H), 1.95 (m, *J* = 6.9 Hz, 2H), 1.35 (s, 9H). ^{13}C-NMR (100 MHz, *d6*-DMSO) δ (ppm): 156.0, 154.5, 148.7, 133.5, 125.8, 78.2, 48.2, 37.4, 30.4, 28.6. IR (cm^{-1}): 3341.75, 3127.81, 2986.99, 1766.73, 1690.71, 1518.53, 1363.91, 1239.24, 1172.27, 951.79, 722.10. HRMS (ESI-FIA-TOF): m/z calculated for $C_{12}H_{19}N_6O_4$ 311.1462, found 311.1459.

5-(1-(2-aminoethyl)-1*H*-1,2,3-triazol-4-yl)-1,3,4-oxadiazol-2(3*H*)-one hydrochloride (6)

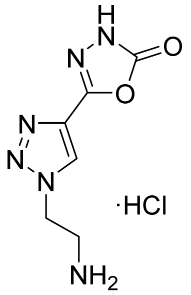

6

A mixture of *tert*-butyl (2-(4-(5-oxo-4,5-dihydro-1,3,4-oxadiazol-2-yl)-1*H*-1,2,3-triazol-1-yl)ethyl)carbamate (**33**) (100 mg, 0.3 mmol) and 3M HCl in methanol (5 mL) was stirred at room temperature for 2 h. The solvent was removed *in vacuo* and the resulting yellow solid was triturated with EtOAc to provide 60 mg of the corresponding hydrochloride as a white solid. Yield: 85%. ^{1}H-NMR (400 MHz, *d6*-DMSO) δ (ppm): 12.67 (s, 1H), 8.84 (s, 1H), 8.13 (s, 3H), 4.73 (t, *J* = 6.5 Hz, 2H), 3.38 (t, *J* = 6.0 Hz, 2H). ^{13}C-NMR (100.0 MHz, *d6*-DMSO) δ (ppm): 154.5, 148.6, 133.7, 126.5, 47.8, 36.7. IR (cm^{-1}): 3295.71, 3100.73, 2890.82, 1773.42, 1662.28, 1524.63, 1197.63, 1056.54, 950.37, 772.68, 698.62. HRMS (ESI-FIA-TOF): m/z calculated for $C_6H_9N_6O_2$ 197.0781, found 197.0779.

5-(1-(3-aminopropyl)-1*H*-1,2,3-triazol-4-yl)-1,3,4-oxadiazol-2(3*H*)-one hydrochloride (7)

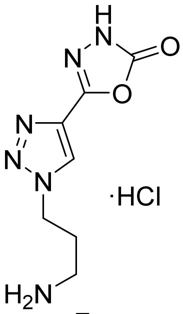

7

A mixture of *tert*-butyl (3-(4-(5-oxo-4,5-dihydro-1,3,4-oxadiazol-2-yl)-1*H*-1,2,3-triazol-1-yl)propyl)carbamate (**34**) (100 mg, 0.3 mmol) and 3M HCl in methanol (5 mL) was stirred at room temperature for 2 h. The solvent was removed *in vacuo* and the resulting yellow solid was triturated with EtOAc to provide 67 mg of the corresponding hydrochloride as a white solid. Yield: 81%. ^{1}H-NMR (400 MHz, d_6-DMSO) δ (ppm): 12.66 (s, 1H), 8.83 (s, 1H), 7.82 – 8.05 (s, 3H), 4.56 (t, J = 6.8 Hz, 2H), 2.79 (t, J = 7.6 Hz, 2H), 2.15 (m, 2H). ^{13}C-NMR (100 MHz, d_6-DMSO) δ (ppm): 154.5, 148.6, 133.6, 126.0, 47.6, 36.6, 28.0. IR (cm^{-1}): 3206.34, 3041.15, 2935.54, 1774.45, 1502.96, 1213.20, 946.02, 908.68, 756.25, 721.25. HRMS (ESI-FIA-TOF): m/z calculated for $C_7H_{11}N_6O_2$ 211.0938, found 211.0933.

***tert*-butyl (3-(4-carbamoyl-1*H*-1,2,3-triazol-1-yl)propyl)carbamate (36)**

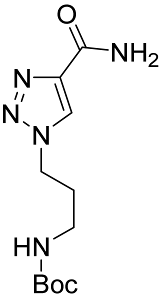

36

To a solution of ethyl 1-(3-((*tert*-butoxycarbonyl)amino)propyl)-1*H*-1,2,3-triazole-4-carboxylate (**23**) (2.48 g, 8.3 mmol) in 20 mL of ethanol, 30 mL of aqueous ammonia were added,

and the resulting solution was stirred at room temperature for 12 h. After completion (monitored by TLC), distilled water was added, and the mixture was filtrated under vacuum. The resulting white solid was washed with distilled water (3x10 mL) and dried under vacuum and in the presence of P_2O_5 overnight. 1.45 g of compound **36** were obtained. Yield: 65 %. ^{1}H-NMR (400 MHz, $CDCl_3$) δ (ppm): 8.32 (s, 1H), 7.07 (s, 1H), 5.90 (s, 1H), 5.07 (s, 1H), 4.48 (t, J = 6.8 Hz, 2H), 3.14 (q, J = 7.2 Hz, 2H), 2.18-2.07 (m, 2H), 1.44 (s, 9H). ^{13}C-NMR (100.0 MHz, d_6-DMSO) δ (ppm): 162.0, 156.1, 143.3, 127.1, 78.2, 47.9, 37.4, 30.5, 28.7.

***tert*-butyl (4-(4-carbamoyl-1*H*-1,2,3-triazol-1-yl)butyl)carbamate (37)**

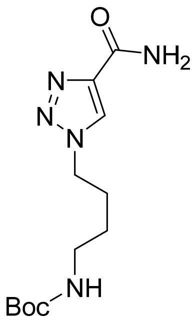

37

To a solution of ethyl 1-(4-((*tert*-butoxycarbonyl)amino)butyl)-1*H*-1,2,3-triazole-4-carboxylate (**24**) (2.48 g, 8.3 mmol) in 20 mL of ethanol, 30 mL of aqueous ammonia were added, and the resulting solution was stirred at room temperature for 12 h. After completion (monitored by TLC), distilled water was added, and the mixture was filtrated under vacuum. The resulting white solid was washed with distilled water (3x10 mL) and dried under vacuum and in the presence of P_2O_5 overnight. 1.62 g of compound **37** were obtained. Yield: 69 %. ^{1}H-NMR (400 MHz, $CDCl_3$) δ (ppm): 8.10 (s, 1H), 7.03 (s, 1H), 5.70 (s, 1H), 4.62 (s, 1H), 4.43 (t, J = 7.1 Hz, 2H), 3.15 (q, J = 6.6 Hz, 2H), 1.96 (m, 2H), 1.51 (m, 2H), 1.43 (s, 9H).

***tert*-butyl (3-(4-cyano-1*H*-1,2,3-triazol-1-yl)propyl)carbamate (40)**

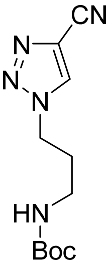

40

A solution of *tert*-butyl (3-(4-carbamoyl-1*H*-1,2,3-triazol-1-yl)propyl)carbamate (**36**) (1.46 g, 5.4 mmol) and 1.0 mL of triethylamine in 20 mL of dichloromethane was stirred at room temperature. The resulting solution was then cooled to 0-5 ºC in an ice bath. To this cooled solution, trifluoroacetic anhydride (2.2 g, 10.8 mmol) was added. The resulting solution was stirred for 5 h. After completion (monitored by TLC), solvent was removed *in vacuo* and distilled water added to the residue and then extracted with 3 portions (3x10mL) of dichloromethane. The organic layer was washed with saturated $NaHCO_3$ solution, dried ($MgSO_4$) and concentrated *in vacuo* to yield 1.3 g of the desired compound. Yield: 96 %. ^{1}H-NMR (400 MHz, $CDCl_3$) δ (ppm): 8.19 (s, 1H), 4.48 (t, *J* = 6.7 Hz, 2H), 3.77 (t, *J* = 6.6 Hz, 2H), 2.30 (m, 2H), 1.52 (s, 9H).

***tert*-butyl (4-(4-cyano-1*H*-1,2,3-triazol-1-yl)butyl)carbamate (41)**

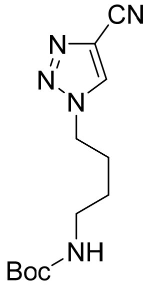

41

A solution of *tert*-butyl (4-(4-carbamoyl-1*H*-1,2,3-triazol-1-yl)butyl)carbamate (**37**) (1.46 g, 5.4 mmol) and 1.0 mL of triethylamine in 20 mL of dichloromethane was stirred at room temperature.

The resulting solution was then cooled to 0-5 ºC in an ice bath. To this cooled solution, trifluoroacetic anhydride (2.2 g, 10.8 mmol) was added. The resulting solution was stirred for 5 h. After completion (monitored by TLC), solvent was removed *in vacuo* and distilled water added to the residue and then extracted with 3 portions (3x10mL) of dichloromethane. The organic layer was washed with saturated $NaHCO_3$ solution, dried ($MgSO_4$) and concentrated *in vacuo* to yield 1.31 g of the desired compound. Yield: 92%. ^{1}H-NMR (400 MHz, $CDCl_3$) δ (ppm): 8.08 (s, 1H), 4.48 (t, J = 7.1 Hz, 2H), 3.74 (t, 2H), 1.96 (m, 2H), 1.62 (m, 2H), 1.51 (s, 9H).

***tert*-butyl (*Z*)-(3-(4-(*N′*-hydroxycarbamimidoyl)-1*H*-1,2,3-triazol-1-yl)propyl)carbamate (44)**

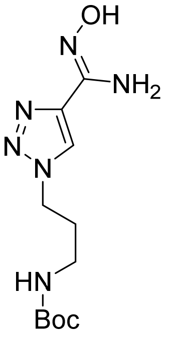

44

To a stirred solution of *tert*-butyl (3-(4-cyano-1*H*-1,2,3-triazol-1-yl)propyl)carbamate (**40**) (1.3 g, 5.1 mmol) in 20 mL methanol, hydroxylamine hydrochloride (0.72g, 10.3 mmol) and sodium bicarbonate (0.85 g, 10.1 mmol) was added. The resulting mixture was heated under reflux for 14 h. After completion (monitored by TLC), solvent was removed *in vacuo* and distilled water added to the residue. The mixture was filtrated under vacuum, and the white solid was washed with distilled water (2x10 mL) and ethyl acetate (2x10 mL) and dried overnight in the presence of P_2O_5. 1.04 g of compound **44** were obtained. Yield: 72 %. ^{1}H-NMR (400 MHz, *d*$_6$-DMSO) δ (ppm): 9.47 (s, 1H), 8.24 (s, 1H), 6.91 (t, J = 5.7 Hz, 1H), 5.69 (s, 2H), 4.34 (t, J = 7.0 Hz, 2H), 2.89 (q, J = 6.5 Hz, 2H), 1.90 (m, 2H), 1.35 (s, 9H).

***tert*-butyl (*Z*)-(4-(4-(*N'*-hydroxycarbamimidoyl)-1*H*-1,2,3-triazol-1-yl)butyl)carbamate (45)**

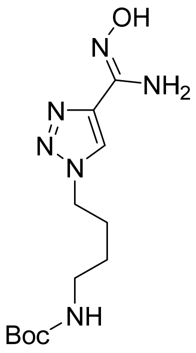

45

To a stirred solution of *tert*-butyl (4-(4-cyano-1*H*-1,2,3-triazol-1-yl)butyl)carbamate (**41**) (1.3 g, 5.1 mmol) in 20 mL methanol, hydroxylamine hydrochloride (0.72g, 10.3 mmol) and sodium bicarbonate (0.85 g, 10.1 mmol) was added. The resulting mixture was heated under reflux for 14 h. After completion (Monitored by TLC), solvent was removed *in vacuo* and distilled water added to the residue. The mixture was filtrated under vacuum, and the white solid was washed with distilled water (2x10 mL) and ethyl acetate (2x10 mL) and dried overnight in the presence of P_2O_5. 1.09 g of compound **45** were obtained. Yield: 72 %. ^{1}H-NMR (400 MHz, *d*$_6$-DMSO) δ (ppm): 9.59 (s, 1H), 8.27 (s, 1H), 6.81 (t, *J* = 6.7 Hz, 1H), 5.87 (s, 2H), 4.34 (t, *J* = 7.1 Hz, 2H), 2.89 (q, *J* = 6.7 Hz, 2H), 1.76 (m, 2H), 1.33 (s, 9H), 1.29 (m, 2H). ^{13}C-NMR (100 MHz, *d*$_6$-DMSO) δ (ppm): 156.0, 145.8, 140.6, 122.7, 77.9, 49.7, 41.6, 27.5, 26.8, 11.3.

***tert*-butyl (3-(4-(5-oxo-4,5-dihydro-1,2,4-oxadiazol-3-yl)-1*H*-1,2,3-triazol-1-yl)propyl)carbamate (48)**

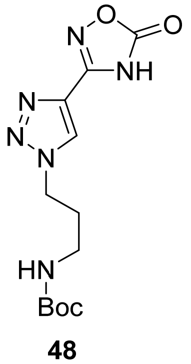

48

To a suspension of *tert*-butyl (*Z*)-(3-(4-(N'-hydroxycarbamimidoyl)-1*H*-1,2,3-triazol-1-yl)propyl)carbamate (**44**) (0.15 g, 0.50 mmol) in anhydrous acetonitrile (5 mL) *N,N'*-carbonyldiimidazole (CDI) (0.10 g, 0.65 mmol) and DBU (0.1 mL) were added at room temperature. The mixture was stirred for 4 h under reflux. After completion (Monitored by TLC), 10% (v/v) acetic acid (25 mL) was added, and the mixture was extracted with ethyl acetate (3x10 mL). The combined organic layers washed with distilled water (3x10 mL), dried ($MgSO_4$) and concentrated *in vacuo* to afford 82 mg of **48**. Yield: 53 %. ^{1}H-NMR (400 MHz, d_6-DMSO) δ (ppm): 13.21 (s, 1H), 8.76 (s, 1H), 6.92 (t, J = 5.7 Hz, 1H), 4.45, (t, J = 7.0 Hz, 2H), 2.91 (q, J = 6.4 Hz, 2H), 1.95 (m, 2H), 1.34 (s, 9H). ^{13}C-NMR (100 MHz, d_6-DMSO) δ (ppm): 160.2, 156.1, 152.3,132.4, 125.8, 78.2, 48.3, 37.4, 30.4, 28.6. IR (cm^{-1}): 3336.33, 3133.23, 2981.57, 2783.88, 1787.26, 1661.54, 1562.54, 1338.59, 1155.09, 1088.63, 924.13, 891.55, 758.80. HRMS (ESI-FIA-TOF): m/z calculated for $C_{12}H_{19}N_6O_4$ 311.1462, found 311.1462.

***tert*-butyl (4-(4-(5-oxo-4,5-dihydro-1,2,4-oxadiazol-3-yl)-1*H*-1,2,3-triazol-1-yl)butyl)carbamate (49)**

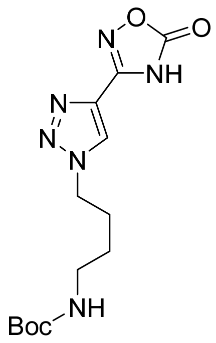

49

To a suspension of *tert*-butyl (Z)-(4-(4-(*N*'-hydroxycarbamimidoyl)-1*H*-1,2,3-triazol-1-yl)butyl)carbamate (**45**) (0.15 g, 0.50 mmol) in anhydrous acetonitrile (5 mL) *N,N'*-carbonyldiimidazole (CDI) (0.10 g, 0.65 mmol) and DBU (0.1 mL) were added at room temperature. The mixture was stirred for 4 h under reflux. After completion (monitored by TLC), 10% (v/v) acetic acid (25 mL) was added, and the mixture was extracted with ethyl acetate (3x10 mL). The combined organic layers washed with distilled water (3x10 mL), dried ($MgSO_4$) and concentrated *in vacuo*. to afford 121 mg of **49**. Yield: 75 %. ^{1}H-NMR (400 MHz, d_6-DMSO) δ (ppm): 8.78 (s, 1H), 6.80 (t, *J* = 5.8 Hz, 1H), 4.45 (t, *J* = 7.0 Hz, 2H), 2.90 (q, *J* = 6.6 Hz, 2H), 1.81 (m, 2H), 1.33 (s, 9H), 1.31 (m, 2H). ^{13}C-NMR (100 MHz, d_6-DMSO) δ (ppm): 160.0, 156.0, 152.1, 132.3, 125.6, 77.9, 50.1, 39.6, 28.6, 27.4, 26.7. HRMS (ESI-FIA-TOF): m/z calculated for $C_{13}H_{21}N_6O_4$ 325.1619, found 325.1619.

3-(1-(3-aminopropyl)-1*H*-1,2,3-triazol-4-yl)-1,2,4-oxadiazol-5(4*H*)-one hydrochloride (2)

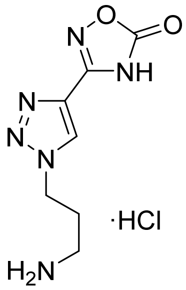

2

A mixture of *tert*-butyl (3-(4-(5-oxo-4,5-dihydro-1,2,4-oxadiazol-3-yl)-1*H*-1,2,3-triazol-1-yl)propyl)carbamate (**48**) (60 mg, 0.2 mmol) and 3M HCl in methanol (5 mL) was stirred at room temperature for 2 h. The solvent was removed *in vacuo* and the resulting yellow solid was triturated with ethyl acetate to provide 41 mg of the hydrochloride as a white solid. Yield: 86%. ^{1}H-NMR (400 MHz, d_6-DMSO) δ (ppm): 13.29 (s, 1H), 8.85 (s, 1H), 7.99 (s, 3H), 4.58 (t, J = 6.8 Hz, 2H), 2.78 (q, J = 7.5, 7.0 Hz, 2H), 2.15 (m, 2H). ^{13}C-NMR (100 MHz, d_6-DMSO) δ (ppm): 159.9, 152.0, 132.4, 126.1, 47.7, 36.5, 28.0. IR (cm^{-1}): 3165.72, 2870.49, 1797.92, 1558.51, 1208.58, 1043.91, 931.82, 783.92, 710.18. HRMS (ESI-FIA-TOF): m/z calculated for $C_7H_{11}N_6O_2$ 211.0938, found 211.0938.

3-(1-(4-aminobutyl)-1*H*-1,2,3-triazol-4-yl)-1,2,4-oxadiazol-5(4*H*)-one hydrochloride (3)

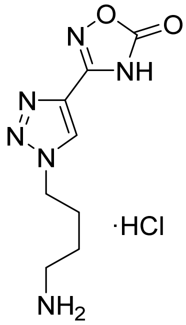

3

A mixture of *tert*-butyl (4-(4-(5-oxo-4,5-dihydro-1,2,4-oxadiazol-3-yl)-1*H*-1,2,3-triazol-1-yl)butyl)carbamate (**49**) (60 mg, 0.2 mmol) and 3M HCl in methanol (5 mL) was stirred at room temperature for 2 h. The solvent was removed *in vacuo* and the resulting yellow solid was triturated with ethyl acetate to provide 41 mg of the corresponding hydrochloride as a white solid. Yield: 86%. ^{1}H-NMR (400 MHz, d_6-DMSO) δ (ppm): 13.30 (s, 1H), 8.85 (s, 1H), 7.88 (s, 3H), 4.50 (t, J = 6.9 Hz, 2H), 2.78 (m, 2H), 1.91 (m, 2H), 1.49 (m, 2H). ^{13}C-NMR (100 MHz, d_6-DMSO) δ (ppm): 159.9, 152.1, 132.4, 125.9, 49.8, 38.5, 26.8, 24.3. IR (cm^{-1}): 3160.31, 2846.17, 1797.79, 1557.51, 1205.08, 930.61, 893.92, 706.24. HRMS (ESI-FIA-TOF): m/z calculated for $C_8H_{13}N_6O_2$ 225.1095, found 225.1091.

4-(((*tert*-butoxycarbonyl)amino)methyl)cyclohexyl methanesulfonate (26)

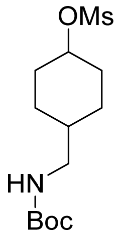

26

To a stirred solution of *tert*-butyl ((4-hydroxycyclohexyl)methyl)carbamate (**25**) (4.8 mmol) in 10 mL dichloromethane was added triethylamine (1 mL) and stirred at room temperature for 5 minutes and then cooled to 0-5 °C. Methanesulfonyl chloride (0.67 g, 6.2 mmol) was added dropwise at 0-5 °C and after addition the reaction mixture was allowed to warm to room temperature and then stirred at room temperature for 2 h. After completion (monitored by TLC), the reaction was quenched by addition of distilled water, additional dichloromethane was added and the dichloromethane layer after extraction was separated, dried ($MgSO_4$) and concentrated *in vacuo* to afford 1.40 g of compound **26**. Yield: 95%. ^{1}H-NMR (400 MHz, $CDCl_3$) δ (ppm): 4.62

(s, 1H), 4.54 (m, 1H), 2.97 (s, 3H), 2.94 (t, J = 6.5 Hz, 2H), 2.13 (m, 2H), 1.80 (m, 2H), 1.53 (m, 2H), 1.40 (s, 9H), 1.37 (m, 1H), 1.03 (m, 2H).

***tert*-butyl ((4-azidocyclohexyl)methyl)carbamate (27)**

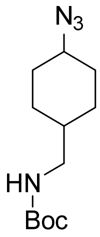

27

To a stirred solution of 4-(((*tert*-butoxycarbonyl)amino)methyl)cyclohexyl methanesulfonate (**26**) (8.9 mmol) in 20 mL dimethyl formamide, sodium azide (1.74, 26.8 mmol) was added and the resulting suspension was stirred at 80 °C for 8 h. After completion (monitored by TLC), the reaction was allowed to cool to room temperature and then poured into cold water with occasional stirring. The residue was extracted with of ethyl acetate (3x10 mL). The combined organic layers were concentrated *in vacuo* and the residue was dissolved in hexane (50 mL) and an extraction with water (3x10 mL) was made. The combined organic layers were dried ($MgSO_4$) and concentrated *in vacuo* to afford 2.01g of **27**. Yield: 89%. ^{1}H-NMR (400 MHz, $CDCl_3$) δ (ppm): 4.62 (s, 1H), 3.79 (m, 1H), 2.97 (t, J = 6.4 Hz, 2H), 1.77 (m, 2H), 1.52 (m, 3H), 1.41 (s, 11H), 1.28 (m, 2H).

ethyl 1-(4-(((*tert*-butoxycarbonyl)amino)methyl)cyclohexyl)-1*H*-1,2,3-triazole-4-carboxylate (28)

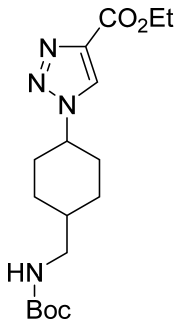

28

tert-butyl ((4-azidocyclohexyl)methyl)carbamate (**27**) (3.0 mmol) was taken in acetonitrile (15 mL) and to it ethyl propiolate (0.33 g, 3.3 mmol) was added, followed by CuI (0.11 g, 0.6 mmol) and the resulting solution was stirred at room temperature for 15 h. After completion (monitored by TLC), solvent was removed *in vacuo* and distilled water was added to the residue and then extracted with 3 portions (3x10 mL) of ethyl acetate. The combined organic layers were dried ($MgSO_4$) and concentrated *in vacuo* to obtain 0.84 g the desired compound as a pale yellow solid. Yield: 89%. ^{1}H-NMR (400 MHz, $CDCl_3$) δ (ppm): 8.12 (s, 1H), 4.62 (m, 1H), 4.59 (m, 1H), 4.39 (q, J = 7.1 Hz, 2H), 3.09 (t, J = 6.8 Hz, 2H), 2.17 (m, 2H), 2.00 (m, 2H), 1.68 (m, 2H), 1.52 (m, 2H), 1.41 (s, 10H), 1.38 (m, 3H).

***tert*-butyl ((4-(4-carbamoyl-1*H*-1,2,3-triazol-1-yl)cyclohexyl)methyl)carbamate (38)**

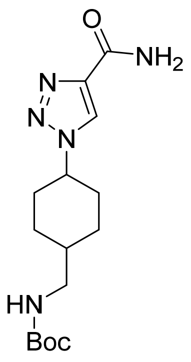

38

To a solution of ethyl 1-(4-(((*tert*-butoxycarbonyl)amino)methyl)cyclohexyl)-1*H*-1,2,3-triazole-4-carboxylate (**28**) (8.3 mmol) in 20 mL of ethanol, 30 mL of aqueous ammonia were added, and the resulting solution was stirred at room temperature for 12 h. After completion (Monitored by TLC), distilled water was added, and the mixture was filtrated under vacuum. The resulting white solid was washed with distilled water (3x10 mL) and dried under vacuum and in the presence of P_2O_5 overnight to provide 1.95 g of compound **38**. Yield. 73%. ^{1}H-NMR (400 MHz, $CDCl_3$) δ (ppm): 8.23 (s, 1H), 7.08 (s, 1H), 5.84 (s, 1H), 4.63 (t, J = 6.1 Hz, 1H), 4.58 (m, 1H), 3.09 (t, J = 6.8 Hz, 2H), 2.23 (m, 2H), 1.99 (m, 2H), 1.78 (m, 1H), 1.68 (m, 1H), 1.51 (m, 3H), 1.42 (s, 9H).

***tert*-butyl ((4-(4-cyano-1*H*-1,2,3-triazol-1-yl)cyclohexyl)methyl)carbamate (42)**

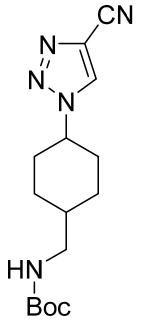

42

A solution of *tert*-butyl ((4-(4-carbamoyl-1*H*-1,2,3-triazol-1-yl)cyclohexyl)methyl)carbamate (**38**) (5.4 mmol) and 1.0 mL of triethylamine in 20 mL of dichloromethane was stirred at room temperature. The resulting solution was then cooled to 0-5 °C in an ice bath. To this cooled solution, trifluoroacetic anhydride (2.2 g, 10.8 mmol) was added. The resulting solution was stirred for 5 h. After completion (monitored by TLC), solvent was removed *in vacuo* and distilled water added to the residue and then extracted with 3 portions (3x10mL) of dichloromethane. The organic layer was washed with saturated $NaHCO_3$ solution, dried ($MgSO_4$) and concentrated *in vacuo* to yield 1.58 g of the desired compound. Yield: 96%. ^{1}H-NMR (400 MHz) δ (ppm): 8.23 (s, 1H),

4.60 (m, 1H), 3.71 (d, J = 7.5 Hz, 2H), 3.09 (m, 1H), 2.20 (m, 2H), 2.03 (m, 4H), 1.68 (m, 2H), 1.51 (s, 10H).

***tert*-butyl (*Z*)-((4-(4-(N'-hydroxycarbamimidoyl)-1*H*-1,2,3-triazol-1-yl)cyclohexyl)methyl)carbamate (46)**

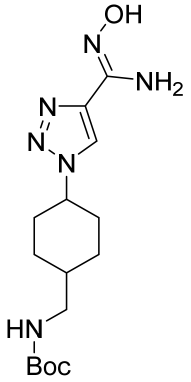

46

To a stirred solution of *tert*-butyl ((4-(4-cyano-1*H*-1,2,3-triazol-1-yl)cyclohexyl)methyl)carbamate (**42**) (1.3 g, 5.1 mmol) in 20 mL methanol, hydroxylamine hydrochloride (0.72g, 10.3 mmol) and sodium bicarbonate (0.85 g, 10.1 mmol) was added. The resulting mixture was heated under reflux for 14 h. After completion (monitored by TLC), solvent was removed *in vacuo* and distilled water added to the residue. The mixture was filtrated under vacuum, and the white solid was washed with distilled water (2x10 mL) and ethyl acetate (2x10 mL). After drying overnight in the presence of P_2O_5, 1.46 g of compound **46** were obtained. Yield: 85 %. ^{1}H-NMR (400 MHz, d_6-DMSO) δ (ppm): 9.44 (s, 1H), 8.28 (s, 1H), 6.83 (t, J = 6.0 Hz, 1H), 5.68 (s, 2H), 4.51 (m, 1H), 2.91 (t, J = 6.7 Hz, 2H), 2.10 (m, 2H), 1.81 (m, 2H), 1.65 (m, 1H), 1.55 (m, 2H), 1.41 (m, 2H), 1.34 (s, 9H).

***tert*-butyl ((4-(4-(5-oxo-4,5-dihydro-1,2,4-oxadiazol-3-yl)-1*H*-1,2,3-triazol-1-yl)cyclohexyl)methyl)carbamate (50)**

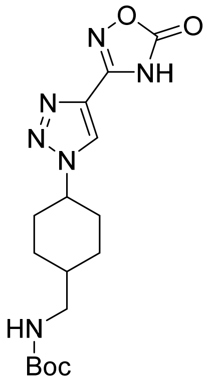

50

To a suspension *tert*-butyl (Z)-((4-(4-(*N′*-hydroxycarbamimidoyl)-1*H*-1,2,3-triazol-1-yl)cyclohexyl)methyl)carbamate (**46**) (0.50 mmol) in anhydrous acetonitrile (5 mL) *N,N'*-carbonyldiimidazole (CDI) (0.10 g, 0.65 mmol) and DBU (0.1 mL) were added at room temperature. The mixture was stirred for 15 h under reflux. After completion of the reaction (monitored by TLC), 10% (v/v) acetic acid (25 mL) was added, and the mixture was extracted with ethyl acetate (3x10 mL). The combined organic layers washed with distilled water (3x10 mL), dried ($MgSO_4$) and concentrated *in vacuo* to provide 0.15 g of **50**. Yield: 81%. ^{1}H-NMR (400 MHz, d_6-DMSO) δ (ppm): 8.85 (s, 1H), 6.84 (t, J = 6.0 Hz, 1H), 4.63 (m, 1H), 2.91 (t, J = 7.3 Hz, 2H), 2.13 (m, 2H), 1.85 (m, 2H), 1.66 (m, 1H), 1.57 (m, 2H), 1.41 (m, 2H), 1.34 (s, 9H). ^{13}C-NMR (100 MHz, d_6-DMSO) δ (ppm): 160.5, 156.2, 152.6, 132.5, 124.5, 77.8, 58.8, 43.2, 34.2, 28.7, 28.4, 25.8. IR (cm^{-1}): 3371.35, 3219.88, 2930.12, 1805.71, 1688.31, 1509.09, 1244.27, 1168.71, 930.82, 899.20, 668.88. HRMS (ESI-FIA-TOF): m/z calculated for $C_{16}H_{25}N_6O_4$ 365.1932, found 365.1932.

3-(1-(4-(aminomethyl)cyclohexyl)-1*H*-1,2,3-triazol-4-yl)-1,2,4-oxadiazol-5(4*H*)-one hydrochloride (4)

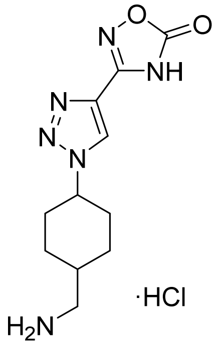

4

A mixture of *tert*-butyl ((4-(4-(5-oxo-4,5-dihydro-1,2,4-oxadiazol-3-yl)-1*H*-1,2,3-triazol-1-yl)cyclohexyl)methyl)carbamate (**50**) (0.2 mmol) and 3M HCl in methanol (5 mL) was stirred at room temperature for 2 h. The solvent was removed *in vacuo* and the resulting yellow solid was triturated with ethyl acetate to provide 41 mg of hydrochloride **4** as a white solid. Yield: 91%. ^{1}H-NMR (400 MHz, d_6-DMSO) δ (ppm): 13.28 (s, 1H), 9.00 (s, 1H), 8.05 (s, 3H), 4.69 (m, 1H), 2.84 (m, 2H), 2.13 (m, 2H), 1.90 (m, 3H), 1.67 (m, 2H), 1.54 (m, 2H). ^{13}C-NMR (100 MHz, d_6-DMSO) δ (ppm): 159.9, 152.2, 132.3, 124.7, 58.4, 41.7, 32.0, 28.7, 28.0, 25.5. IR (cm^{-1}): 3051.98, 2935.54, 1786.12, 1612.98, 1463.24, 1433.63, 926.44, 894.03, 753.45. HRMS (ESI-FIA-TOF): m/z calculated for $C_{11}H_{17}N_6O_2$ 265.1408, found 265.1410.

***tert*-butyl 4-(3-(methoxycarbonyl)-1H-1,2,4-triazol-1-yl)piperidine-1-carboxylate (52)**

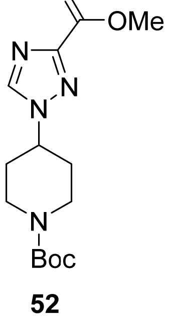

52

NaH (1 g; 41.7 mmol) was added to a solution of methyl 1*H*-1,2,4-triazole-3-carboxylate (2.8 g; 22 mmol) in DMF (130 mL). The reaction mixture was stirred at 25 °C for 20 minutes followed by 1 h at 70 °C. *tert*-butyl 4-iodopiperidine-1-carboxylate (**51**) (6 g, 19.3 mmol) was added and the reaction mixture was heated at 70 °C for 48 h. The solution was cooled to 0 °C and the insoluble material was removed by filtration. The filtrate was diluted with DCM and washed with water, brine, dried over Na_2SO_4, filtered, and evaporated to dryness. The residue was purified by chromatography over silica gel (mobile phase: petroleum ether/ethyl acetate 1/5) to render 1.50 g of compound **52**. Yield: 25%. ^{1}H-NMR (400 MHz, $CDCl_3$) δ (ppm): 7.98 (d, *J* = 0.6 Hz, 1H), 5.32 (s, 1H), 4.24 (t, *J* = 16.7 Hz, 2H), 4.01 (s, 3H), 2.91 (s, 2H), 2.17 – 2.05 (m, 2H), 1.99 (s, 2H), 1.48 (s, 9H).

tert-butyl 4-(3-(hydrazinecarbonyl)-1*H*-1,2,4-triazol-1-yl)piperidine-1-carboxylate (53)

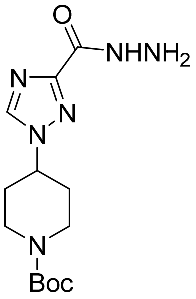

53

tert-butyl 4-(3-(methoxycarbonyl)-1*H*-1,2,4-triazol-1-yl)piperidine-1-carboxylate (**52**) (0.5 g, 1.6 mmol) and hydrazine hydrate (0.25 g) in 20 mL of *n*-butanol were refluxed for 3 h. Then, the solvent was removed by evaporation under vacuum. The residue was dissolved in dichloromethane and washed with water. The organic phase was dried ($MgSO_4$) and the solvent removed under reduced pressure. The resulting solid was washed with cold ethanol to afford 0.50 g of compound **53**. Yield: 95%. ^{1}H-NMR (400 MHz, $CDCl_3$) δ (ppm): δ 8.53 (s, 1H), 7.86 (s, 1H), 5.51 (tt, *J* =

11.4, 4.2 Hz, 1H), 4.27 (s, 2H), 4.03 (s, 2H), 2.90 (s, 2H), 2.16 – 2.02 (m, 2H), 1.96 (d, *J* = 11.8 Hz, 2H), 1.47 (s, 9H).

***tert*-butyl 4-(3-(5-oxo-4,5-dihydro-1,3,4-oxadiazol-2-yl)-1*H*-1,2,4-triazol-1-yl)piperidine-1-carboxylate (54)**

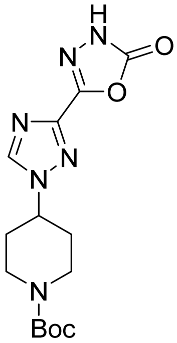

54

To a suspension of *tert*-butyl 4-(3-(hydrazinecarbonyl)-1*H*-1,2,4-triazol-1-yl)piperidine-1-carboxylate (**53**) (0.15 g, 0.50 mmol) in anhydrous acetonitrile (5 mL) *N*,*N*'-carbonyldiimidazole (CDI) (0.10 g, 0.65 mmol) and DBU (0.1 mL) were added at room temperature. The mixture was stirred for 15 h under reflux. After completion (monitored by TLC), 10% (v/v) acetic acid (25 mL) was added, and the mixture was extracted with ethyl acetate (3x10 mL). The combined organic layers washed with distilled water (3x10 mL), dried ($MgSO_4$) and concentrated *in vacuo* to render 0.15 g of compound **54**. Yield: 90%. ^{1}H-NMR (400 MHz, $CDCl_3$) δ (ppm): 8.04 (s, 1H), 5.00 (d, *J* = 11.3 Hz, 1H), 4.29 (s, 2H), 2.90 (s, 2H), 2.24 – 1.92 (m, 4H), 1.49 (s, 9H). ^{13}C-NMR (100 MHz, d_6-DMSO) δ (ppm): 153.9, 153.7, 151.2, 145.6, 139.7, 78.9, 56.6, 54.9, 31.1, 28.04. IR (cm^{-1}): 3083.51, 2972.69, 2867.28, 1818.87, 1780.92, 1638.91, 1434.60, 1367, 1268.44, 1164.10, 1133.93, 1046.99, 1006.74, 902.33, 675.17. HRMS (ESI-FIA-TOF): m/z calcd for $C_{14}H_{21}N_6O_4$ 337.1619, found 337.1621.

5-(1-(piperidin-4-yl)-1*H*-1,2,4-triazol-3-yl)-1,3,4-oxadiazol-2(3*H*)-one hydrochloride (10)

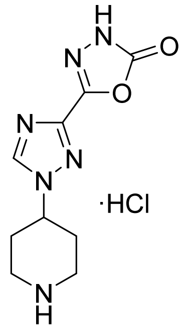

10

A mixture of *tert*-butyl 4-(3-(5-oxo-4,5-dihydro-1,3,4-oxadiazol-2-yl)-1*H*-1,2,4-triazol-1-yl)piperidine-1-carboxylate (**54**) (100 mg, 0.3 mmol) and 4N HCl in dioxane (2 mL) was stirred at room temperature for 2 h. The solvent was removed *in vacuo* and the resulting yellow solid was triturated with ethyl acetate to provide 77 mg of hydrochloride **10** as a white solid. Yield: 95%. ^{1}H-NMR (400 MHz, *d$_6$*-DMSO) δ (ppm): 13.24 (s, 1H), 9.25 (s, 1H), 9.10 (s, 1H), 8.24 (d, *J* = 0.6 Hz, 1H), 5.18 – 5.03 (m, 1H), 3.38 (d, *J* = 12.7 Hz, 2H), 3.05 (q, *J* = 11.7 Hz, 2H), 2.33 – 2.08 (m, 4H). ^{13}C-NMR (100 MHz, *d$_6$*-DMSO) δ (ppm): 153.5, 151.3, 145.4, 139.9, 54.0, 41.8, 27.9. IR (cm^{-1}): 2908.46, 2786.59, 2705.99, 2494.12, 1810.53, 1774.29, 1487.00, 1401.30, 1285.47, 1045.03, 904.69, 773.68, 724.76. HRMS (ESI-FIA-TOF): m/z calcd for $C_9H_{13}N_6O_2$ 237.1095, found 237.1094.

***tert*-butyl 4-(3-carbamoyl-1*H*-1,2,4-triazol-1-yl)piperidine-1-carboxylate (55)**

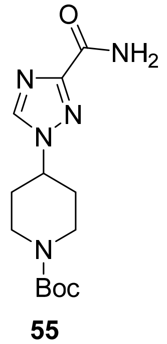

55

To a solution of *tert*-butyl 4-(3-(methoxycarbonyl)-1*H*-1,2,4-triazol-1-yl)piperidine-1-carboxylate (**52**) (0.85 g, 2.6 mmol) in 10 mL of methanol, 20 mL of ammonia in methanolic solution was added and the resulting solution was stirred at room temperature for 12 h. After completion (monitored by TLC), solvent was removed *in vacuo* and distilled water was added to the residue. It was extracted with 3 portions (3x10 mL) of dichloromethane. The combined organic layers were dried ($MgSO_4$) and concentrated *in vacuo* to render 0.75 g of the desired compound *tert*-butyl 4-(3-carbamoyl-1*H*-1,2,4-triazol-1-yl)piperidine-1-carboxylate (**55**) as a white solid. Yield: 96 %. ^{1}H-NMR (400 MHz, d_6-DMSO) δ (ppm): 13.08 (s, 2H), 8.20 (s, 1H), 5.19 – 4.89 (m, 1H), 4.19 – 3.82 (m, 2H), 2.80 (m, 2H), 1.95 (s, 2H), 1.88 – 1.69 (m, 2H), 1.39 (s, 9H).

***tert*-butyl 4-(3-cyano-1*H*-1,2,4-triazol-1-yl)piperidine-1-carboxylate (56)**

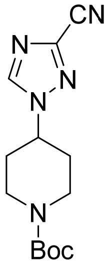

56

A solution of *tert*-butyl 4-(3-carbamoyl-1*H*-1,2,4-triazol-1-yl)piperidine-1-carboxylate (**55**) (0.9 g, 3.0 mmol) and 1.7 mL of triethylamine in 80 mL of dichloromethane was stirred at room temperature. The resulting solution was then cooled to 0-5 °C in an ice bath. To this cooled solution, trifluoroacetic anhydride (1.3 g, 6.2 mmol) was added in 20 mL of dichloromethane. The resulting solution was stirred for 5 h. After completion (monitored by TLC), solvent was removed *in vacuo* and distilled water added to the residue and then extracted with 3 portions (3x10mL) of dichloromethane. The organic layer was washed with saturated $NaHCO_3$ solution, dried ($MgSO_4$) and concentrated *in vacuo* to render 0.70 g the desired compound **56** as a brown oil. Yield: 91%.

^{1}H-NMR (400 MHz, $CDCl_3$) δ (ppm): 8.04 (s, 1H), 4.62 (tt, J = 11.4, 4.3 Hz, 1H), 4.29 (s, 2H), 2.92 (t, J = 13.1 Hz, 2H), 2.13 (dddd, J = 13.1, 12.0, 11.3, 4.5 Hz, 2H), 2.00 (m, 2H), 1.47 (s, 9H).

***tert*-butyl (*Z*)-4-(3-(*N*'-hydroxycarbamimidoyl)-1*H*-1,2,4-triazol-1-yl)piperidine-1-carboxylate (57)**

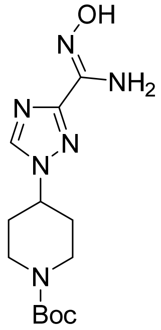

57

To a stirred solution of *tert*-butyl 4-(3-cyano-1*H*-1,2,4-triazol-1-yl)piperidine-1-carboxylate (**56**) (0.38 g, 1.3 mmol) in 10 mL methanol, hydroxylamine hydrochloride (0.47g, 6.8 mmol) and sodium bicarbonate (0.93 g, 6.8 mmol) was added. The resulting mixture was heated under reflux for 14 h. After completion (monitored by TLC), solvent was removed *in vacuo* and distilled water added to the residue and then extracted with 3 portions (3x10mL) of dichloromethane. The organic layer dried ($MgSO_4$) and concentrated *in vacuo*. A small portion of ethyl acetate was added and stirred at room temperature for 30 minutes. The resulting solid was collected by filtration and dried in vacuum to afford 0.33 g of compound **57**. Yield: 82%. ^{1}H-NMR (400 MHz, d_6-DMSO) δ (ppm): 10.21 (s, 1H), 7.99 (s, 1H), 5.93 (s, 2H), 5.25 – 5.08 (m, 1H), 4.02 (s, 2H), 2.79 (s, 2H), 1.95 – 1.72 (m, 4H), 1.39 (s, 9H).

***tert*-butyl 4-(3-(5-oxo-4,5-dihydro-1,2,4-oxadiazol-3-yl)-1*H*-1,2,4-triazol-1-yl)piperidine-1-carboxylate (58)**

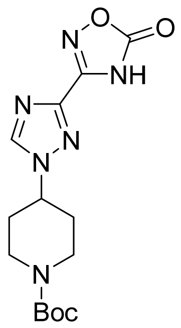

58

To a suspension of *tert*-butyl (*Z*)-4-(3-(*N*'-hydroxycarbamimidoyl)-1*H*-1,2,4-triazol-1-yl)piperidine-1-carboxylate (**57**) (0.15 g, 0.50 mmol) in anhydrous acetonitrile (5 mL) *N*,*N*'-carbonyldiimidazole (CDI) (0.10 g, 0.65 mmol) and DBU (0.1 mL) were added at room temperature. The mixture was stirred for 15 h under reflux. After completion (monitored by TLC), 10% (v/v) acetic acid (25 mL) was added, and the mixture was extracted with ethyl acetate (3x10 mL). The combined organic layers washed with distilled water (3x10 mL), dried ($MgSO_4$) and concentrated *in vacuo* to give 0.13 g of compound **58**. Yield: 78%. ^{1}H-NMR (400 MHz, d_6-DMSO) δ (ppm): 9.47 (s, 1H), 7.95 (s, 1H), 5.54 – 5.30 (m, 1H), 4.02 (d, *J* = 13.5 Hz, 2H), 2.84 (s, 2H), 1.95 – 1.70 (m, 2H), 1.70 – 1.49 (m, 2H), 1.39 (s, 9H). ^{13}C-NMR (100 MHz, d_6-DMSO) δ (ppm): 159.8, 153.7, 151.9, 132.1, 123.7, 79.0, 57.8, 42.3, 31.6, 28.0. IR (cm^{-1}): 3121.35, 2975.39, 2853.76, 1819.07, 1788.93, 1687.45, 1419.42, 1361.69, 1274.22, 1166.18, 948.42, 907.18, 733.88. HRMS (ESI-FIA-TOF): m/z calcd for $C_{14}H_{21}N_6O_4$ 337.1619, found 337.1619.

3-(1-(piperidin-4-yl)-1*H*-1,2,4-triazol-3-yl)-1,2,4-oxadiazol-5(4*H*)-one hydrochloride (9)

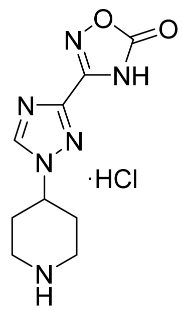

9

A mixture of *tert*-butyl 4-(3-(5-oxo-4,5-dihydro-1,2,4-oxadiazol-3-yl)-1*H*-1,2,4-triazol-1-yl)piperidine-1-carboxylate (**58**) (100 mg, 0.3 mmol) and 4N HCl in dioxane (2 mL) was stirred at room temperature for 2 h. The solvent was removed *in vacuo* and the resulting yellow solid was triturated with EtOAc to provide 77 mg of compound **9** as a yellow solid. Yield: 95%. ^{1}H-NMR (400 MHz, d_6-DMSO) δ (ppm): 9.09 (s, 2H), 8.90 (d, J = 4.5 Hz, 1H), 7.67 (s, 2H), 4.93 (s, 1H), 3.13 – 3.03 (m, 2H), 2.38 – 2.29 (m, 2H), 2.24 (s, 2H). ^{13}C-NMR (100.0 MHz, d_6-DMSO) δ (ppm): 153.5, 151.3, 145.4, 139.9, 54.0, 41.8, 27.9. IR (cm^{-1}): 3213.25, 2707.80, 1754.73, 1422.42, 1068.24, 944.82, 906.40, 762.39, 742.79, 677.38. HRMS (ESI-FIA-TOF): m/z calcd for $C_9H_{13}N_6O_2$ 237.1095, found 237.1094.

ethyl 4-(aminomethyl)cyclohexane-1-carboxylate hydrochloride (60)

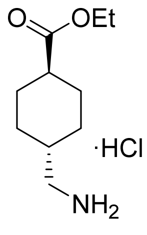

60

A solution of tranexamic acid (**59**) (4.0 g, 25.6 mmol) in ethanol (150 mL) was stirred at room temperature. The solution was then cooled to 0-5 ºC in an ice bath. To this cooled solution, thionyl chloride (9.1 g, 77 mmol) was added dropwise. The mixture was refluxed for 2 hours. After

completion, solvent was removed by evaporation and dichloromethane was added. The solution was washed with a saturated Na_2CO_3 solution and brine, dried ($MgSO_4$) and concentrated *in vacuo* to render 5.05 g of compound **60**. Yield: 89% yield. ^{1}H-NMR (400 MHz, $CDCl_3$) δ (ppm): 8.32 (s, 3H), 4.09 (q, *J* = 7.1 Hz, 2H), 2.84 (m, 2H), 2.21 (m, 1H), 2.01 (m, 4H), 1.75 (m, 1H), 1.47 (m, 2H), 1.21 (t, *J* = 7.1 Hz, 3H), 1.06 (m, 2H).

ethyl 4-(((*tert*-butoxycarbonyl)amino)methyl)cyclohexane-1-carboxylate (61)

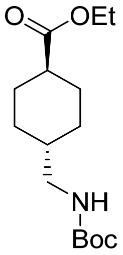

61

A mixture of ethyl 4-(aminomethyl)cyclohexane-1-carboxylate hydrochloride (**60**) (2.9 g, 13 mmol) and triethylamine (3.6 mL, 26 mmol) was dissolved in methanol (20 mL). After the addition of di-*tert*-butyl decarbonate (3.1 g, 14 mmol) the solution was stirred at room temperature for 8 hours. After completion, solvent was removed *in vacuo* and dichloromethane was added. The solution was then washed with 1M HCl solution and brine. The organic phase was dried ($MgSO_4$) and the solvent evaporated to obtain 3.52 g of compound **61**. Yield: 95%. ^{1}H-NMR (400 MHz, $CDCl_3$) δ (ppm): 4.56 (s, 1H), 4.09 (q, *J* = 7.1 Hz, 2H), 2.96 (t, 2H), 2.19 (m, 1H), 1.99 (m, 2H), 1.79 (m, 2H), 1.42 (s, 10H), 1.40 (m, 2H), 1.22 (t, *J* = 7.1 Hz, 3H), 0.95 (m, 2H).

***tert*-butyl ((4-carbamoylcyclohexyl)methyl)carbamate (62)**

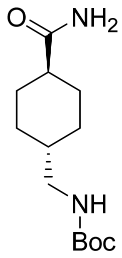

62

A solution of ethyl (4-(((*tert*-butoxycarbonyl)amino)methyl)cyclohexane-1-carboxylate (**61**) (2.6 g, 9.1 mmol) in 7N methanolic ammonia (20 mL) was stirred inside a pressurized vessel at 85 ºC for 5 days. After the addition of NaOH 1M (10 mL), the mixture was stirred at 40 ºC for 4 hours. The solvent was removed *in vacuo*, and water was added. The desired amide was extracted with ethyl acetate, dried ($MgSO_4$) and concentrated under vacuum to afford 0.58 g of compound **62**. Yield: 25%. ^{1}H-NMR (400 MHz, $CDCl_3$) δ (ppm): 5.58 (s, 2H), 4.56 (s, 1H), 2.96 (d, J = 6.6 Hz, 2H), 2.09 (m, 1H), 1.96 (m, 2H), 1.85 (m, 2H), 1.46 (m, 2H), 1.43 (s, 10H), 0.97 (m, 2H).

***tert*-butyl ((4-cyanocyclohexyl)methyl)carbamate (63)**

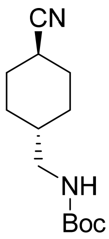

63

A solution of *tert*-butyl ((4-carbamoylcyclohexyl)methyl)carbamate (**62**) (3.0 mmol) and 1.7 mL of triethylamine in 80 mL of dichloromethane was stirred at room temperature. The resulting solution was then cooled to 0-5 °C in an ice bath. To this cooled solution, trifluoroacetic anhydride (6.2 mmol) was added in 20 mL of dichloromethane. The resulting solution was stirred for 5 h. After completion of the reaction (monitored by TLC), solvent was removed *in vacuo* and distilled

water added to the residue and then extracted with 3 portions (3x10mL) of dichloromethane. The organic layer was washed with saturated $NaHCO_3$ solution, dried ($MgSO_4$) and concentrated *in vacuo* to render 0.65 g of the desired product as a brown oil. Yield: 91%. ^{1}H-NMR (400 MHz, $CDCl_3$) δ (ppm): 3.58 (d, J = 6.8 Hz, 2H), 2.37 (m, 1H), 2.12 (m, 2H), 1.74 (m, 3H), 1.56 (m, 2H), 1.52 (s, 9H), 1.02 (m, 2H).

***tert*-butyl ((4-((*Z*)-*N*'-hydroxycarbamimidoyl)cyclohexyl)methyl)carbamate (64)**

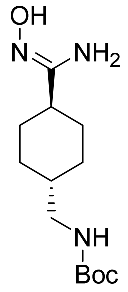

64

To a stirred solution of *tert*-butyl ((4-cyanocyclohexyl)methyl)carbamate (**63**) (1.3 mmol) in 10 mL methanol, hydroxylamine hydrochloride (6.8 mmol) and sodium bicarbonate (6.8 mmol) was added. The resulting mixture was heated under reflux for 14 h. After completion (monitored by TLC), solvent was removed *in vacuo* and distilled water added to the residue and then extracted with 3 portions (3x10mL) of dichloromethane. The organic layer dried ($MgSO_4$) and concentrated *in vacuo*. A small portion of ethyl acetate was added and stirred at room temperature for 30 minutes. The solid was collected by filtration and dried under vacuum to afford 0.29 g of compound **64**. Yield: 82%. ^{1}H-NMR (400 MHz, d_6-DMSO) δ (ppm): 8.64 (s, 1H), 6.77 (t, J = 6.1 Hz, 1H), 5.19 (s, 2H), 2.72 (q, J = 6.2 Hz, 2H), 1.86 (m, 1H), 1.68 (m, 4H), 1.34 (s, 10H), 1.28 (m, 2H), 0.82 (m, 2H).

tert-butyl ((4-(5-oxo-4,5-dihydro-1,2,4-oxadiazol-3-yl)cyclohexyl)methyl)carbamate (65)

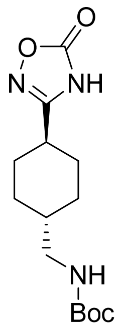

65

To a suspension of *tert*-butyl ((4-((*Z*)-*N*'-hydroxycarbamimidoyl)cyclohexyl)methyl)carbamate (**64**) (0.50 mmol) in anhydrous acetonitrile (5 mL) *N*,*N*'-carbonyldiimidazole (CDI) (0.10 g, 0.65 mmol) and DBU (0.1 mL) were added at room temperature. The mixture was stirred for 15 h under reflux. After completion (monitored by TLC), 10% (v/v) acetic acid (25 mL) was added, and the mixture was extracted with ethyl acetate (3x10 mL). The combined organic layers washed with distilled water (3x10 mL), dried ($MgSO_4$) and concentrated *in vacuo* to provide 115 mg of compound **65**. Yield: 78%. ^{1}H-NMR (400 MHz, d_6-DMSO) δ (ppm): 12.09 (s, 1H), 6.80 (t, J = 5.9 Hz, 1H), 2.75 (t, J = 6.3 Hz, 2H), 2.46 (m, 1H), 1.90 (m, 2H), 1.72 (m, 2H), 1.34 (s, 10H), 1.31 (m, 2H), 0.93 (m, 2H). ^{13}C-NMR (100 MHz, d_6-DMSO) δ (ppm): 163.4, 160.3, 156.2, 77.8, 46.8, 37.6, 34.8, 29.5, 28.7. IR (cm^{-1}): 3363.41, 2984.28, 2924.70, 1782.73, 1687.06, 1530.88, 1248.35, 1166.58, 953.70, 758.24. HRMS (ESI-FIA-TOF): m/z calculated for $C_{14}H_{24}N_3O_4$ 298.1761, found 298.1757.

3-(4-(aminomethyl)cyclohexyl)-1,2,4-oxadiazol-5(4*H*)-one hydrochloride (11)

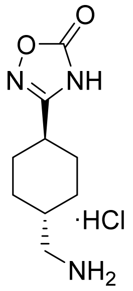

11

A mixture of *tert*-butyl ((4-(5-oxo-4,5-dihydro-1,2,4-oxadiazol-3-yl)cyclohexyl)methyl)carbamate (**65**) (0.2 mmol) and 3M HCl in methanol (5 mL) was stirred at room temperature for 2 h. The solvent was removed *in vacuo* and the resulting yellow solid was triturated with ethyl acetate to provide41 mg of hydrochloride **11** as a white solid. Yield: 88%. ^{1}H-NMR (400 MHz, d_6-DMSO) δ (ppm): 8.37 (s, 3H), 2.63 (d, J = 6.8 Hz, 2H), 2.52 (m, 1H), 1.93 (m, 2H), 1.84 (m, 2H), 1.56 (m 1H), 1.40 (m, 2H), 1.03 (m, 2H). ^{13}C-NMR (100 MHz, d_6-DMSO) δ (ppm): 165.8, 162.8, 46.7, 37.3, 36.7, 31.2, 30.5. IR (cm^{-1}): 3030.32, 2945.68, 1770.02, 1605.87, 1492.20, 1221.33, 959.10, 713.56. HRMS (ESI-FIA-TOF): m/z calculated for $C_9H_{16}N_3O_2$ 198.1237, found 198.1235.

II. NMR Spectroscopy

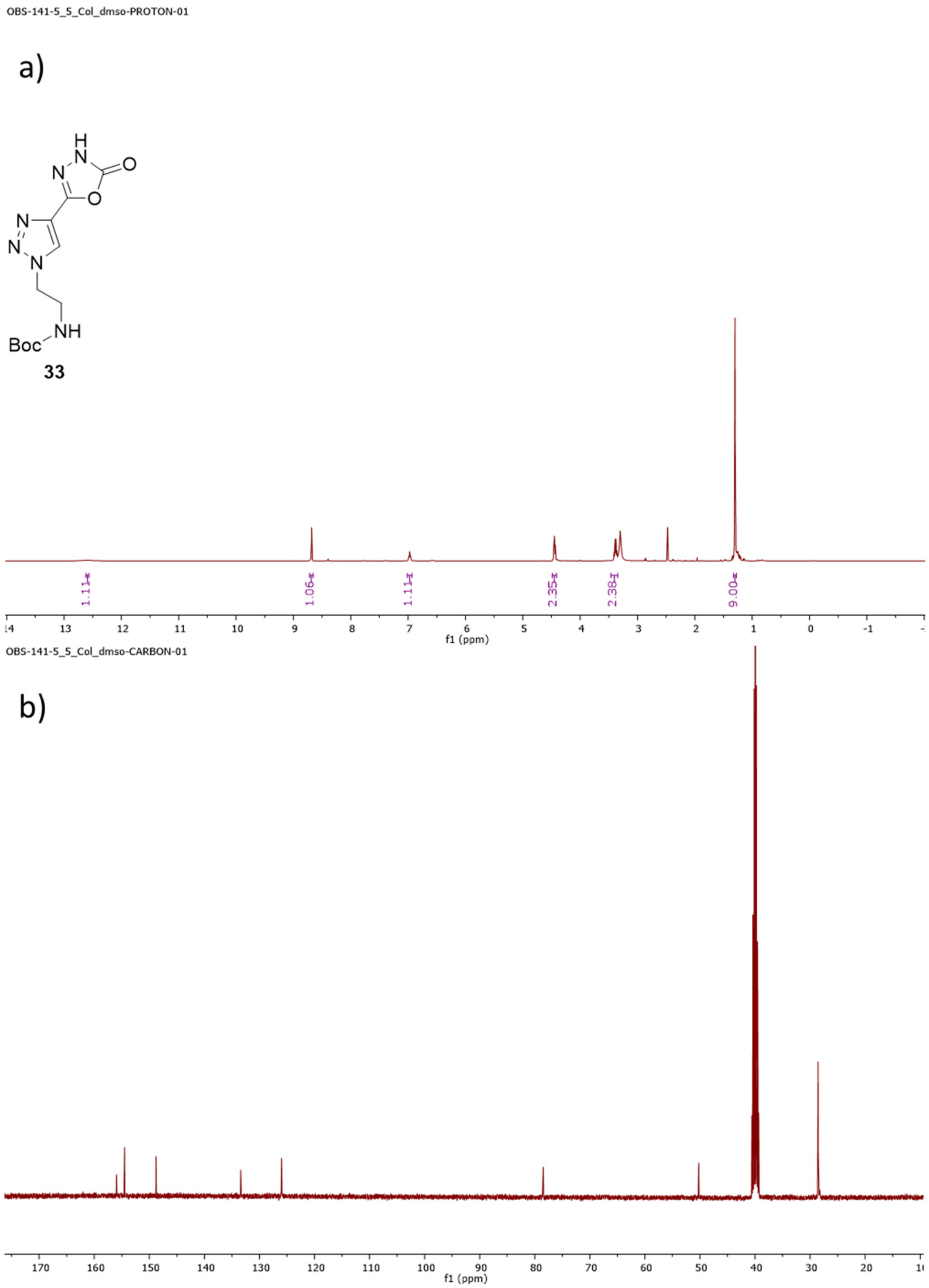

Figure S1. ^{1}H-NMR (a) and ^{13}C-NMR (b) spectra of **33** in *d*$_6$-DMSO.

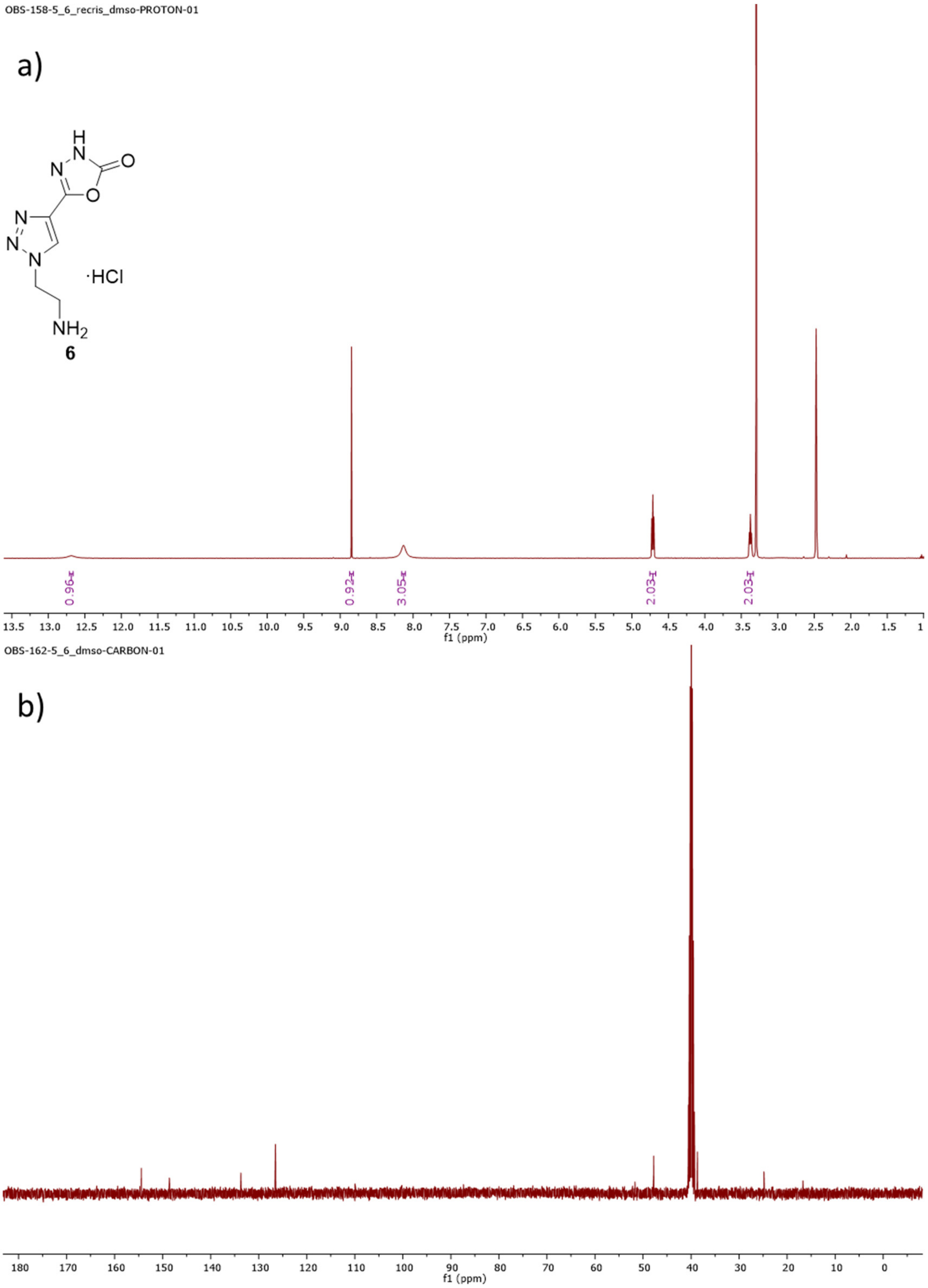

Figure S2. ^{1}H-NMR (a) and ^{13}C-NMR (b) spectra of **6** in d_6-DMSO.

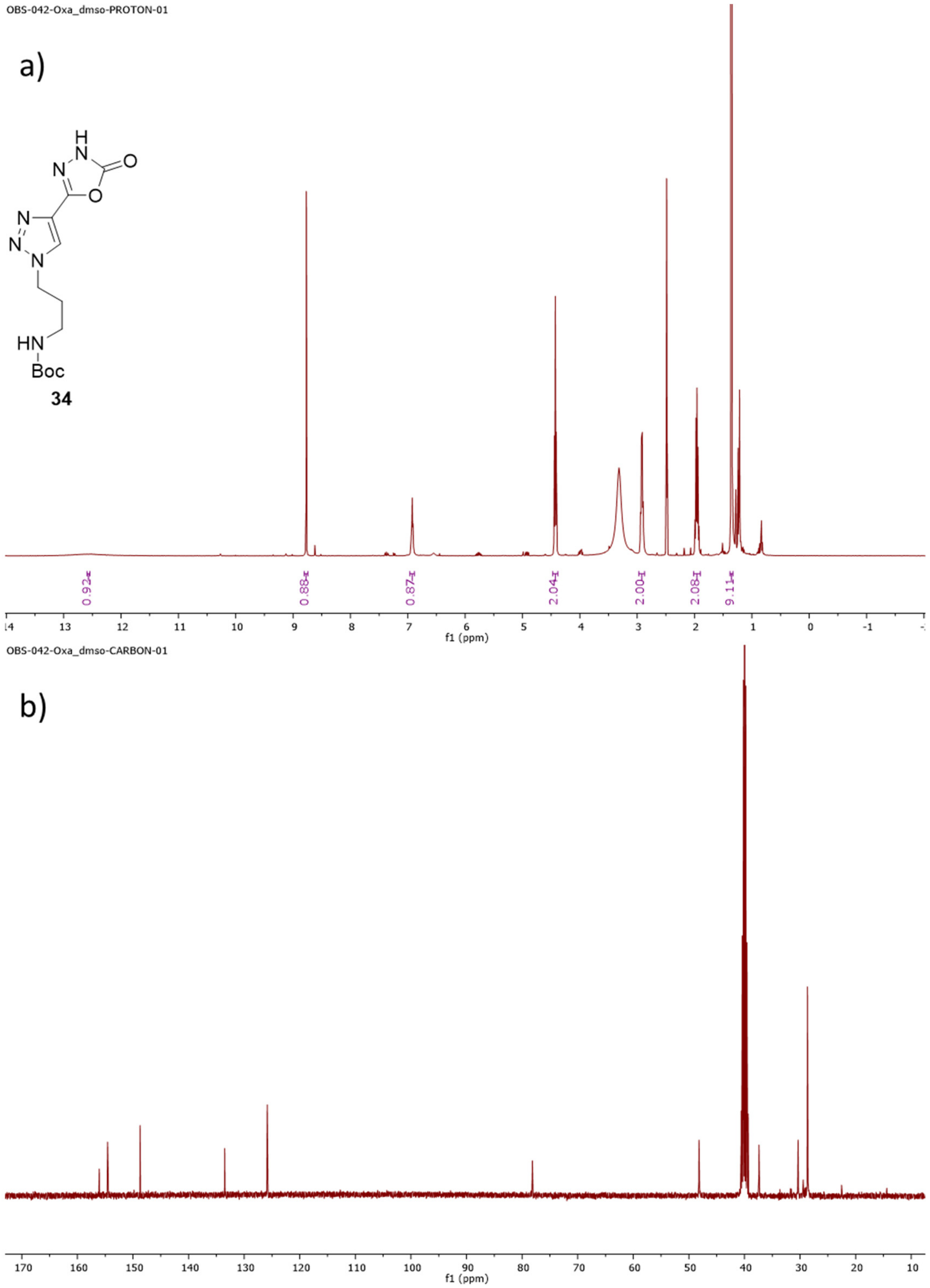

Figure S3. ^{1}H-NMR (a) and ^{13}C-NMR (b) spectra of **34** in d_6-DMSO.

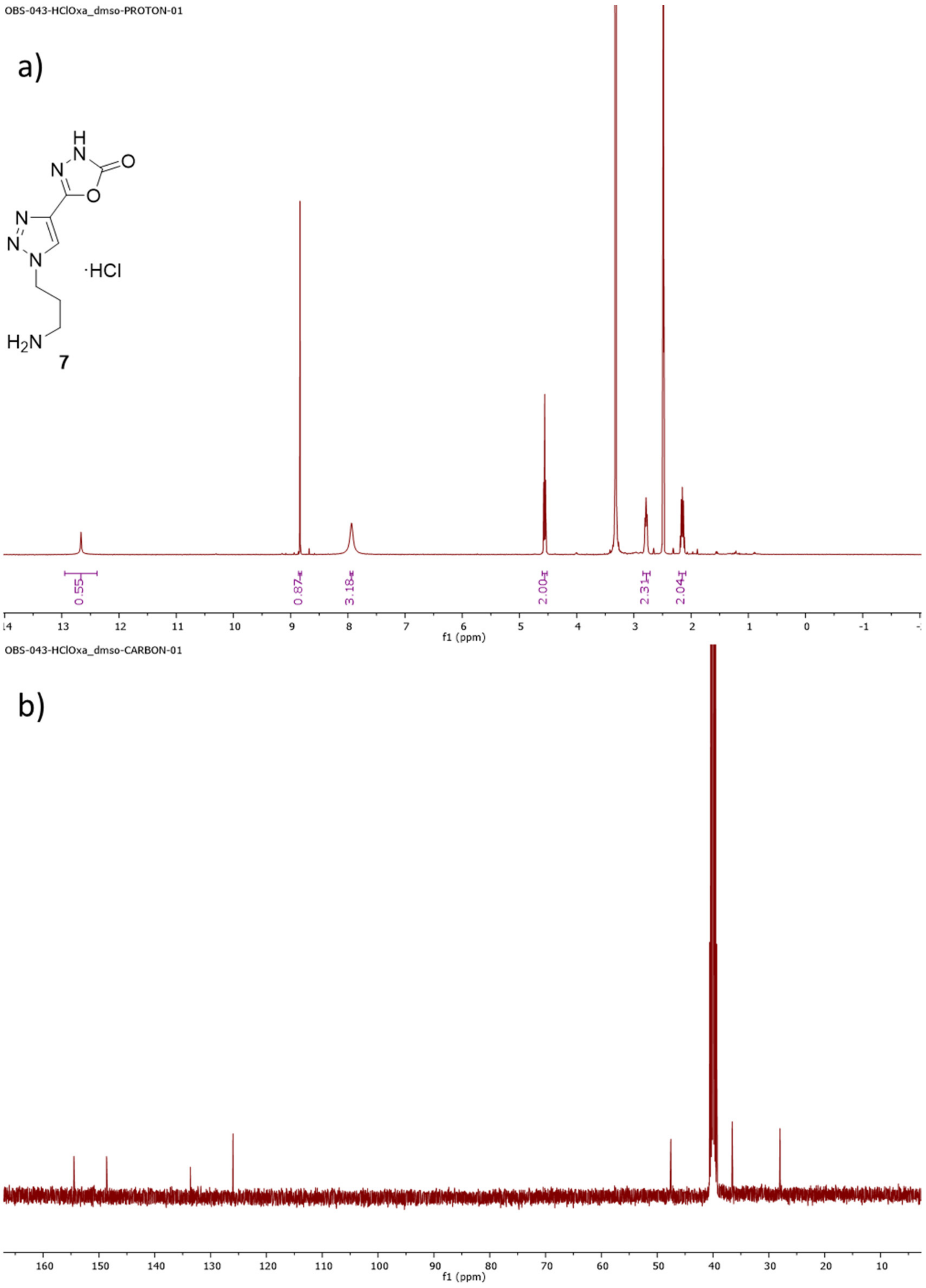

Figure S4. ^{1}H-NMR (a) and ^{13}C-NMR (b) spectra of **7** in d_6-DMSO.

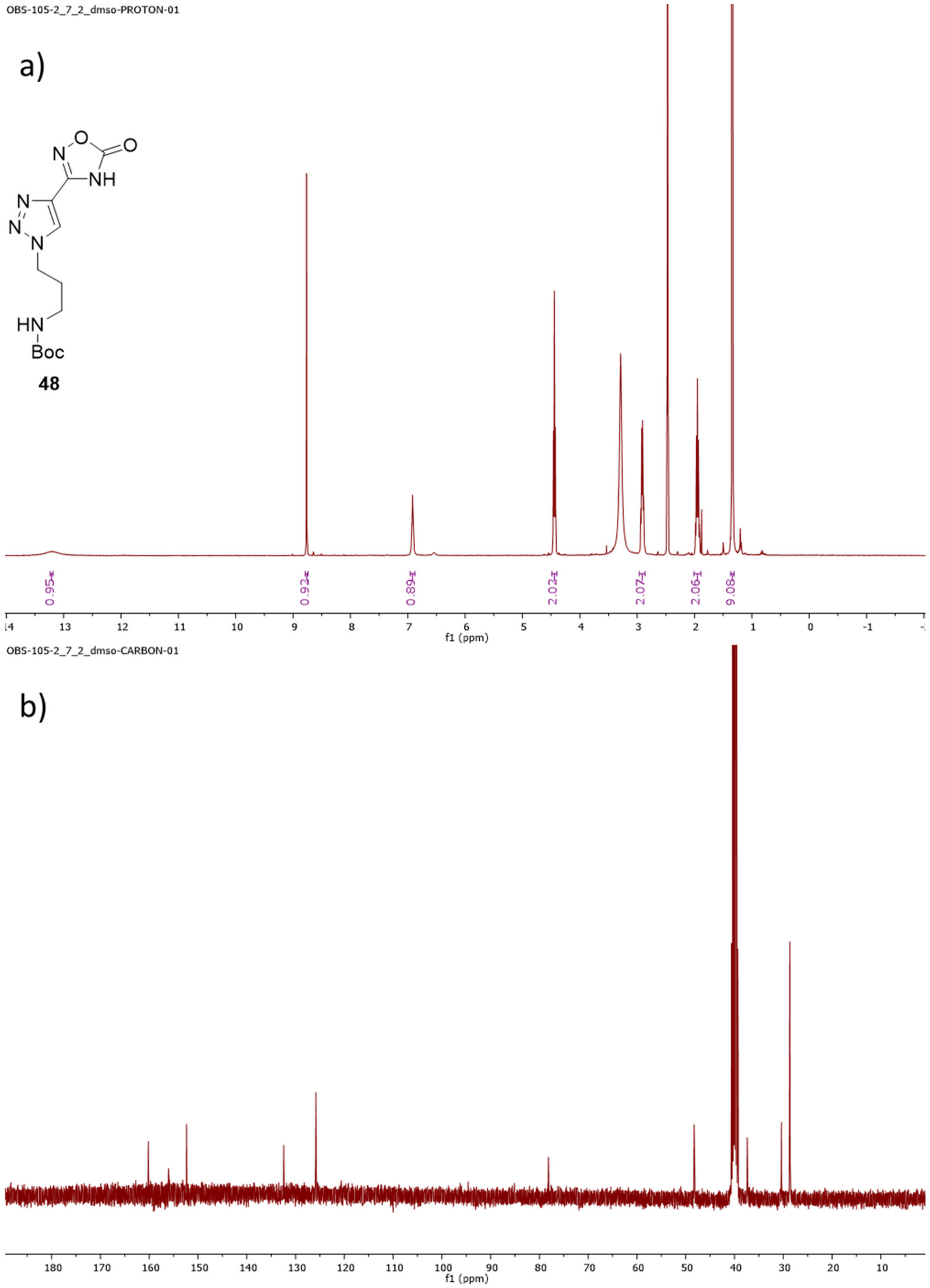

Figure S5. ^{1}H-NMR (a) and ^{13}C-NMR (b) spectra of **48** in d_6-DMSO.

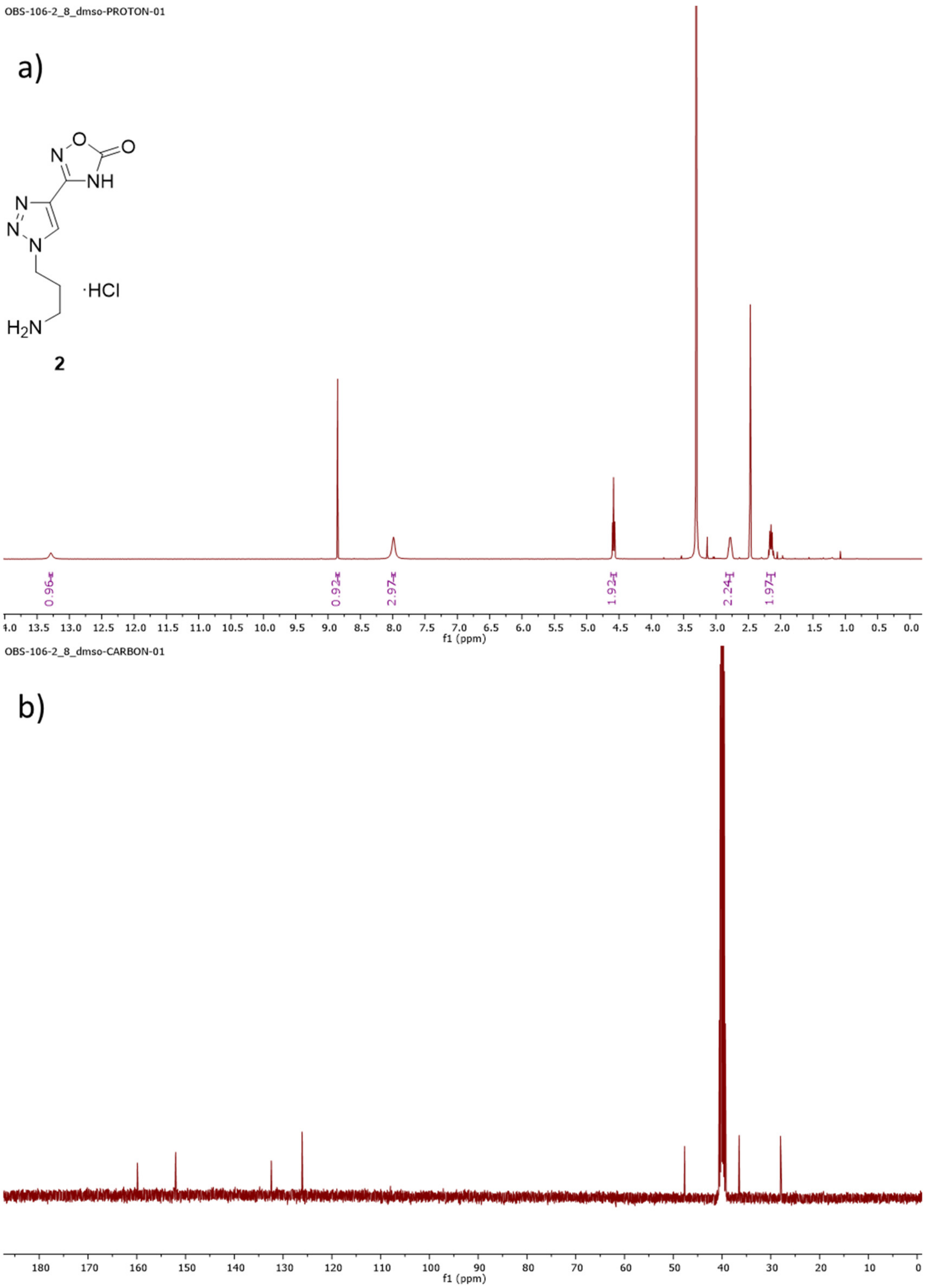

Figure S6. ^{1}H-NMR (a) and ^{13}C-NMR (b) spectra of **2** in d_6-DMSO.

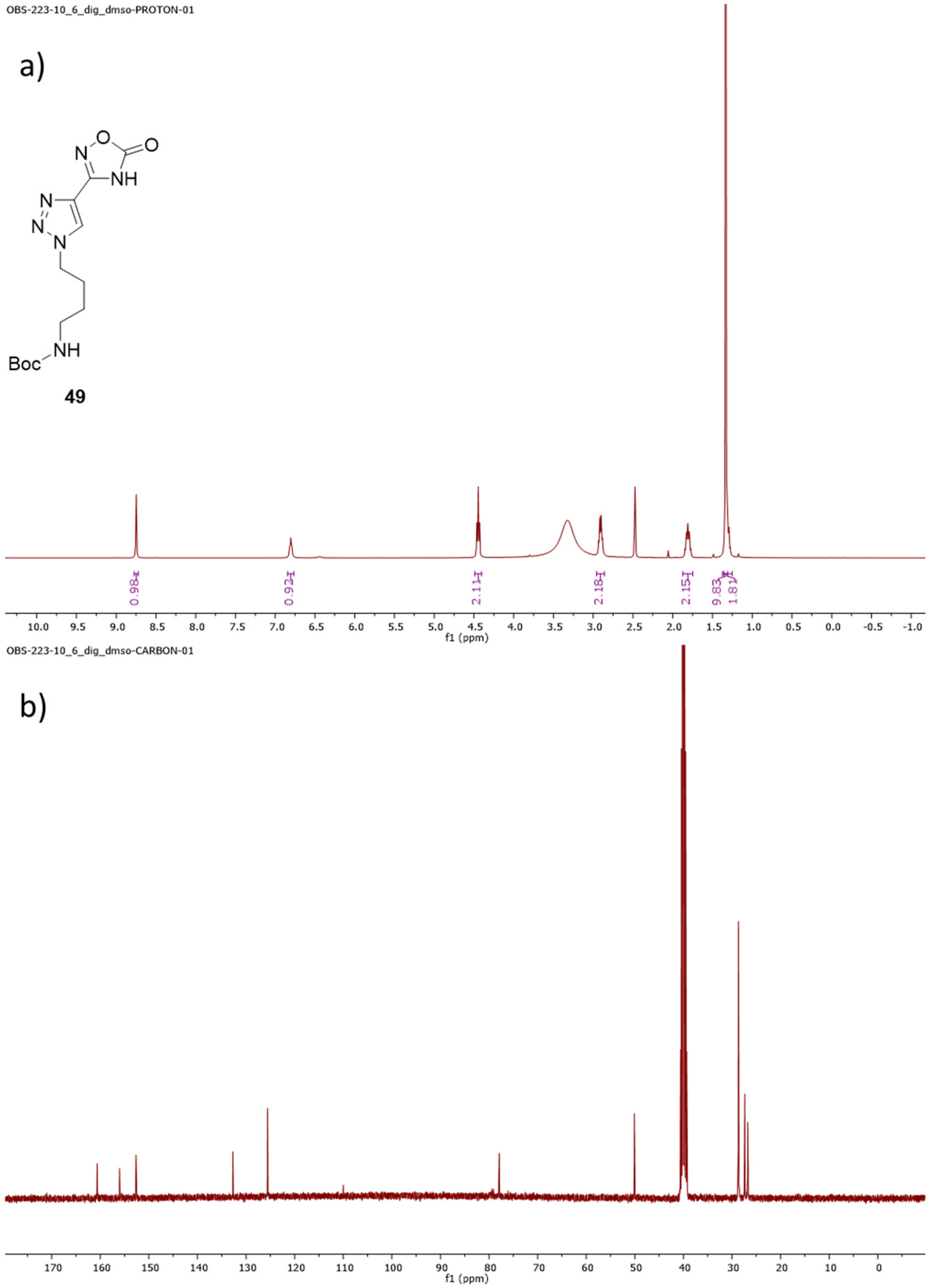

Figure S7. ^{1}H-NMR (a) and ^{13}C-NMR (b) spectra of **49** in d_6-DMSO.

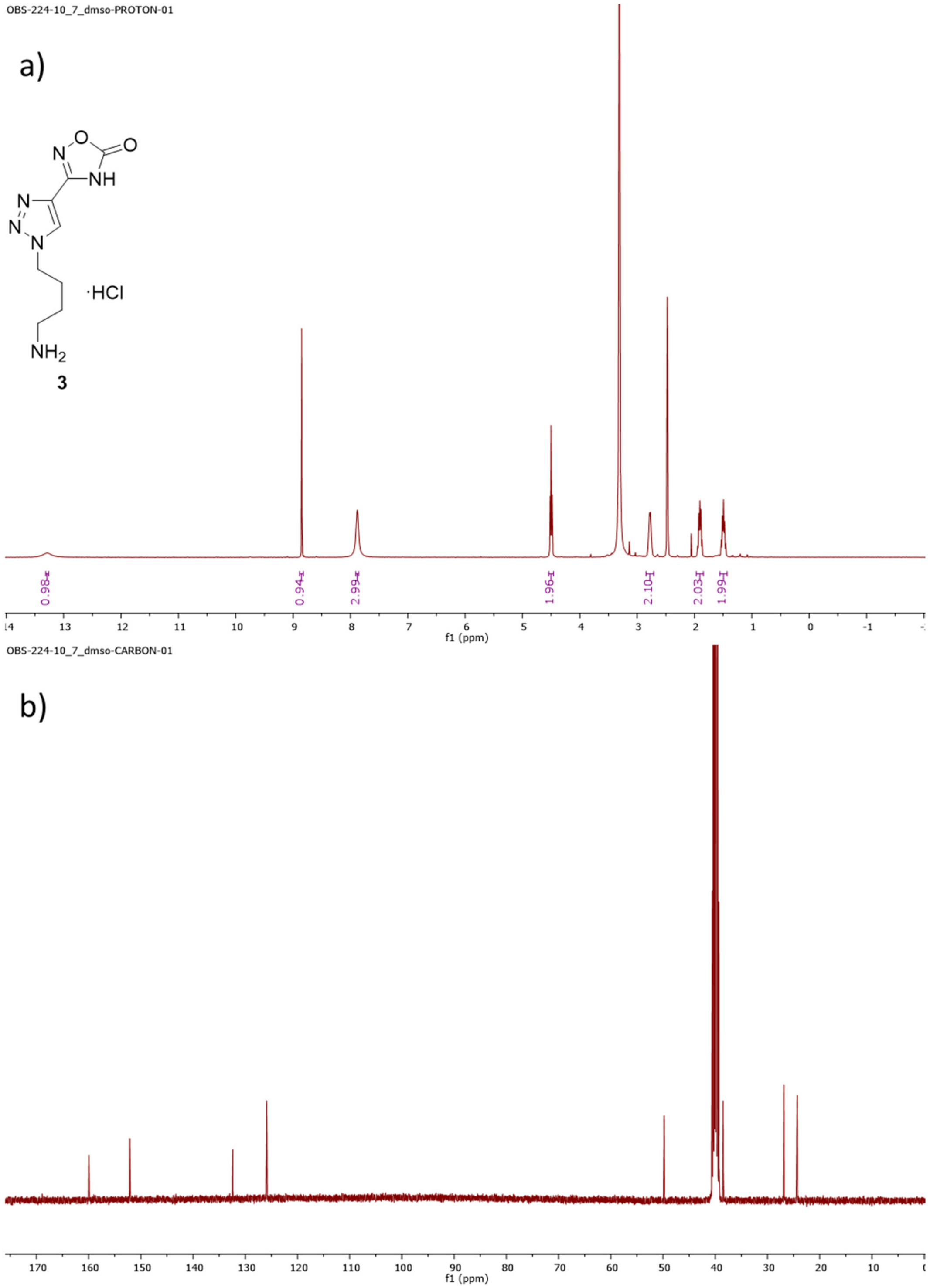

Figure S8. ^{1}H-NMR (a) and ^{13}C-NMR (b) spectra of **3** in d_6-DMSO.

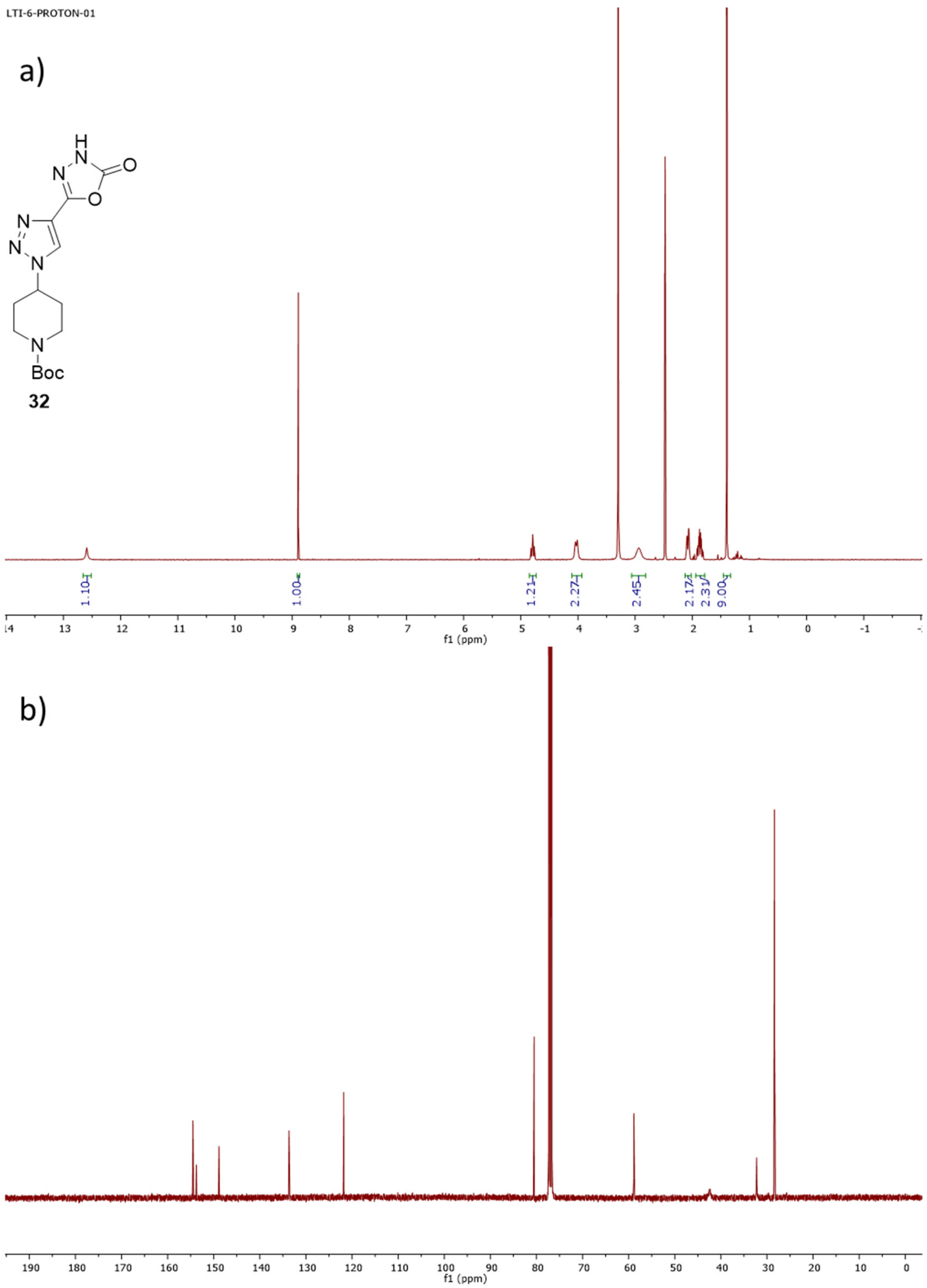

Figure S9. ^{1}H-NMR (a) and ^{13}C-NMR (b) spectra of **32** in d_6-DMSO.

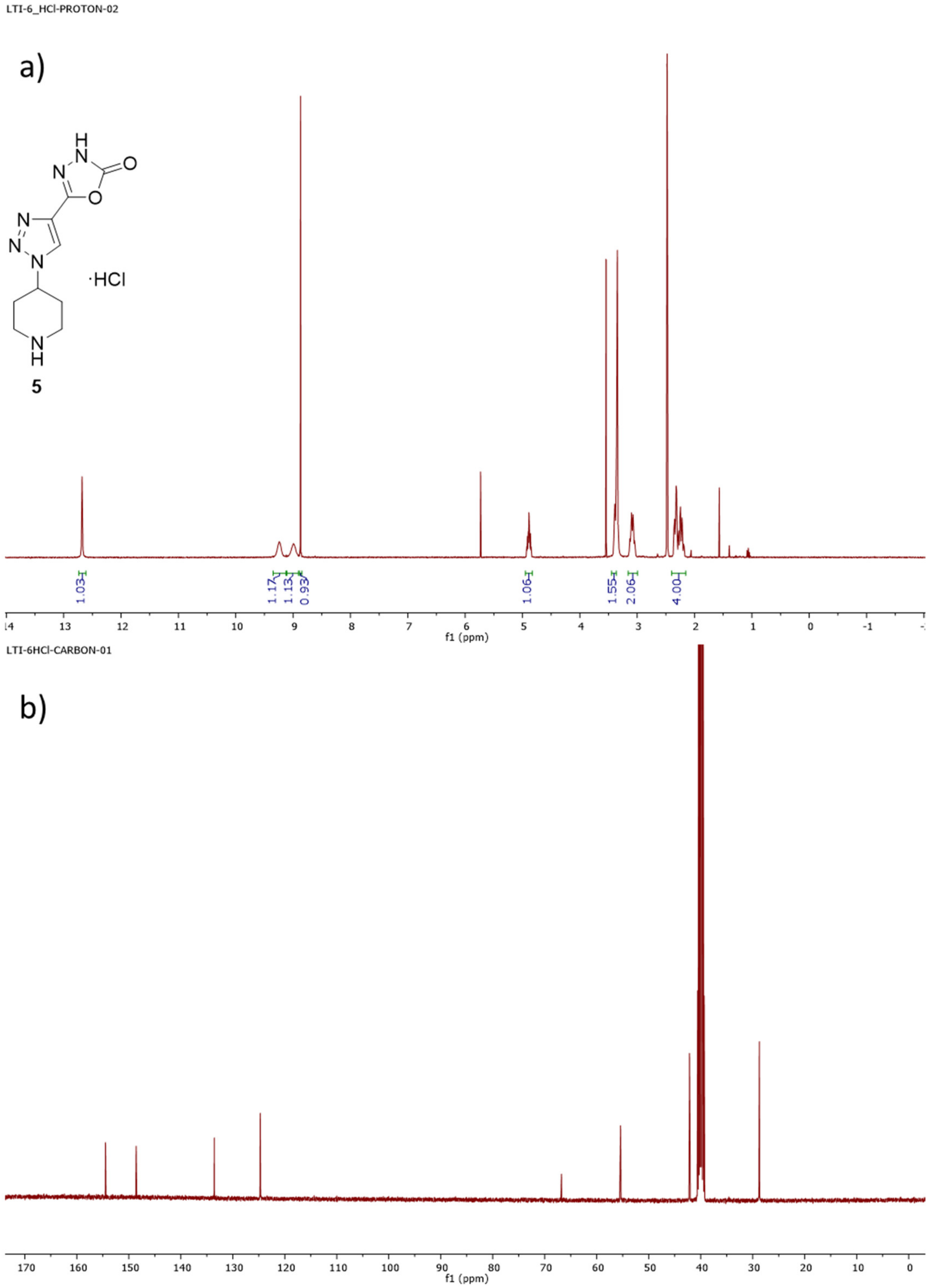

Figure S10. ^{1}H-NMR (a) and ^{13}C-NMR (b) spectra of **5** in d_6-DMSO.

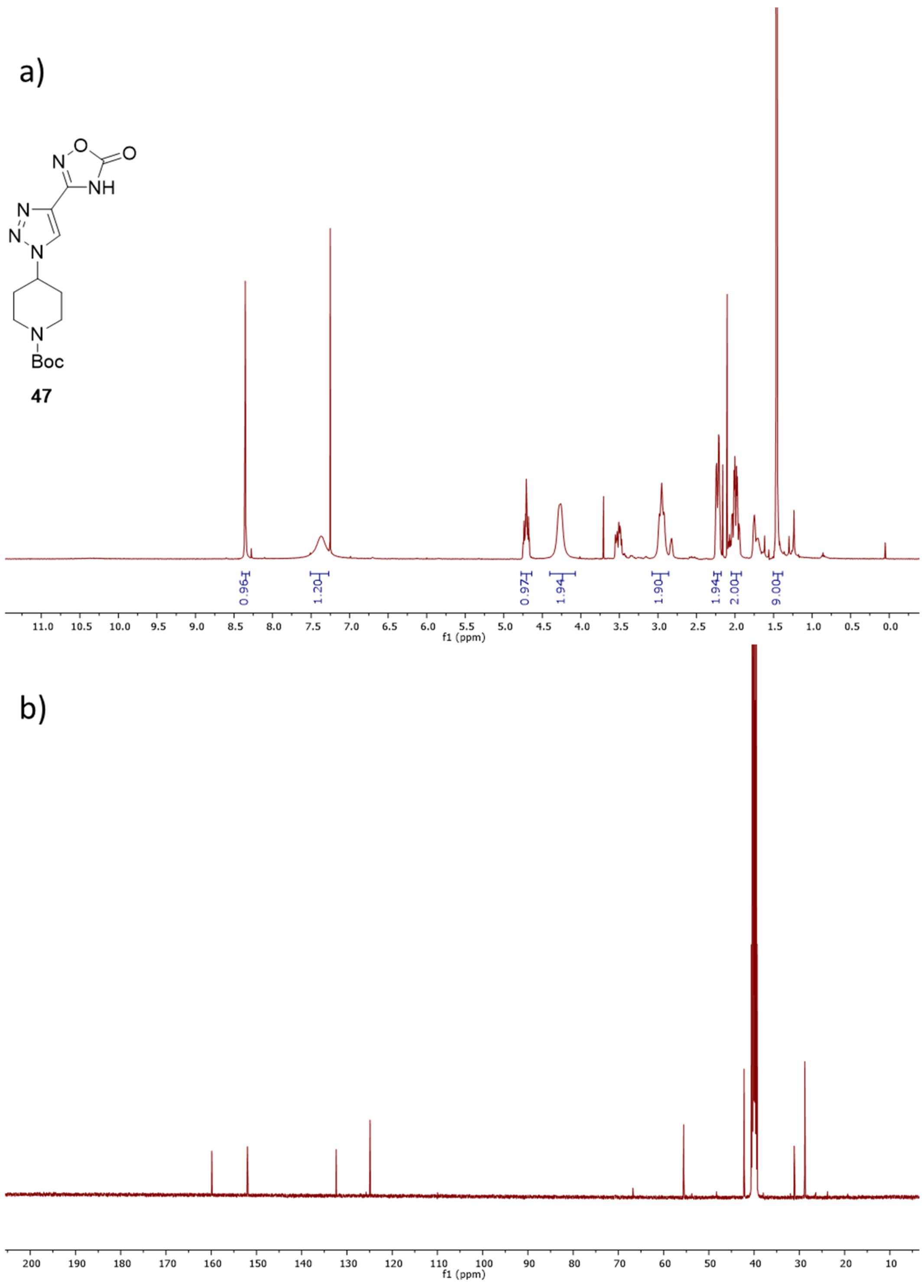

Figure S11. ^{1}H-NMR (a) and ^{13}C-NMR (b) spectra of **47** in *d$_6$*-DMSO.

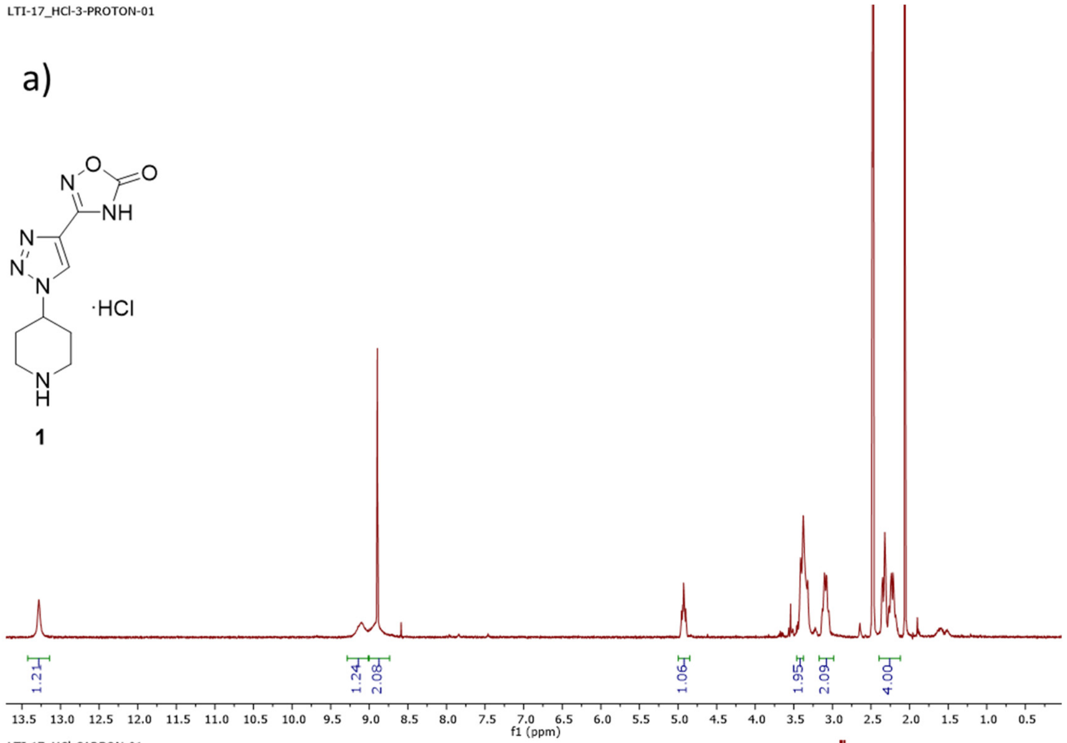

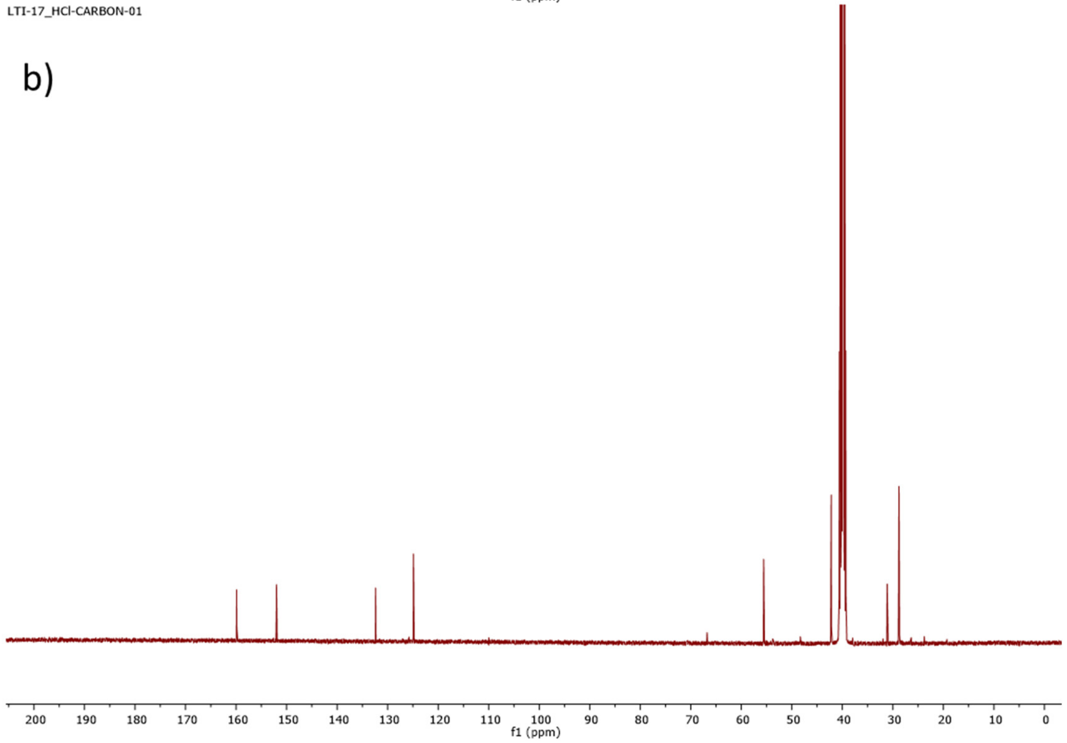

Figure S12. ^{1}H-NMR (a) and ^{13}C-NMR (b) spectra of **1** in *d*$_6$-DMSO.

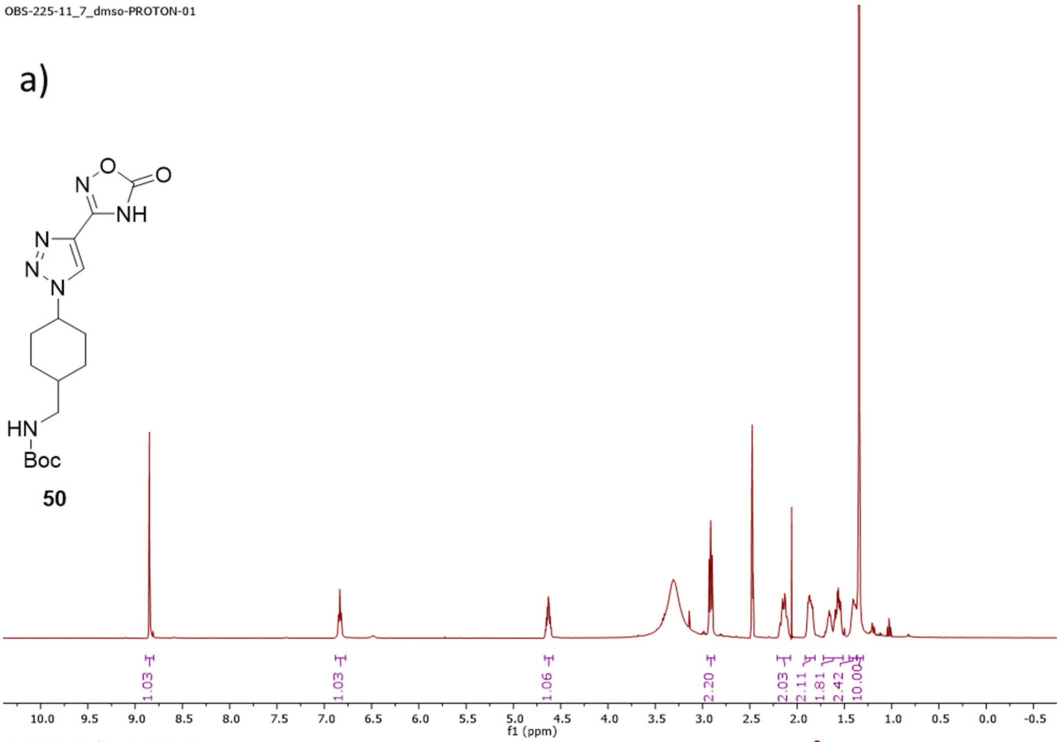

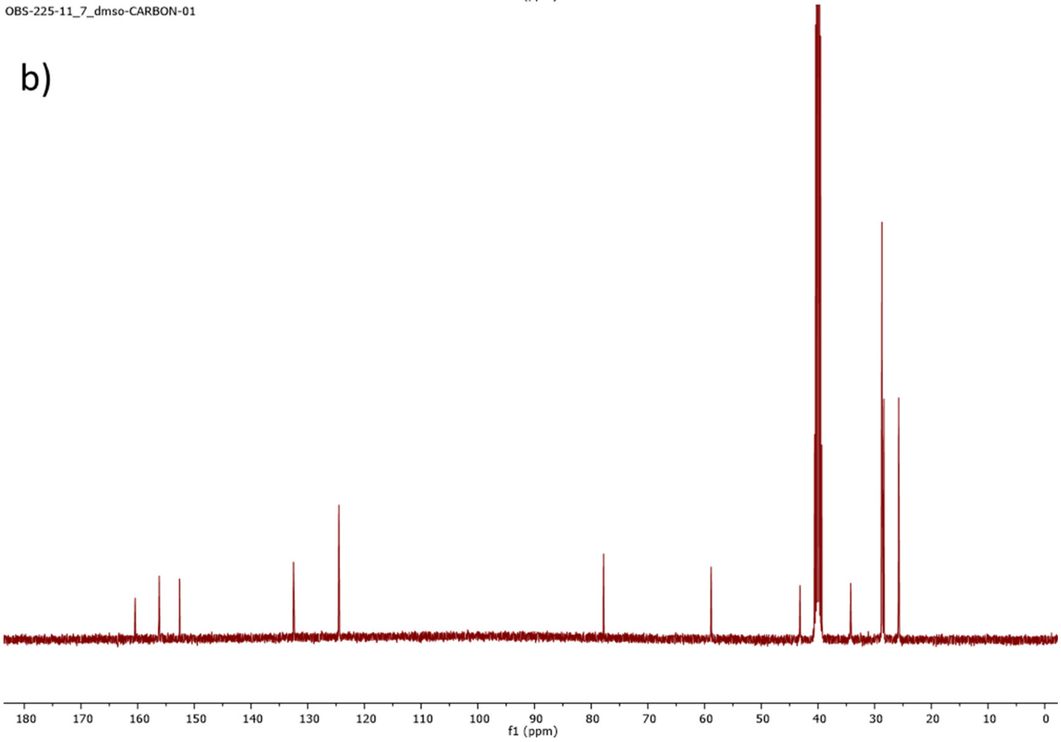

Figure S13. ^{1}H-NMR (a) and ^{13}C-NMR (b) spectra of **50** in d_6-DMSO.

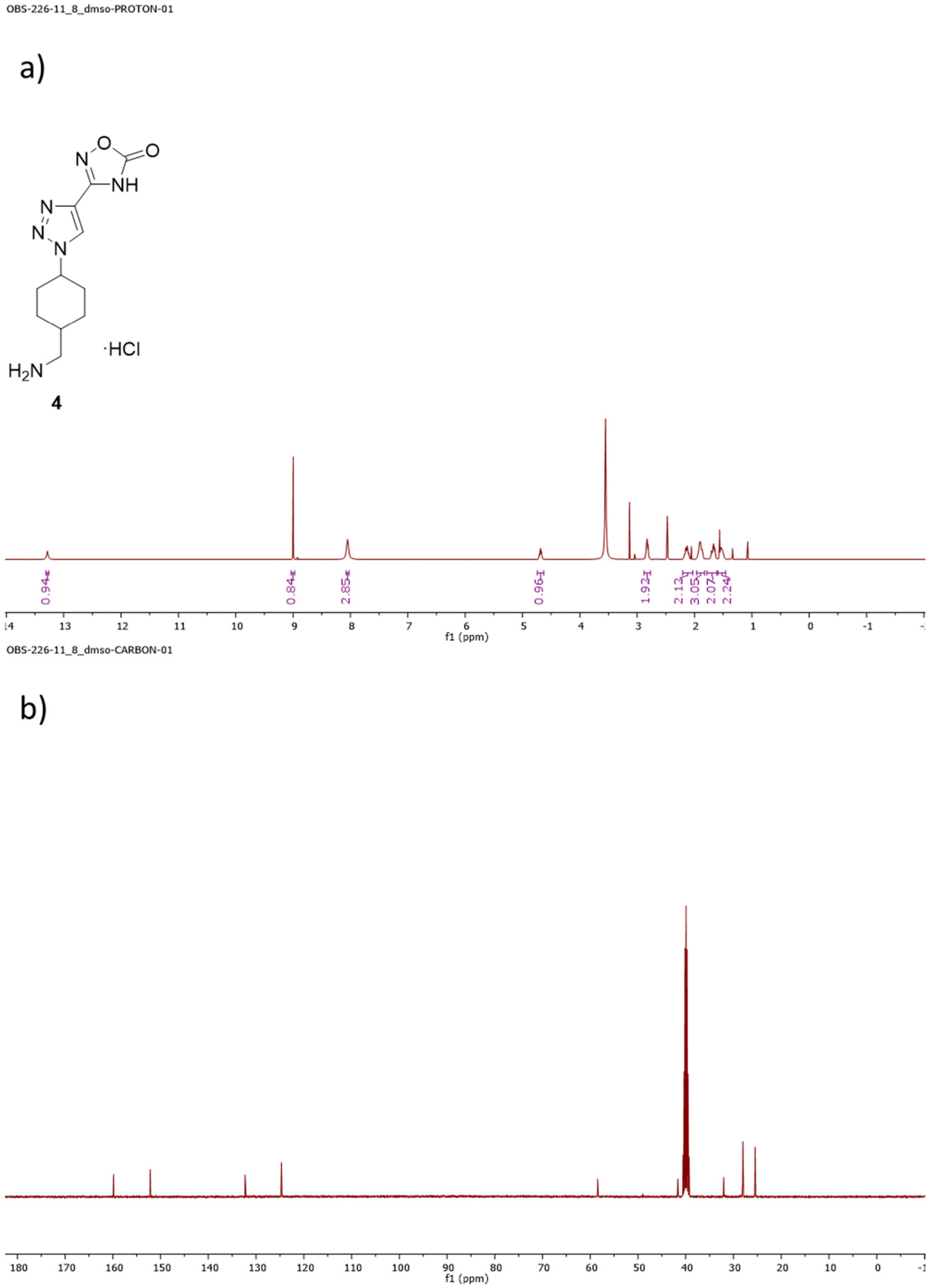

Figure S14. ^{1}H-NMR (a) and ^{13}C-NMR (b) spectra of **4** in *d*$_6$-DMSO.

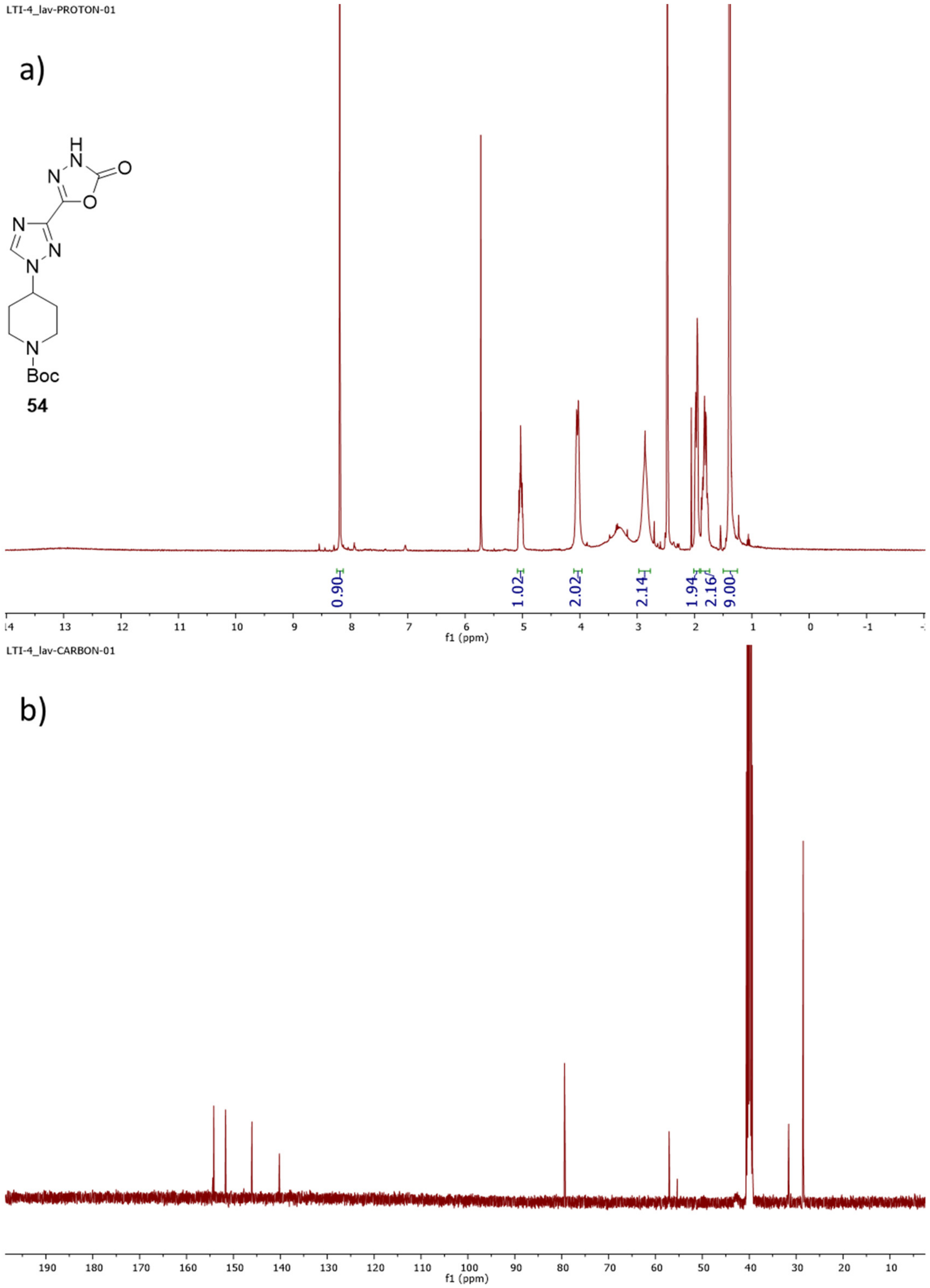

Figure S15. ^{1}H-NMR (a) and ^{13}C-NMR (b) spectra of **54** in *d₆*-DMSO.

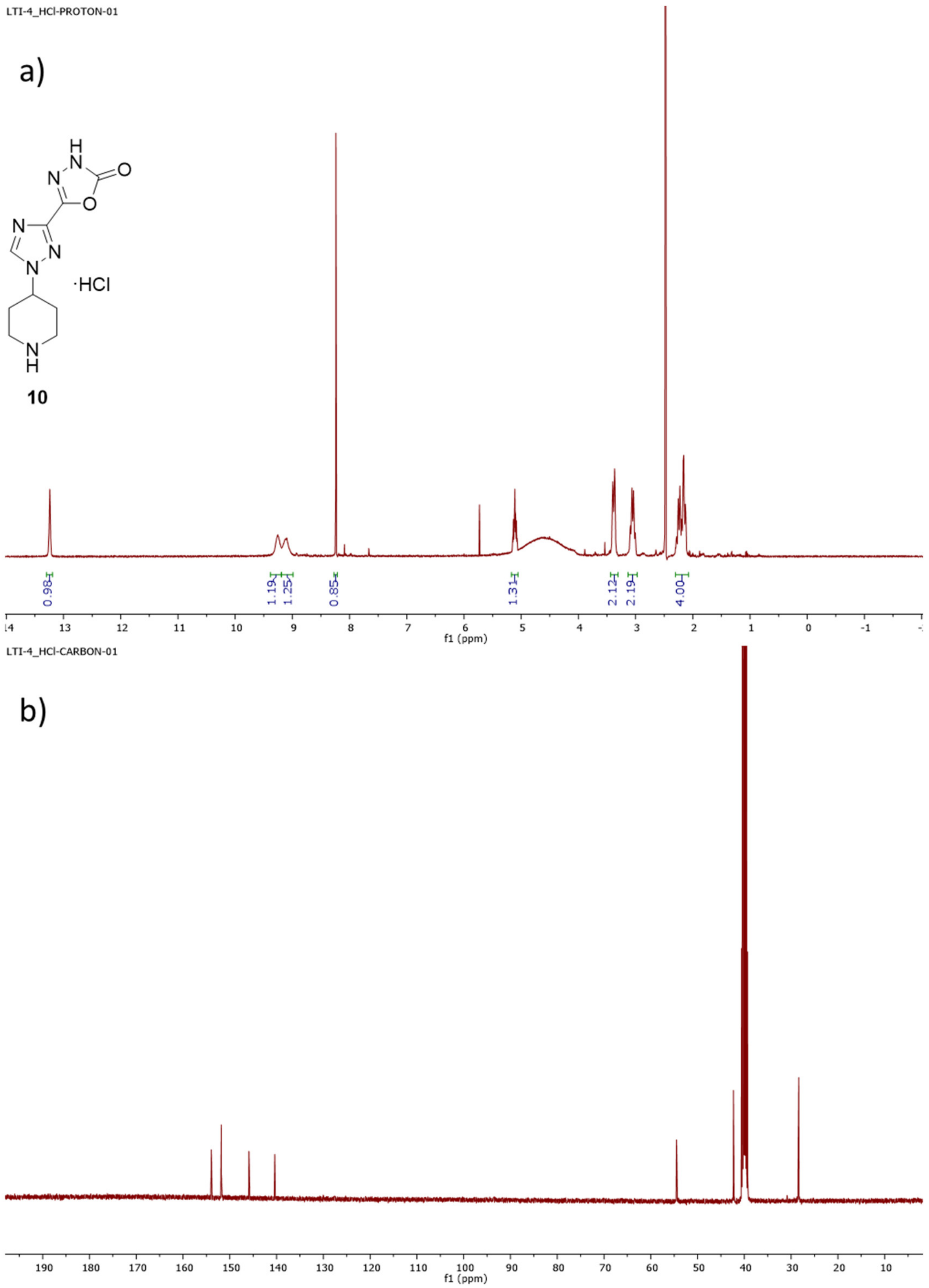

Figure S16. ^{1}H-NMR (a) and ^{13}C-NMR (b) spectra of **10** in *d$_6$*-DMSO.

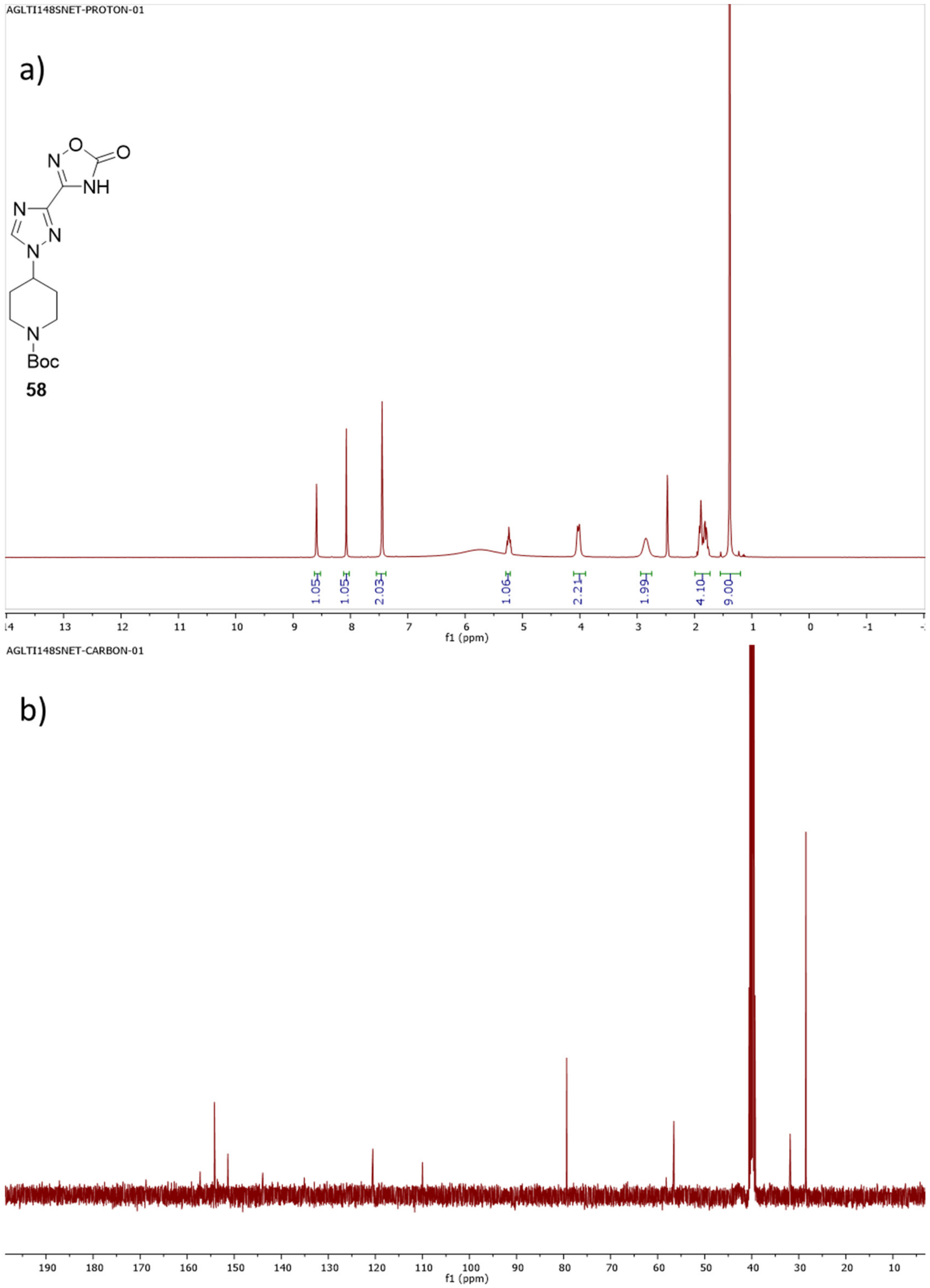

Figure S17. ^{1}H-NMR (a) and ^{13}C-NMR (b) spectra of **58** in d_6-DMSO.

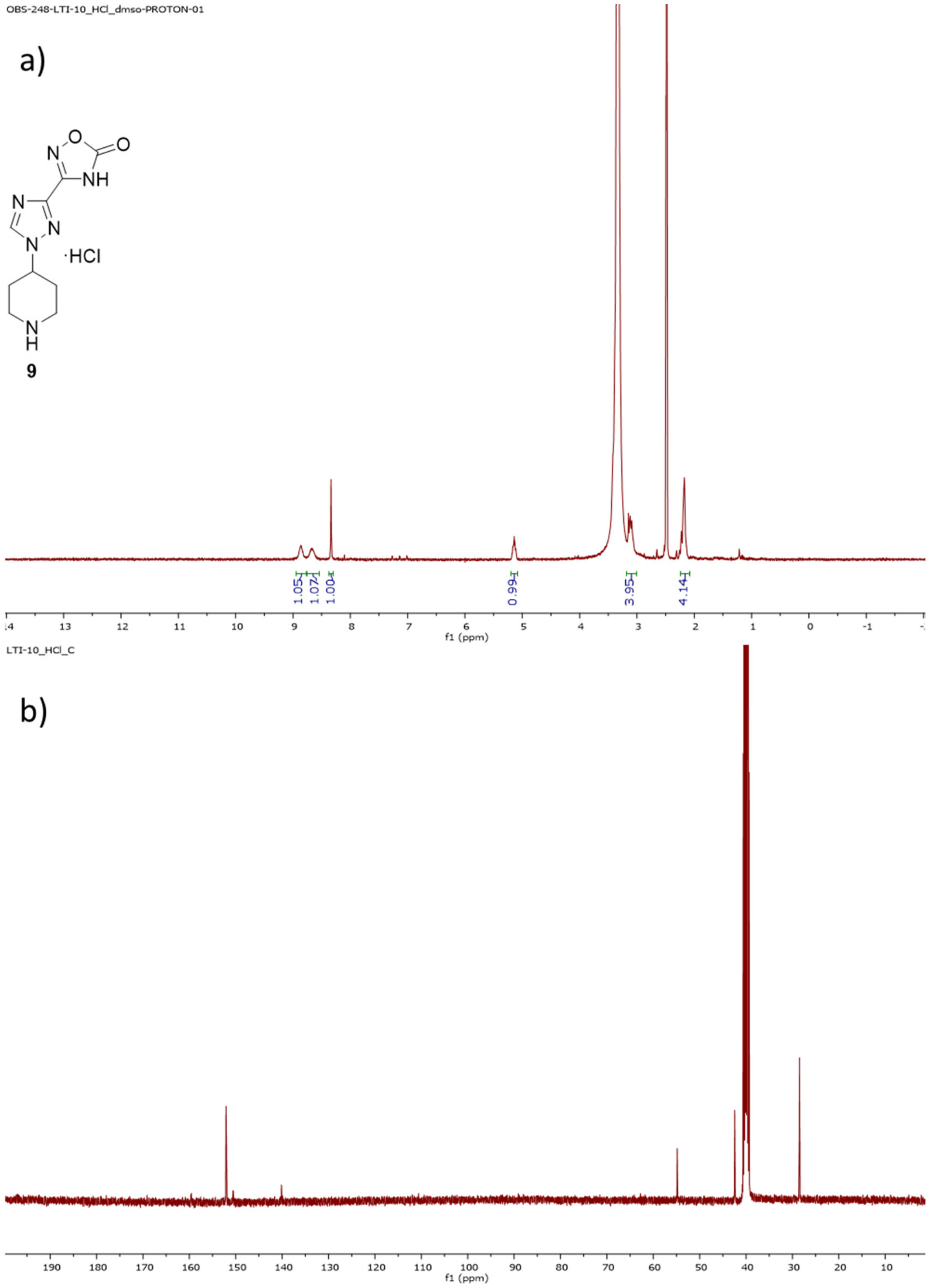

Figure S18. ^{1}H-NMR (a) and ^{13}C-NMR (b) spectra of **9** in *d*$_6$-DMSO.

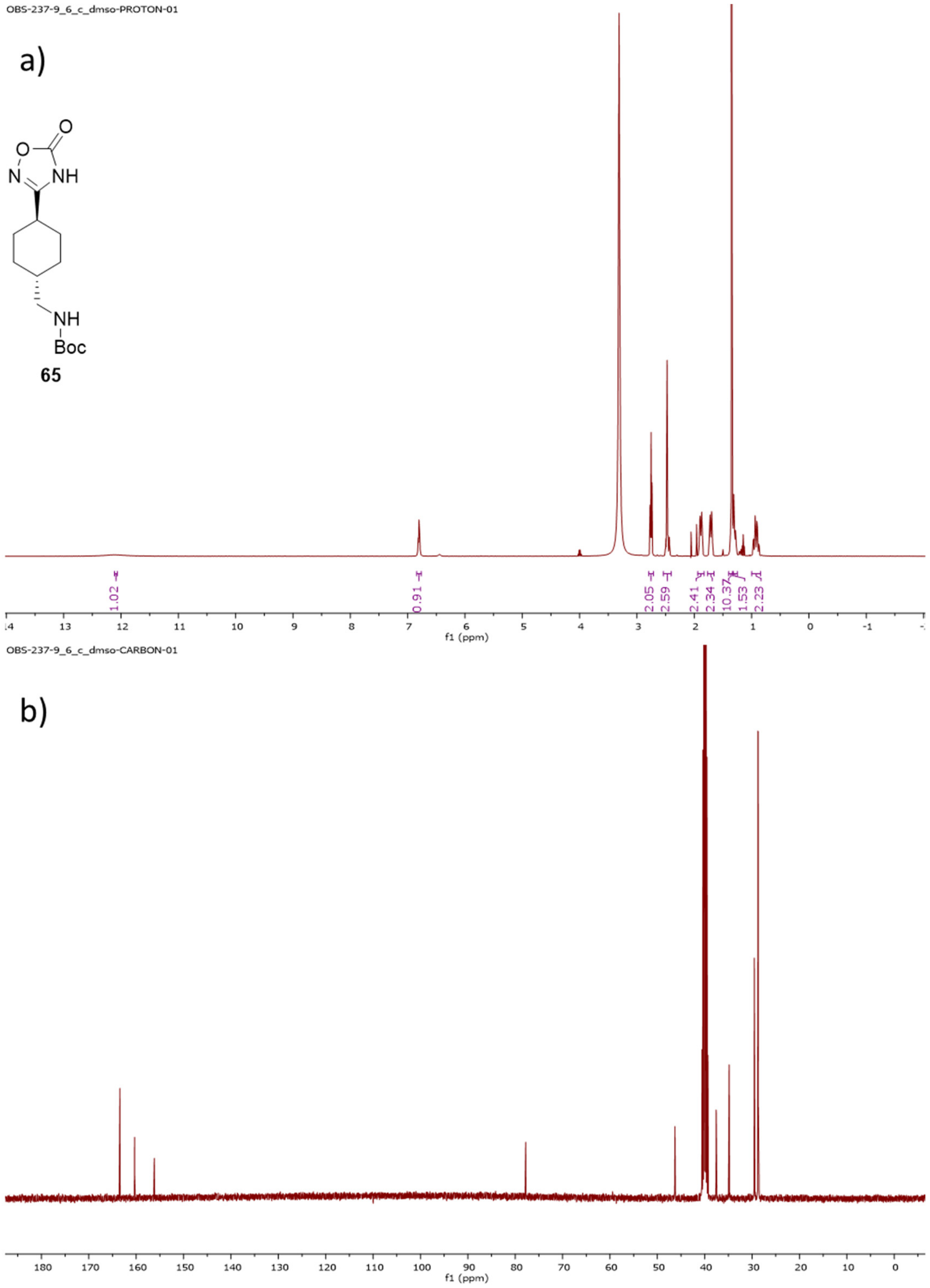

Figure S19. ^{1}H-NMR (a) and ^{13}C-NMR (b) spectra of **65** in *d_6*-DMSO.

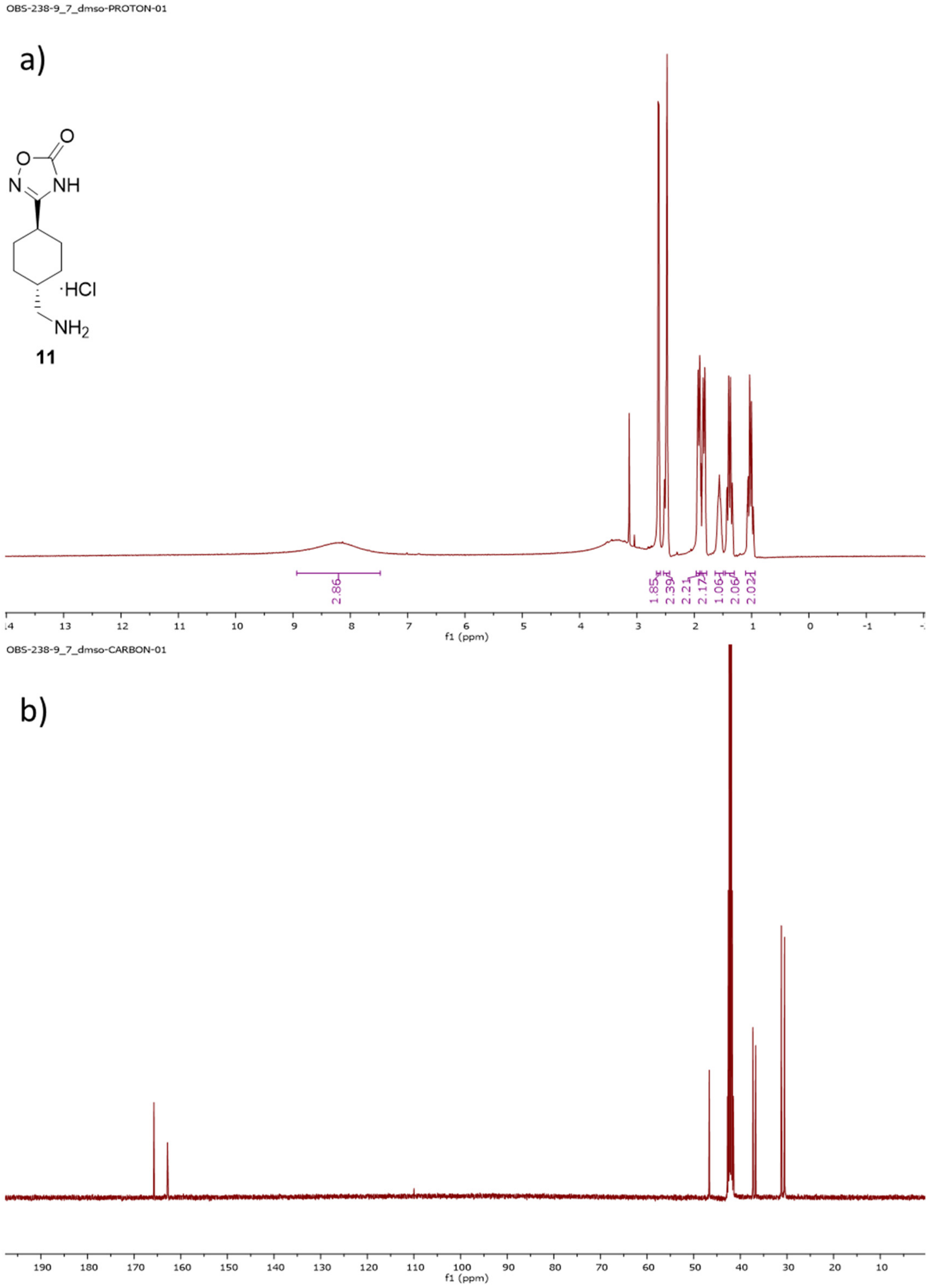

Figure S20. ^{1}H-NMR (a) and ^{13}C-NMR (b) spectra of **11** in *d$_6$*-DMSO.

III. Docking with $GABA_A$ receptor

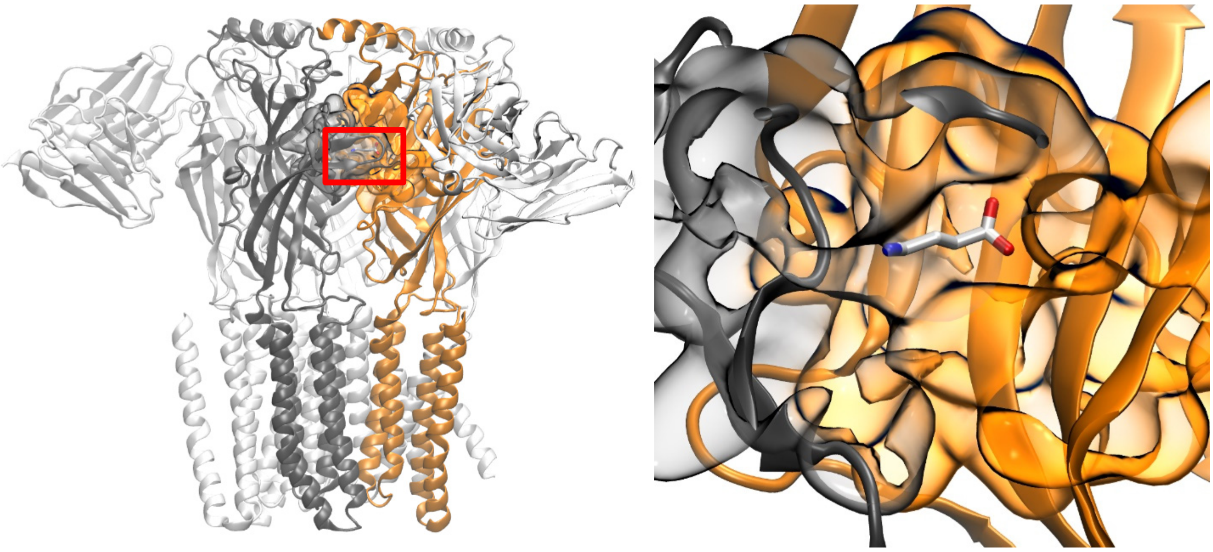

Figure S21. Full structure of the $GABA_A$ receptor (left) and expanded area of a binding pocket with GABA (right). $GABA_A$ receptor file obtained from PDB code 6d6u.

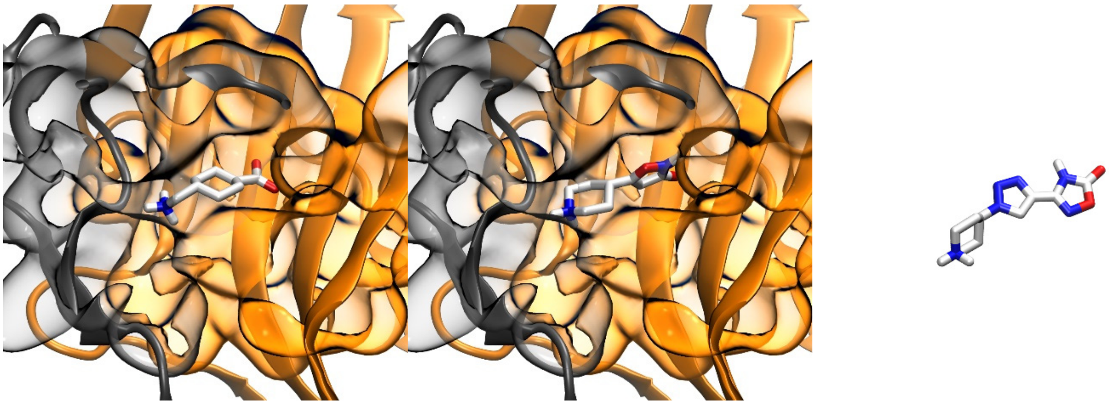

Figure S22. Binding poses with the GABA binding pocket for tranexamic acid (left) and 4-PIOL (center). Representation at same scale of compound **1** (right).
